# New Marine Actinobacteria Strain, *Micromonospora* sp. SH-82: Characterization, Specialized Metabolites and Biological Activities

**DOI:** 10.3390/microorganisms13092045

**Published:** 2025-09-02

**Authors:** Alexandre Le Loarer, Laurence Marcourt, Rémy Marcellin-Gros, Laurent Dufossé, Chatragadda Ramesh, Maile Anwesh, Jérome Bignon, Michel Frédérich, Allison Ledoux, Emerson Ferreira Queiroz, Jean-Luc Wolfender, Mireille Fouillaud, Anne Gauvin-Bialecki

**Affiliations:** 1 Laboratory of Chemistry and Biotechnology of Natural Products, Faculty of Science and Technology, University of La Réunion, 15 Avenue René Cassin, CS 92003, CEDEX 09, 97744 Saint-Denis, France; alexandre.le-loarer@univ-reunion.fr (A.L.L.); laurent.dufosse@univ-reunion.fr (L.D.); anne.bialecki@univ-reunion.fr (A.G.-B.); 2 Institute of Pharmaceutical Sciences of Western Switzerland, University of Geneva, CMU-Rue Michel-Servet 1, CH-1211 Geneva, Switzerland; laurence.marcourt@unige.ch (L.M.); remy.marcellin-gros@unige.ch (R.M.-G.); emerson.ferreira@unige.ch (E.F.Q.); jean-luc.wolfender@unige.ch (J.-L.W.); 3 School of Pharmaceutical Sciences, University of Geneva, CMU-Rue Michel-Servet 1, CH-1211 Geneva, Switzerland; 4 Biological Oceanography Division, National Institute of Oceanography (CSIR-NIO), Panaji 403004, Goa, India; chrameshpu@gmail.com; 5 Academy of Scientific and Innovative Research (AcSIR), Ghaziabad 201002, Uttar Pradesh, India; 6 DBT-Centre for Microbial Informatics, School of Life Sciences, University of Hyderabad, Gachibowli, Hyderabad 500046, Telangana, India; anwesh023@gmail.com; 7 Institute of Chemistry of Natural Substances (ICSN), Centre National de la Recherche Scientifique (CNRS) UPR 2301, Université Paris-Saclay, 1, av. de la Terrasse, 91198 Gif-sur-Yvette, France; jerome.bignon@cnrs.fr; 8 Pharmacognosy Laboratory, Department of Pharmacy, Centre Interfacultaire de Recherche sur le Médicament (CIRM), University of Liège, Campus du Sart-Tilman, Quartier Hôpital, Avenue Hippocrate, 15, B36, 4000 Liege, Belgium; m.frederich@uliege.be (M.F.); allison.ledoux@uliege.be (A.L.)

**Keywords:** marine actinobacteria, *Micromonospora*, specialized metabolites, biosynthetic gene cluster, molecular network, megalomicin, erythromycin

## Abstract

The study of various microorganisms isolated from an Indian Ocean sponge, *Scopalina hapalia* ML-263, led to the selection of a promising Actinobacteria strain, *Micromonospora* sp. SH-82. Genomic analysis identified this strain as a new species, revealing the presence of 23 biosynthetic gene clusters (BGCs), some of which are associated with the synthesis of specialized metabolites such as polyketides deriving from polyketide synthases (PKSs). The strain was cultivated under favorable conditions for the production of bioactive molecules, resulting in the isolation and identification of seven microbial metabolites. Three of them are potentially novel, two erythronolides and one erythromycin, all characterized by a rare C10–C11 double bond. Some of these compounds also display atypical conformations, forming hemiacetals or spiroacetals. Their identification was achieved through detailed chemical analyses (NMR and ESI^+^-HRMS). A molecular networking approach was employed to assess the presence of potentially novel molecules in the microbial crude extract, supported by the identification of isolated molecules. Four molecules (**1**, **2**, **3** and **5**) were evaluated for their cytotoxic activities against cancer cell lines (HCT-116 and MDA-MB-231) and the immortalized retinal pigment epithelial RPE1 cells. No activity was observed in the latter, suggesting a lack of toxicity toward healthy cells. Moreover, megalomicin C1 (3), one of the isolated compounds, showed interesting antiplasmodial activity against *Plasmodium falciparum* 3D7, with an IC_50_ of 6.37 ± 2.99 µM.

## 1. Introduction

In the face of major challenges such as cancer and malaria [1,2], the search for new therapeutic solutions is crucial. Microorganisms, both terrestrial and marine, are well-recognized sources of new drugs [3,4]. Among them, the genus *Micromonospora* particularly stands out as one of the most promising groups for the discovery of bioactive compounds [5]. *Micromonospora* are Gram-positive bacteria, generally aerobic, mesophilic, and saprophytic [6]. They typically form yellow-orange colonies due to their production of carotenoid pigments [5,7]. This genus is widely distributed in various habitats, such as soils, marine sediments, and extreme environments, highlighting their adaptability. *Micromonospora* can also be associated with macroorganisms such as sponges [8], and this marine environment contributes to the discovery of novel species [7]. This genus comprises 110 species [5], with genome sizes ranging from 5 to 10 Mb with a high Guanine+Cytosine (G+C) [9]. With biosynthetic gene clusters (BGCs) ranging from 7 to 30, these microorganisms demonstrate significant potential for the production of secondary metabolites of interest in fields such as pharmacology and biotechnology [5,7]. These natural products display diverse chemical structures and biological activities, such as gentamicin, an antibiotic [10], and diazepinomicin, which has anticancer properties [11].

Progress in genomics has highlighted the abundance of BGCs in *Micromonospora*, with more than 2000 predicted BGCs, while only around 400 molecules have been isolated [5]. This disparity is due to the presence of numerous predicted BGCs that remain unexpressed under standard laboratory conditions [12]. One major obstacle to harnessing this metabolic potential is the activation of these BGCs, whose expression is often tightly regulated, condition-dependent, or completely repressed under standard laboratory conditions [12,13]. To overcome this challenge, several strategies have been developed, including co-culturing with inducing microorganisms [14], promoter engineering to activate gene cluster expression [15], and heterologous expression of BGCs in optimized host strains [16].

This article describes the study of a new strain, *Micromonospora* sp. SH-82, isolated from an Indian Ocean sponge, *Scopalina hapalia* ML-263 [17]. Using an adapted selection method and improved culture conditions [18,19], this strain was cultivated for the production of valuable secondary metabolites. This article focuses on the study of its metabolome using bioinformatics tools, leading to the isolation and identification of seven secondary metabolites, belonging to families of bioactive molecules such as megalomicins, erythromycins, and erythronolides. Among them, three compounds are not described in the literature. These new compounds (**5**–**7**) likely result from acid-mediated hydrolysis and/or rearrangement processes, leading to unusual hemiacetal or spiroacetal conformations, and are distinguished by a rare double bond at position C10–C11 within this type of structure. Compounds **1**–**3** and **5** were tested for cytotoxic activity, while only megalomicin C1 (compound **3**) was evaluated for antiplasmodial activity and showed a promising effect. In addition, the crude extract was evaluated for both activities. This study also provides morphological identification and genomic information about this species. This study not only assessed the strain’s capacity to produce bioactive secondary metabolites but it also paves the way for the exploration of new potential molecules to be isolated. It highlights the relevance of a multidisciplinary approach to exploring the biosynthetic potential of microorganisms. Genetic analysis revealed a strain rich in BGCs, suggesting increased production of bioactive metabolites. Through innovative culture strategies and advanced analytical techniques, several compounds of interest, including some in low abundance, were identified, paving the way for new biological applications.

## 2. Materials and Methods

### 2.1. General Experimental Procedures

Macroscopic and microscopic observation was performed on a stereomicroscope (S Apo, Leica, Wetzlar, Germany) and a microscope (DM1000, Leica, Wetzlar, Germany) coupled with a camera (MC170HD, Leica, Wetzlar, Germany). Metabolite profiling and purity control were performed via UHPLC-PDA-ELSD-MS on an ACQUITY UPLC I-Class system (Waters, Milford, MA, USA) equipped with an ACQUITY UPLC photodiode array detector (Waters, Milford, MA, USA), a SEDEX LT-ELSD 100LT evaporative light-scattering detector (Sedere, Alfortville, France), and a single-quadrupole detector (QDa) using heated electrospray ionization. Agilent Technologies 1260 HPLC system (Agilent Technologies, Santa Clara, CA, USA) equipped with a solvent degasser (G4225A), a binary pump (G1312B), a sample manager (G1329B), a column chamber (G1316A) coupled with a diode array detector (G4212B) and a Sedex 85 LT-evaporative light-scattering detector (Sedere, Alfortville, France) was used for optimized metabolite separation. The semi-preparative Shimadzu HPLC system (Shimadzu, Kyoto, Japan) equipped with an LC-20AP module pump, an SPD-20A UV/VIS module, a 7725I Rheodyne^®^ switch valve, and an FRC-10A fraction collector (Shimadzu, Kyoto, Japan), connected to a Sedex-FP LT-evaporative light-scattering detector (Sedere, Alfortville, France) was used for the purification of metabolites. ESI^+^-HRMS/MS data were obtained on a Waters UHPLC system coupled with a Thermo Scientific Orbitrap mass spectrometer (Thermo Scientific^®^, Bremen, Germany), using ion source with a heated electrospray ionization (HESI-II) probe. NMR spectroscopic data were acquired on a Bruker Avance Neo 600 MHz NMR spectrometer equipped with a QCI 5 mm Cryoprobe and a SampleJet automated sample changer (Bruker BioSpin, Rheinstetten, Germany). Spectra were recorded in CD_3_OD without solvent suppression at a fixed temperature of 298 K. Chemical shifts are reported in parts per million (δ) using the residual CD_3_OD signal (δ_H_ 3.31; δ_C_ 49.0) as internal standards for ^1^H and ^13^C NMR, respectively, and coupling constants (*J*) are reported in Hz. Complete assignments were obtained based on 2D-NMR experiments (COrrelation SpectroscopY (COSY), Rotating frame Overhauser Enhancement SpectroscopY (ROESY), Heteronuclear Single-Quantum Correlation (HSQC) and Heteronuclear Multiple-Bond Correlation (HMBC)).

### 2.2. Biological Material and Strain Culture

From the sponge *Scopalina hapalia* ML-263, collected on the island of Mayotte, a collection of 124 microbial strains was isolated [17]. This study focuses on the *Micromonospora* sp. SH-82 strain.

The strains were all preserved in storage cryotubes and then reactivated before the preparation of pre-cultures [18]. The A1BFe+C medium containing 33 g sea salts (Instant Ocean 16 kg, Aquarium system, Sarrebourg, France), 10 g soluble starch (ref. 417587, BD Difco, Le Pont de Claix, France), 4 g yeast extract (ref. 212750, BD Bacto, Le Pont de Claix, France), 2 g peptone (ref. 211820, BD Bacto, Le Pont de Claix, France), 1 g CaCO_3_ (ref. 433185, Carlo Erba, Val de Reuil, France), 100 mg KBr (ref. 470735, Carlo Erba, Val de Reuil, France), 40 mg Fe_2_(SO_4_)_3_ (ref. 451926, Carlo Erba, Val de Reuil, France), 20 agar (ref. 281210, BD Difco, Le Pont de Claix, France) and QSP distilled water to obtain 1 L of final medium was used for all culture stages. The bacterial strains were revived on solid medium A1 for a period ranging from 7 to 15 days. Subsequently, two Petri dishes, completely covered with a dense microbial culture, were scraped to prepare a bacterial suspension in 25 mL of artificial seawater (ASW) (sea salts 33 g/L). Then, 10 mL of this suspension was inoculated into 100 mL of medium A1 to perform pre-cultures, which were incubated for 7 days at 21 °C.

The solid culture of *Micromonospora* sp. SH-82 was generated by introducing 12.5 mL of pre-culture with 25 g of amberlite resin XAD-16 (ref. MFCD00145831, Sigma Aldrich, St. Louis, MI, USA) on a 25 × 25 cm culture Petri dish (ref. 240835, Nunc Thermo Fisher, Waltham, MA, USA) containing 250 mL of solid A1BFe+C medium. A total of 4 L of this solid culture was prepared and incubated for 21 days at 28° in MIR-154 PE thermostatic oven (PHC, Etten-Leur, The Netherlands). Additionally, a culture blank was prepared without microorganisms.

### 2.3. Genomic Analysis, Observation, and Identification

Petri dish cultures of *Micromonospora* sp. SH-82 strain, cultivated on A1BFe+C medium for 21 days, were produced and used for observation. A stereomicroscope (S Apo, Leica, Wetzlar, Germany) and a microscope (DM1000, Leica, Wetzlar, Germany) coupled with a camera (MC170HD, Leica, Wetzlar, Germany) were used. Macroscopic observation was performed directly on the Petri dish, and microscopic observation was performed on a bacterial suspension after Gram coloration.

The Petri dishes were also sent to the society Genoscreen for complete genome sequencing. Raw reads were obtained from the Illumina NovaSeq platform and Oxford Nanopore GridION platform. The Illumina paired-end reads were quality checked with FastQC v0.12.1 [20] and trimmed using BBDuk v39.01 [21] to remove adaptor sequences and retain bases above a quality score of 20. Similarly, nanopore raw reads were filtered using nanoplot v1.20 [22] and porechop v0.2.4 [23] to remove low-quality sequences. The filtered short and long reads were assembled into contigs in a hybrid de novo fashion using Unicycler v0.5.0 [24]. The quality of the assembly was verified using BUSCO v5.5.0 [25] and CheckM v1.2.2 [26]. Barrnap v0.9 [27] was used to extract ribosomal RNA genes, and the extracted 16S rRNA gene was searched against the non-redundant nucleotide (nr-nt) database at NCBI using the BLASTn web suite (BLAST+ v2.15.0) [28]. The 16S rRNA genes were extracted from the downloaded genomes and were used to obtain multiple sequence alignments with MAFFT v.7.520 [29]. Phylogenetic trees were constructed using RAxML v8.2.12 [30] with the GTRGAMMA model, 1000 bootstrap replicates, and the corresponding genes of *Salinispora tropica* strain CNB440 as outgroups. Genome-to-genome pairwise comparisons were made by calculating the average nucleotide identity (ANI) values with fastANI v1.33 [31], and a comparison plot was constructed with ANIclustermap v1.3.0 [32]. Gene annotation was performed using the online RAST server (RASTtk v1.30) [33] as well as Prokka v1.14.6 [34] on a local machine. Secondary metabolite gene clusters in the genome were identified using antiSMASH tool v6 [35], and the distribution of BGCs was visualized in a pie chart.

### 2.4. Extract Preparation and Purification

After 21 days of incubation, 25 g of resin and the associated biomass were recovered and extracted together with 100 mL of ethyl acetate (EtOAc) (ref. 448252 grade RPE, CarloErba, Val de Reuil, France) for 4 h, in accordance with this protocol [19]. To perform delipidation of the crude extract, it was evaporated and dissolved in a solution containing a mixture of MeOH/H_2_O/hexane (7/3/10). The MeOH/H_2_O phase of interest was washed with an equal volume of hexane three times and then evaporated to obtain a delipidated dry extract (±250 mg). This step allowed for the removal of highly apolar compounds such as lipids, enriching the extract in polar and semi-polar metabolites such as macrolides and polyketides, which were targeted for chemical and biological analyses, as well as for fractionation aimed at their isolation. This delipidated extract was then used for all subsequent analyses described in this study.

The crude extract and purified metabolites were analyzed via UHPLC-PDA-ELSD-MS using an Acquity BEH (Bridget Ethylene Hybrid) C18 analytical column (50 × 2.1 mm; 1.7 μm) (Waters™, Milford, MA, USA). The analyses were conducted with a solvent system consisting of (A) water (Optima™ LC/MS Grade, Fisher Chemical™, Portsmouth, NH, USA) with 0.1% formic acid, employing a linear gradient from 5 to 100% B over 7 min followed by an isocratic step at 100% B for one minute, with a flow rate set at 600 μL/min. Instrument control, data acquisition, and processing were performed using Masslynx^®^ software v4.1 (Waters^®^ Milford, MA, USA). The analytical conditions were transferred using a gradient transfer method [36] to an Agilent HPLC system equipped with an analytical XBridge^®^ BEH amide column (250 × 4.6 mm, 5 µm) (Waters™, Milford, MA, USA) for optimizing metabolite separation. To maintain separation resolution on semi-preparative Shimadzu HPLC system (Shimadzu, Kyoto, Japan), the sample (250 mg) was dry-loaded on the precolumn [37]. The separation was performed on a puriFlash^®^ C18-HP column (250 × 19 mm, 5 µm) (Interchim, Montluçon, France). The solvent system used was a mixture of (A) filtered H_2_O (Elga Purelab^®^ Ultra, ELGA LabWater, Lane End, UK) and (B) HPLC-grade acetonitrile (ACN) (Fisher Chemical™, Hampton, NH, USA), each containing 0.1% formic acid. The separation used an optimized gradient: 10% to 50% of B over 61.4 min, followed by 50% to 100% over 20 min and then held at 100% for 20 min. The flow rate was set at 17 mL/min. After collection, similar fractions, each comprising at least six individual fractions, were combined, evaporated to dryness using an EZ-2 Elite solvent evaporator (GENEVAC™, Stone Ridge, NY, USA). The resulting samples were then analyzed via UHPLC-ESI-HRMS/MS to assess their purity.

This purification led to the isolation of 7 pure compounds, with a purity estimated by NMR to be above 85% and obtained in small quantities (ranging from 0.2 to 2 mg): 1.4 mg of compound **1**, 2 mg of compound **2**, 1.5 mg of compound **3**, 0.5 mg of compound **4**, 1.4 mg of compound **5**, 1.8 mg of compound **6**, and 0.2 mg of compound **7**. These pure molecules were then identified on spectral interpretation and tested for their biological activities according to the quantity available.

### 2.5. Description of the Isolated Compounds

A detailed description of the chemical shifts of the isolated compounds is provided in Tables 1–3, located in Section 3.2.1.

Erythronolide B (**1**): For ^1^H- and ^13^C-NMR data, see Table 1, Table S1, Figures S5–S9. ESI^+^-HRMS *m*/*z* 385.2583 [M-H_2_O+H]^+^ (calcd for C_21_H_37_O_6_, 385.2590, Δ1.8 ppm); for HRMS data, see Figure S10.

6-desoxyerythronolide B (**2**): ^1^H NMR (CD_3_OD, 600 MHz) Δ0.93 (3H, t, *J* = 7.4 Hz, H_3_-15), 0.93 (3H, d, *J* = 7.1 Hz, 12-CH_3_), 0.96 (3H, d, *J* = 6.8 Hz, 10-CH_3_), 0.99 (1H, overlapped, H-7″), 1.05 (3H, d, *J* = 6.4 Hz, 8-CH_3_), 1.06 (3H, d, *J* = 6.9 Hz, 4-CH_3_), 1.10 (3H, d, *J* = 7.0 Hz, 6-CH_3_), 1.23 (3H, d, *J* = 6.7 Hz, 2-CH_3_), 1.54 (1H, dqd, *J* = 14.0, 7.3, 4.4 Hz, H-14″), 1.70 (1H, dqd, *J* = 10.2, 7.1, 1.3 Hz, H-12), 1.80 (2H, overlapped, H-4, H-6), 1.80 (1H, pd, *J* = 7.4, 2.6 Hz, H-14′), 1.89 (1H, ddd, *J* = 14.2, 6.1, 3.5 Hz, H-7′), 2.69 (1H, dqd, *J* = 12.8, 6.4, 3.5 Hz, H-8), 2.77 (1H, dq, *J* = 10.3, 6.7 Hz, H-2), 2.91 (1H, qd, *J* = 6.7, 2.0 Hz, H-10), 3.67 (1H, dd, *J* = 10.3, 1.3 Hz, H-3), 3.69 (1H, dd, *J* = 6.1, 3.1 Hz, H-5), 3.79 (1H, dd, *J* = 10.2, 2.2 Hz, H-11), 5.30 (1H, ddd, *J* = 9.6, 4.4, 1.2 Hz, H-13); ^13^C NMR (CD_3_OD, 151 MHz) Δ6.8 (10-CH_3_), 8.5 (4-CH_3_), 9.7 (12-CH_3_), 10.9 (CH_3_-15), 14.6 (8-CH_3_), 15.3 (2-CH_3_), 19.6 (6-CH_3_), 26.7 (CH_2_-14), 37.0 (CH_2_-7), 37.3 (CH-6), 40.6 (CH-4), 42.4 (CH-12), 43.5 (CH-8), 44.7 (CH-10), 45.3 (CH-2), 72.3 (CH-11), 77.1 (CH-13), 77.4 (CH-3), 77.9 (CH-5), 178.9 (C-1), 216.7 (C-9); ^1^H NMR (CDCl_3_, 600 MHz) Δ0.89 (3H, d, *J* = 7.0 Hz, 12-CH_3_), 0.93 (3H, t, *J* = 7.4 Hz, H_3_-15), 1.02 (3H, d, *J* = 6.8 Hz, 10-CH_3_), 1.05 (3H, d, *J* = 6.3 Hz, 6-CH_3_), 1.05 (3H, d, *J* = 6.3 Hz, 8-CH_3_), 1.07 (3H, d, *J* = 7.0 Hz, 4-CH_3_), 1.25 (1H, m, H-7″), 1.30 (3H, d, *J* = 6.8 Hz, 2-CH_3_), 1.52 (1H, dqd, *J* = 14.4, 7.4, 4.1 Hz, H-14′), 1.67 (1H, overlapped, H-7′), 1.73 (1H, overlapped, H-12), 1.82 (1H, overlapped, H-14″), 1.86 (1H, overlapped, H-4), 2.03 (1H, overlapped, H-6), 2.62 (1H, dqd, *J* = 9.0, 6.4, 3.6 Hz, H-8), 2.76 (1H, qd, *J* = 6.8, 2.4 Hz, H-10), 2.79 (1H, dq, *J* = 10.3, 6.8 Hz, H-2), 3.68 (1H, dd, *J* = 10.2, 2.4 Hz, H-11), 3.92 (1H, d, *J* = 10.3 Hz, H-3), 4.01 (1H, dd, *J* = 5.2, 2.3 Hz, H-5), 5.15 (1H, ddd, *J* = 9.5, 4.1, 1.5 Hz, H-13); ^13^C NMR (CDCl_3_, 151 MHz) Δ6.4 (10-CH_3_), 7.1 (4-CH_3_), 9.3 (12-CH_3_), 11.0 (CH_3_-15), 13.4 (8-CH_3_), 15.0 (2-CH_3_), 16.7 (6-CH_3_), 25.9 (CH_2_-14), 35.7 (CH-6), 37.6 (CH-4), 37.8 (CH_2_-7), 39.4 (CH-8), 40.6 (CH-12), 43.6 (CH-10), 44.2 (CH-2), 71.0 (CH-11), 76.5 (CH-13), 76.7 (CH-5), 79.8 (CH-3), 178.6 (C-1), 213.6 (C-9); NMR data see Table S2, Figure S11–S15; ESI^+^-HRMS *m*/*z* 369.2633 [M-H_2_O+H]^+^ (calcd for C_21_H_37_O_5_, 369.2636, Δ0.8 ppm); HRMS data see Figure S16.

megalomicin C1 (**3**): For ^1^H- and ^13^C-NMR data, see Table 3, Table S3, Figure S17–S22. ESI^+^-HRMS *m*/*z* 961.5847 [M+H]^+^ (calcd for C_48_H_85_O_17_N_2_, 961.5843, Δ0.4 ppm); for HRMS data, see Figure S23.

6,9-hemiacetal-8,9-anhydroerythonolide B (**4**): ^1^H NMR (CD_3_OD, 600 MHz) Δ0.87 (3H, t, *J* = 7.4 Hz, H_3_-15), 0.93 (3H, d, *J* = 7.5 Hz, 12-CH_3_), 0.95 (3H, d, *J* = 7.0 Hz, 4-CH_3_), 1.07 (3H, d, *J* = 7.1 Hz, 10-CH_3_), 1.16 (3H, d, *J* = 6.7 Hz, 2-CH_3_), 1.34 (3H, s, 6-CH_3_), 1.48 (1H, dqd, *J* = 14.0, 7.4, 4.9 Hz, H-14″), 1.55 (3H, t, *J* = 1.3 Hz, 8-CH_3_), 1.59 (1H, dq, *J* = 9.2, 7.5 Hz, H-12), 1.69 (1H, ddq, *J* = 14.0, 9.5, 7.4 Hz, H-14′), 2.06 (1H, qd, *J* = 7.0, 1.4 Hz, H-4), 2.07 (1H, dq, *J* = 15.7, 1.3 Hz, H-7″), 2.61 (1H, dq, *J* = 9.2, 7.1 Hz, H-10), 2.66 (1H, dq, *J* = 10.4, 6.7 Hz, H-2), 2.81 (1H, dq, *J* = 15.7, 1.3 Hz, H-7′), 3.49 (1H, dd, *J* = 10.4, 1.4 Hz, H-3), 3.56 (1H, t, *J* = 9.2 Hz, H-11), 3.56 (1H, s, H-5), 5.41 (1H, dd, *J* = 9.5, 4.9 Hz, H-13); ^13^C NMR (CD_3_OD, 151 MHz) Δ7.2 (4-CH_3_), 9.2 (12-CH_3_), 10.8 (CH_3_-15), 12.5 (8-CH_3_), 14.6 (2-CH_3_), 16.3 (10-CH_3_), 27.0 (CH_2_-14), 28.9 (Me6), 35.4 (CH-10), 36.2 (CH-4), 43.3 (CH_2_-7), 44.9 (CH-2), 47.6 (CH-12), 71.9 (CH-11), 78.5 (CH-13), 82.8 (CH-3), 83.1 (CH-5), 85.1 (C-6), 102.5 (C-8), 152.5 (C-9), 177.5 (C-1); NMR data, see Table S4, Figures S24–S29; ESI^+^-HRMS *m*/*z* 385.2568 [M+H]^+^ (calcd for C_21_H_37_O_6_, 385.2585, Δ4.4 ppm); HRMS data, see Figure S30.

6,9-hemiacetal-9-O-methyl-10,11-anhydroerythronolide B (**5**): For ^1^H- and ^13^C-NMR data, see Table 1, Table S5, Figures S31–S36. ESI^+^-HRMS *m*/*z* 399.2728 [M+H]^+^ (calcd for C_22_H_39_O_6_, 399.2746, Δ4.5 ppm); for HRMS data, see Figure S37.

6,9-hemiacetal-9-hydroxy-10,11-anhydroerythronolide B (**5.1**): For ^1^H- and ^13^C-NMR data, see Table 1, Table S5, Figures S38–S42. ESI-HRMS *m*/*z* 367.2474 [M-H_2_O+H]^+^ (calcd for C_21_H_35_O_5_, 367. 2482, Δ2.2 ppm); for HRMS data, see Figure S43.

8-*epi*-10,11-anhydroerythronolide B (**5.2**): For ^1^H- and ^13^C-NMR data, see Table 2, Table S5, Figures S38–S42. ESI^+^-HRMS *m*/*z* 367.2474 [M-H_2_O+H]^+^ (calcd for C_21_H_35_O_5_, 367.2482, Δ2.2 ppm); for HRMS data, see Figure S43.

10,11-anhydroerythronolide B (**6**): For ^1^H- and ^13^C-NMR data, see Table 2, Figures S44–S49. ESI^+^-HRMS *m*/*z* 367.2474 [M-H_2_O+H]^+^ (calcd for C_21_H_35_O_5_, 367.2482, Δ2.2 ppm); for HRMS data, see Figure S50.

3″,4″-di-O-acetyl-9-deoxo-6,12-dideoxy-6,9:9,12-diepoxyerythromycin D (**7**): For ^1^H- and ^13^C-NMR data, see Table 3, Figures S51–S55. ESI^+^-HRMS *m*/*z* 768.4528 [M+H]^+^ (calcd for C_40_H_66_O_13_N, 768.4529, Δ0.1 ppm); for HRMS data, see Figure S56.

### 2.6. UHPLC-ESI-HRMS/MS Analyses

Microbial crude extracts and isolated metabolites were analyzed via UHPLC-ESI-HRMS/MS on a Waters Acquity UPLC I-Class system (Waters^®^, Milford, MA, USA) coupled with a Thermo Scientific Orbitrap Exploris 120 mass spectrometer (Thermo Scientific^®^, Bremen, Germany), using a Thermo Scientific OptaMax NG ion source with a heated electrospray ionization (HESI-II) probe.

Chromatographic experiment was performed on a Acquity BEH C18 analytical column (50 × 2.1 mm; 1.7 μm) (ref. 186002350, Waters™, Milford, MA, USA) using a mobile phase consisting of water (A) and acetonitrile (B), each containing 0.1% formic acid. An injection volume of 5 µL was introduced, and a linear gradient elution from 5% to 100% B in 7 min, followed by isocratic at 100% B for 2 min, and decreased to 5% B at the final step for 2 min were applied.

Positive ionization mode was applied in this study. The optimized HESI-II parameters were set as follows: source voltage, 3.1 to 3.7 kV (pos); sheath gas flow rate (N_2_), 35 units; auxiliary gas flow rate, 10 units; sweep gas flow rate, 1.0; capillary temperature, 320 °C (pos); S–Lens RF Level, 55. The mass analyzer was calibrated using the Thermo Scientific EASY-IC ion source internal reference mass (fluoranthene). The mass spectrometer method was set to FullMS data-dependent MS2 (ddMS2) for a scan range between 100 and 1500 *m*/*z*.

The high-resolution analysis of the co-culture and the culture blank was carried out to enhance data quality and identify ions unique to the extract of *Micromonospora* sp. SH-82. Retention time stability was monitored by the periodic injection (every ten samples) of a quality control (QC) mixture, along with regular injections of QC standards to assess column performance over time. No retention time shifts were observed during the analyses, allowing us to maintain a tight RT shift range for data processing with MZmine. No quantitative analysis was performed.

### 2.7. Raw Data Processing and Ion Identity Molecular Network

The raw data from microbial extracts and isolated compounds were processed with the MZmine 3.6.0 software [38]. A detection threshold for MS1 and MS2 masses was set at 1 × 10^6^ and 1 × 10^0^, respectively. The feature tables were created with the ADAP chromatogram builder [39] and ADAP feature resolver. The alignment was performed with an *m*/*z* tolerance of 0.0070 Da and a retention time of 0.07 min. An *m*/*z* tolerance of 4.0 ppm was utilized for adduct identification. All parameters used for data processing are described in Table S6.

The Ion Identity Molecular Network [40] was generated on the GNPS platform [41,42], and the parameter sets are shown in Table S7. Visualization and graphical modifications were performed with Cytoscape 3.9.1 software [43].

Various bioinformatics and automated tools were used to annotate the features. The GNPS plateform [41], SIRIUS 5.8.2 software (Lehrstuhl für Bioinformatik, Jena, Germany) [44] and the tool for comparing data with in silico spectral databases of natural products (ISDB) [45] refined by timaR 2.7.2 software [46] and the LOTUS database [47] were used. Only results consistent with the molecular network structure, NMR identifications, scientific literature and identifications from the various bioinformatics tools are summarized in Table S5.

### 2.8. Biological Activities

Biological activity assays were conducted on microbial extract from *Micromonospora* sp. SH-82 and on isolated compounds **1**, **2**, **3**, and **5**. The tests focused on antiplasmodial and cytotoxic activity. Detailed protocols for these activity tests can be found in reference [18].

The antiplasmodial activity was conducted by using in vitro cultures of the chloroquine-sensitive 3D7 strain of *Plasmodium falciparum* (BEI Resources, Manassas, VA, USA) following the methodology of Trager and Jensen [48]. The assays were performed in three independent experiments (*n* = 3), each conducted in duplicate. Artemisinin was used as the positive control, while DMSO served as the vehicle and negative control, representing 100% parasite growth. Untreated red blood cells were also included as a blank. The measurements consisted of determining the concentration required to inhibit 50% of parasite growth (IC_50_), expressed in µg/mL for the crude extract and in µM for the isolated compounds. IC_50_ values were calculated via simple linear regression.

The cytotoxic activity was performed on human colorectal HCT-116 and mammary MDA-MB-231 carcinoma cells. Human HCT-116 colorectal carcinoma cells were grown in Gibco McCoy’s 5A supplemented with 10% fetal calf serum and 1% glutamine. MDA-MB231 breast carcinomas were grown in RPMI 1640 supplemented with 10% fetal calf serum and 1% glutamine. Human hTERT-RPE1 cells were cultured in DMEM/F12 medium containing 10% fetal calf serum and 1% glutamine, enabling their impact on a healthy cell line. Cell lines were maintained at 37 °C in a humidified atmosphere containing 5% CO_2_. Cell viability was determined via a luminescent assay according to the manufacturer’s instructions (Promega, Madison, WI, USA). Briefly, the cells were seeded in 96-well plates (2.5 × 10^3^ cells/well) containing 90 μL of growth medium. After 24 h of culture, the cells were treated with the tested compounds at 10 and 1 μg/mL (extracts) or at 10 and 1 μM (pure compounds) final concentrations. Control cells were treated with the vehicle (DMSO), and no effect was observed from DMSO diluted at 1/1000. After 72 h of incubation, 100 μL of CellTiter Glo Reagent was added for 15 min before recording luminescence with a spectrophotometric plate reader PolarStar Omega (BMG LabTech, Ortenberg, Germany). The percent viability index was evaluated by two independent assays in triplicate.

## 3. Results and Discussion

### 3.1. Strain Characterization, Identification, and Genomic Analysis

**Figure 1:**
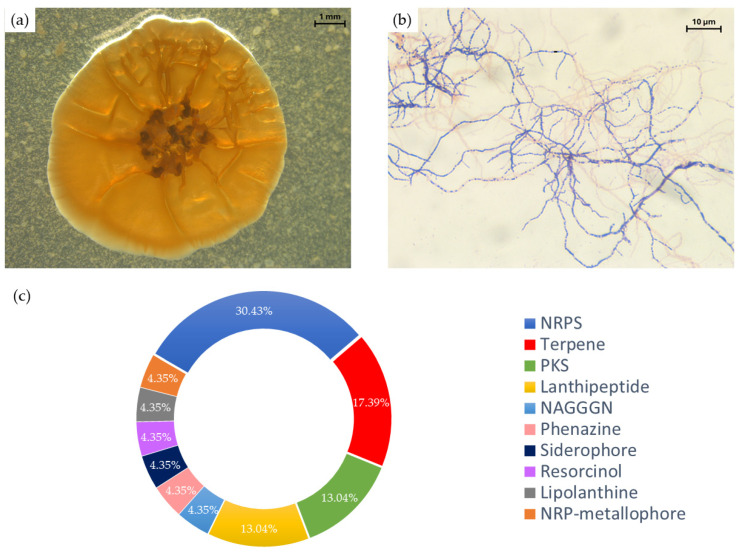
(**a**) Macroscopic (×10) and (**b**) microscopic (×1000) observation of a culture of *Micromonospora* sp. SH-82 on A1 medium for 21 days. (**c**) Distribution of biosynthetic gene clusters (BGCs) in *Micromonospora* sp. SH-82 strain.

Concise characterization, identification, and genomic analysis of *Micromonospora* sp. SH-82 were conducted. The aim was to provide a more detailed description of this strain to improve our understanding of its metabolic potential. *Micromonospora* sp. SH-82 was cultivated under improved conditions, on A1BFe+C solid medium for 21 days at 28 °C [19]. Macroscopic observation revealed a circular shape of the colony, with a bulging relief, undulating contour, rough surface, dry consistency, and a characteristic orange color (Figure 1a). Over time, darker colonies emerged (Figure S1), with an average diameter of 8 mm.

Gram staining (Figure 1b) categorizes this strain as Gram-positive with extensive branched and fragmented mycelial hyphae of 0.5–0.7 µm in diameter. Single spores are 0.2–0.3 µm in diameter and are produced on the hyphae or detached.

A *Micromonospora* sp. SH-82 culture was sent to the company Genoscreen for whole-genome sequencing. The results presented here come from a detailed genome analysis of this strain [49]. Figure S2 depicts a phylogenetic tree based on 16S rRNA, which highlights a distinct clustering of *Micromonospora* sp. SH-82 among 63 other genetically characterized *Micromonospora* strains used to build this figure, suggesting that it corresponds to an unidentified strain. A recent study [9] showed that using 16S rRNA alone is not sufficient for an accurate identification. Pairwise genome-to-genome comparisons were, therefore, performed to identify this strain, by calculating average nucleotide identity (ANI) values [50]. A matrix of results was generated using 64 *Micromonospora* genomes (Figure S3). The ANI values for our strain did not exceed 85%, and the dDDH values ranged from 21% to 30%, both well below the thresholds (95% for ANI and 70% for dDDH) used to define bacterial species [51]. Thus, the results suggest that *Micromonospora* sp. SH-82 constitutes a new species. However, no formal taxonomic description or valid Latin name has been proposed so far.

The genomic analysis indicates a genome size of 6.25 Mb with a high GC content of 71.6%, typical of the *Micromonospora* genus [5,9]. Further, 23 biosynthetic gene clusters (BGCs) were highlighted for this strain, corresponding to a slightly higher number compared to the 50 *Micromonospora* strains, where only 15 had more than 21 BGCs [7], highlighting its uniqueness. Figure 1c displays the distribution of the 23 BGCs in ten metabolic pathway classes, as predicted by antiSMASH, highlighting the prevalence of genes coding for non-ribosomal protein synthases (NRPSs), terpenes, polyketide synthases (PKSs), and lanthipeptide-type compounds. This extensive and diverse repertoire of specialized genes aligns with the remarkable chemical diversity recently revealed within the genus *Micromonospora* [5,7,9]. Among the BGCs identified in the genome, two displayed over 80% similarity to clusters previously associated with the biosynthesis of bioactive metabolites [49]. The BGC with the highest similarity (90%) corresponds to a non-ribosomal peptide synthetase (NRPS) cluster involved in the biosynthesis of coelibactin, a predicted zincophore in *Streptomyces coelicolor* known to regulate antibiotic production [52]. Another cluster showed 84% similarity to the loseolamycin BGC, which encodes an alkylresorcinol-type polyketide produced through the heterologous expression of a type III PKS gene from *Micromonospora endolithica*, and reported for its antibacterial and herbicidal activities [53]. In contrast, 17 BGCs showed less than 50% similarity, suggesting the presence of novel or poorly characterized biosynthetic pathways [49]. These findings highlight the biosynthetic richness of *Micromonospora* sp. SH-82 and emphasize the relevance of implementing targeted strategies, particularly molecular approaches, to activate silent genes, especially those involved in PKS and NRPS pathways, and to unlock their potential for the discovery of novel secondary metabolites.

### 3.2. Chemical Investigation

*Micromonospora* sp. SH-82, a new marine-derived species, has already demonstrated promising metabolic potential [18]. For its chemical investigation, a specific methodological strategy was adopted. The compounds were selected based on their abundance in the extract and the successful outcome of their isolation. They were subsequently identified by NMR and ESI^+^-HRMS. Subsequently, metabolite profiling of the crude extract by LC-HRMS/MS was carried out, and molecular networking analysis was conducted to explore the chemical diversity and identify potential new molecules related to compounds characterized by NMR.

#### 3.2.1. Purification and Structural Elucidation

The isolation process was conducted on a delipidated ethyl acetate extract obtained from the culture of *Micromonospora* sp. SH-82 under optimized conditions on A1 solid medium with amberlite XAD-16 resin for 21 days. The delipidation was carried out in order to concentrate microbial secondary metabolites and improve the separation and analysis of compounds. The purification methodology was established as in Figure S4. Initially, the crude extract was analyzed using UHPLC-PDA-ELSD-MS on a reversed-phase C18 analytical column (50 × 2.1 mm, 1.7 µm) to evaluate and quantify chemical diversity for metabolite isolation work. The separation was optimized by HPLC-ELSD on a column with a similar stationary phase (250 × 4.6 mm, 5 µm). This resulted in the determination of an optimized elution gradient of 10–50% acetonitrile (ACN) over a duration of 60 min. These optimized analytical conditions were then transferred to a semi-preparative column with the same stationary phase (250 × 19 mm, 5 µm) using a gradient transfer method [36]. To maintain the separation resolution at the preparative scale, the crude extract was injected via ‘dry load’ on the column [37]. The purity of the isolated metabolites was then controlled by UHPLC-PDA-ELSD-MS.

**Figure 2:**
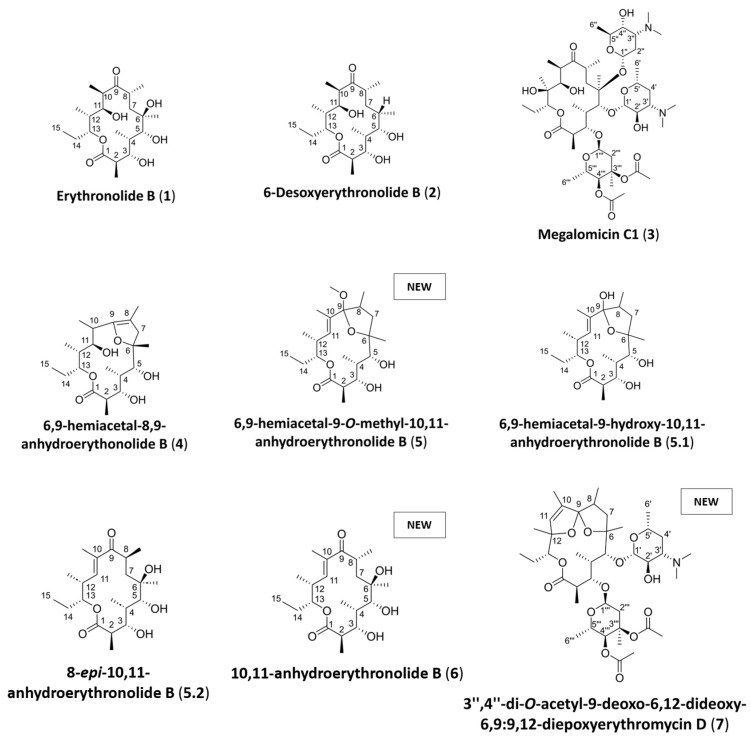
Structures of compounds **1–7** isolated from *Micromonospora* sp. SH-82.

Using this approach, seven pure molecules (Figure 2) were isolated in one step, among which three are potentially new (**5**, **6** and **7**). The identified molecules belong to families of bioactive metabolites such as erythronolides, erythromycins, and megalomicins [54,55,56,57,58]. The study of their biosynthetic pathways highlights the connection between these metabolites [59,60].

The known compounds (**1**–**4**) were identified exclusively through a comparison of NMR and ESI^+^-HRMS data with the literature. Compound **1** was identified as erythronolide B [61], **2** as 6-desoxyerythronolide B [62,63,64], **3** as megalomicin C1 [65] and **4** as 6,9-hemiacetal-8,9-anhydroerythonolide B [63]. NMR identification, spectra, and ESI^+^-HRMS data are provided in the Supplementary Materials (Tables S1–S4 and Figures S5–S30). Compounds **1**, **2**, and **4** belong to the family of erythronolides, molecules known to be intermediates in the biosynthesis of erythromycins [66]. The first isolated intermediate was 6-deoxyerythronolide B (**2**), discovered from a strain of *Streptomyces erythreus* [66]. Megalomicin C1 (**3**) is final product of erythromycin synthesis and was first isolated from a strain of *Micromonospora megalomicea* [67]. Like erythromycins, the megalomicin family exhibits various biological activities, including antibiotic properties [58,60].

**Figure 3:**
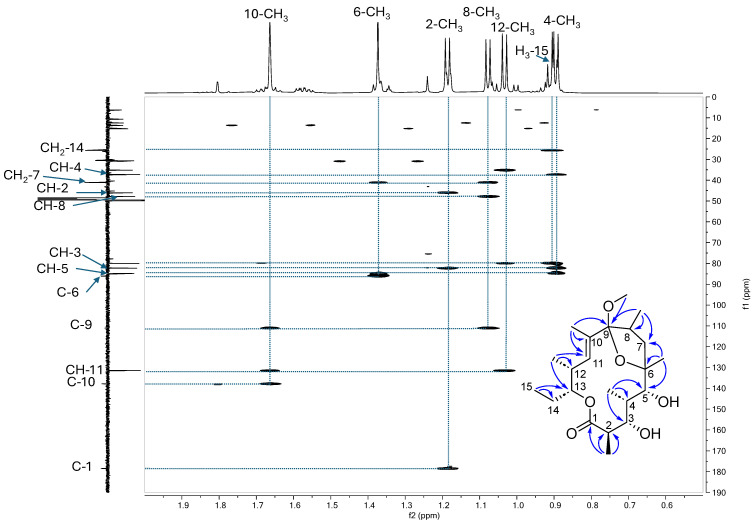
HMBC spectrum in the methyl region showing the main HMBC correlations useful for the identification of compound **5**. Structure of compound **5** and HMBC correlations (blue arrows).

The ^1^H and ^13^C NMR data of compound **5** (Table 1) showed that, when compared to erythronolide B (**1**), the carbonyl C-9 was missing, a double bond was present with an ethylenic proton at δ_H_ 5.86 (δ_C_ 131.5) and we observed a substituted ethylenic carbon at δ_C_ 137.8, a di-oxygenated quaternary carbon at δ_C_ 111.1, one oxygenated proton less (three instead of four), a methoxy group at δ_H_ 3.11 (δ_C_ 50.0) and one of the methyl doublets, which was replaced by a methyl triplet at δ_H_ 1.66 (δ_C_ 13.7) with a small coupling constant (*J* = 1.3 Hz). The HMBC correlations from the methyl doublet in C-2 (δ_H_ 1.19) to the ester carbonyl C-1 (δ_C_ 178.4), the methine CH-2 (δ_C_ 46.1) and the oxygenated methine CH-3 (δ_C_ 82.3); from the methyl doublet in C-4 (δ_H_ 0.89) to CH-3, the methine CH-4 (δ_C_ 37.3) and the oxygenated methine CH-5 (δ_C_ 84.8); the methyl singlet in C-6 to H-5, the oxygenated quaternary carbon C-6 (δ_C_ 86.1) and the methylene CH_2_-7 (δ_C_ 41.2) indicated the same C-1 to C-7 sequence as that of erythronolide B (Figure 3). The correlations from the methyl doublet in C-8 to CH_2_-7; the methine CH-8 (δ_C_ 47.8) and the di-oxygenated quaternary carbon C-9; and from the methyl in C-10 (δ_H_ 1.66) to C-9, and the ethylenic carbons C-10 (δ_C_ 137.8) and CH-11 (δ_C_ 131.5) indicated that the double bond was positioned in C-10-C-11 and that the ketone in C-9 in erythronolide B was an acetal (Figure 3). The correlation for the methoxy group to C-9 placed the methoxyl in this position. The rest of the molecule is arranged like erythronolide B, as shown by the HMBC correlations from the methyl doublet in C-12 (δ_H_ 1.03) to CH-11, the methine CH-12 (δ_C_ 35.2) and the oxygenated methine CH-13 (δ_C_ 80.0) and from the methyl triplet (δ_H_ 0.90) to the methylene CH_2_-14 (δ_C_ 25.7) and CH-13 (Figure 3). A hemiacetal from C-6 to C-9 was deduced from the ^13^C chemical shift of C-6, which was at δ_C_ 75.6 in erythronolide B and 86.1 in **5**. The presence of a methoxy group indicates that it cannot be ruled out that this compound is formed during the analysis process. The molecule’s stereochemistry has yet to be determined. The structure of **5** was confirmed by HRMS data, showing an ion at *m*/*z* 399.2728 in positive mode corresponding to the [M+H]^+^ adduct and to the molecular formula C_22_H_39_O_6_. NMR spectra and ESI^+^-HRMS data are provided in Figures S31–S37. Compound **5** was, thus, identified as 6,9-hemiacetal-9-*O*-methyl-10,11-anhydroerythronolide B.

**Table 1:** ^1^H and ^13^C NMR chemical shifts of compounds **5**, erythronolide B (**1**) and **5.1** in CD_3_OD.

	5		Erythronolide B (1)		5.1	
No	δ_H_ (Multiplicity, *J*)	δ_C_	δ_H_ (Multiplicity, *J*)	δ_C_	δ_H_ (Multiplicity, *J*)	δ_C_
1	-	178.4	-	177.6	-	178.7
2	2.58 (dq, 10.4, 6.9 Hz)	46.1	2.69 (dq, 10.5, 6.7 Hz)	45.1	2.58 (dq, 10.4, 6.9 Hz)	46.1
2-CH_3_	1.19 (d, 6.9 Hz)	15.3	1.19 (d, 6.7 Hz)	15.5	1.19 (d, 6.9 Hz)	13.2
3	3.74 (d, 10.4 Hz)	82.3	3.58 (dd, 10.5, 1.5 Hz)	80.3	3.75 (d, 10.4 Hz)	82.3
4	2.33 (q, 7.0 Hz)	37.3	2.16 (overlapped)	37.3	2.30 (q, 7.3 Hz)	37.3
4-CH_3_	0.89 (d, 7.0 Hz)	6.3	1.01 (d, 7.2 Hz)	8.1	0.90 (d, 7.3 Hz)	6.4
5	3.59 (s)	84.8	3.52 (d, 2.9 Hz)	82.6	3.60 (s)	84.7
6	-	86.1	-	75.8	-	86.2
6-CH_3_	1.37 (s)	31.0	1.30 (s)	26.4	1.37 (s)	30.9
7	1.37 (dd, 13.1, 12.0 Hz) 2.44 (dd, 13.1, 9.0 Hz)	41.2	1.98 (dd, 14.7, 9.3 Hz) 1.39 (dd, 14.7, 4.2 Hz)	38.8	1.37 (dd, 13.2, 12.0 Hz) 2.43 (dd, 13.2, 9.0 Hz)	41.1
8	2.06 (ddq, 12.0, 9.0, 6.7 Hz)	47.8	2.72 (dqd, 9.3, 7.0, 4.2 Hz)	45.5	2.06 (ddq, 12.0, 9.0, 6.8 Hz)	47.8
8-CH_3_	1.08 (d, 6.7 Hz)	15.2	1.14 (d, 7.0 Hz)	18.3	1.07 (d, 6.8 Hz)	15.1
9	-	111.1	-	220.5	-	111.1
10	-	137.8	3.03 (qd, 6.9, 1.8 Hz)	41.3	-	137.8
10-CH_3_	1.66 (t, 1.3 Hz)	13.7	0.96 (d, 6.9 Hz)	9.7	1.66 (d, 1.2 Hz)	13.7
11	5.86 (dq, 6.3, 1.3 Hz)	131.5	3.95 (dd, 10.1, 1.8 Hz)	71.3	5.84 (dq, 6.4, 1.2 Hz)	131.5
12	2.62 (pt, 6.9, 6.3, 2.5, 1.3 Hz)	35.2	1.66 (dq, 10.1, 6.5 Hz)	41.5	2.63 (overlapped)	35.2
12-CH_3_	1.03 (d, 6.9 Hz)	12.5	0.95 (d, 6.5 Hz)	9.7	1.03 (d, 6.9 Hz)	12.5
13	5.10 (ddd, 9.1, 5.0, 2.5 Hz)	80.0	5.44 (dd, 9.7, 4.6 Hz)	76.1	5.09 (overlapped)	80.2
14	1.67 (ddq, 14.2, 9.1, 7.4 Hz) 1.57 (dqd, 14.2, 7.4, 5.0 Hz)	25.7	1.74 (ddq, 14.0, 9.6, 7.4 Hz) 1.51 (dqd, 14.0, 7.4, 4.6 Hz)	26.9	1.66 (overlapped) 1.58 (overlapped)	25.6
15	0.90 (t, 7.4 Hz)	10.7	0.89 (t, 7.4 Hz)	10.9	0.90 (t, 7.3 Hz)	10.6
OCH_3_	3.11 (s)	50.0				

Prior to conducting biological assays, it was necessary to ascertain the stability of compound **5**. To this end, new NMR and ESI^+^-HRMS analyses were recorded (Figures S38–S43). The new ESI^+^-HRMS data obtained showed a molecular ion at *m*/*z* 367.2474 in positive mode corresponding to [M-H_2_O+H]^+^ adduct and, therefore, to the molecular formula of C_21_H_36_O_6_ (Δ4.5 ppm). This result is consistent with a molecule containing one CH_2_ less than that obtained for **5** (C_22_H_38_O_6_). The new NMR data showed that the signal corresponding to the methoxy group is no longer present and that a mixture of two compounds is observed. The first one could be identified as the 9-hydroxy derivative of **5** and, thus, to 6,9-hemiacetal-9-hydroxy-10,11-anhydroerythronolide B (**5.1**) (Figure 2). The second one has a carbonyl in C-9 at δ_C_ 208.8 and, therefore, corresponds to the open form of the first one: 8-*epi*-10,11-anhydroerythronolide B (**5.2**) (Figure 2).

The ESI^+^-HRMS data recorded for compound **6** displayed a major ion at *m*/*z* 367.2476 in positive mode corresponding to the [M-H_2_O+H]^+^ adduct, with the calculated molecular formula of C_21_H_36_O_6_. The ^1^H and ^13^C NMR data of **6** (Table 2) showed close similarities to erythronolide B (**1**) and **5**. The sequence from C-1 to the ketone C-9 corresponds to that of erythronolide B and could be followed by HMBC. The presence of a 10,11 unsaturation was established by the HMBC correlation from the methyl in C-10 (δ_H_ 1.74) to the carbonyl C-9 (δ_C_ 208.3), and the ethylenic carbons C-10 (δ_C_ 136.2) and CH-11 (δ_C_ 144.6) and from the methyl doublet in C-12 (δ_H_ 1.07) to CH-11, the methine CH-12 (δ_C_ 37.7) and the oxygenated methine CH-13 (δ_C_ 77.9). The presence of a hydroxyl in C-6 was confirmed by the ^13^C chemical shift value of C-6 (δ_C_ 76.5), which was close to that in erythronolide B (δ_C_ 75.8). The ROESY correlation from the methyl in C-10 to H-12 indicated the (*E*) configuration of the double bond. The other asymmetric carbons were kept in the same configuration as erythronolide B since chemical shifts and coupling constant values were close. Compound **6** was, thus, identified as 10,11-anhydroerythronolide B. NMR and ESI^+^-HRMS data are provided in Figures S44–S50. This compound was not the same isomer as **5.2** derived from the degradation of **5** (Table 2). A comparison of the chemical shifts and coupling constant values of **6** and **5.2** indicated that **5.2** could be 8-*epi*-10,11-anhydroerythronolide B.

**Table 2:** ^1^H and ^13^C NMR chemical shifts of compound **6** and **5.2** in CD_3_OD.

	6		5.2	
No	δ_H_ (Multiplicity, *J*)	δ_C_	δ_H_ (Multiplicity, *J*)	δ_C_
1	-	177.9	-	177.5
2	2.75 (dq, 10.4, 6.7 Hz)	45.3	2.63 (dq, 11.0, 6.7 Hz)	45.2
2-CH_3_	1.27 (d, 6.7 Hz)	15.5	1.18 (d, 6.7 Hz)	13.2
3	3.94 (dd, 10.4, 1.1 Hz)	80.2	3.63 (d, 11.0 Hz)	79.9
4	1.87 (qdd, 7.0, 2.9, 1.1)	38.4	2.02 (overlapped)	37.4
4-CH_3_	0.99 (d, 7.0 Hz)	7.1	1.06 (d, 7.1 Hz)	7.9
5	3.86 (d, 2.9 Hz)	81.7	3.37 (d, 4.3 Hz)	82.1
6	-	76.5	-	75.6
6-CH_3_	1.22 (s)	27.1	1.24 (s)	28.8
7	1.36 (dd, 14.8, 12.3 Hz) 1.71 (dd, 14.8, 2.6 Hz)	44.3	1.63 (dd, 14.3, 5.9 Hz) 1.84 (dd, 14.3, 8.2 Hz)	43.1
8	3.29 (overlapped)	37.5	3.49 (dqd, 8.2, 6.7, 5.9 Hz)	35.2
8-CH_3_	1.18 (d, 6.4 Hz)	16.4	1.00 (d, 6.7 Hz)	19.9
9	-	208.3	-	208.8
10	-	136.2	-	138.0
10-CH_3_	1.74 (d, 1.4 Hz)	11.7	1.80 (d, 1.2 Hz)	12.2
11	6.53 (dq, 8.9, 1.4 Hz)	144.6	6.40 (dq, 8.3, 1.2 Hz)	145.4
12	2.84 (dqd, 8.9, 7.0, 1.8 Hz)	37.7	2.88 (dqd, 8.3, 6.7, 2.4 Hz)	36.4
12-CH_3_	1.07 (d, 7.0 Hz)	12.3	1.18 (d, 6.7 Hz)	13.2
13	5.08 (ddd, 9.7, 4.3, 1.8 Hz)	77.9	5.07 (overlapped)	78.1
14	1.75 (dqd, 14.7, 9.7, 7.4 Hz) 1.61 (dqd, 14.7, 7.4, 4.3 Hz)	26.1	1.68 (m)	26.2
15	0.92 (t, 7.4 Hz)	10.7	0.92 (t, 7.4 Hz)	10.6
1	-	177.9	-	177.5

**Figure 4:**
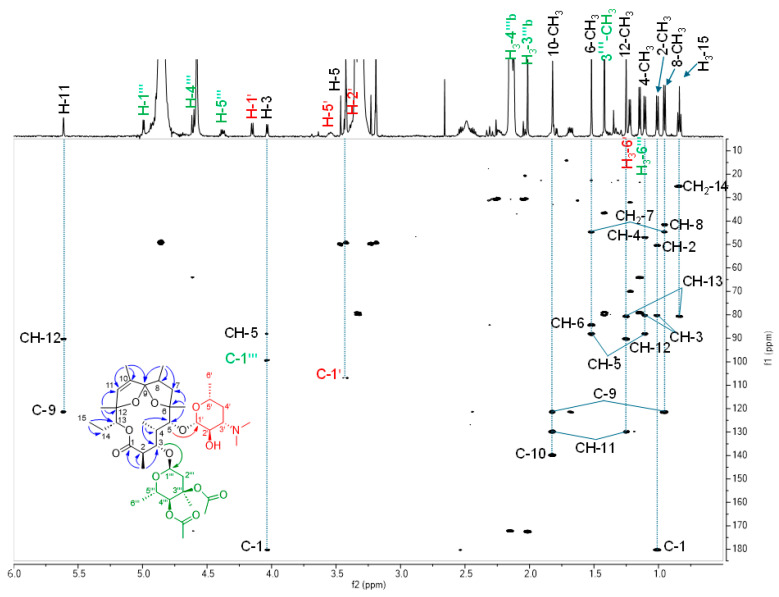
HMBC spectrum showing the main HMBC correlations useful for the identification of **7**. Structure of compound **7** and HMBC correlations (blue arrows). Other arrows represent HMBC correlations between the macrolide core and D-desosamine (in red) and L-mycarose (in green).

The ESI^+^-HRMS data of compound **7** showed a major ion at *m*/*z* 768.4528 in positive mode corresponding to the [M+H]^+^ adduct. The data show that the compound is similar to erythromycin and leads to a molecular formula of C_40_H_65_NO_13_ (Δ0.2 ppm). NMR identification and ESI^+^-HRMS data for **7** are provided in the Supplementary Materials (Figures S51–S56). The ^1^H and ^13^C NMR data for **7** are presented in Table 3. As for the other compounds, the HMBC correlations from the methyl groups were very useful to follow the connectivity of the aglycone. These correlations were as follows: from the methyl doublet in C-2 (δ_H_ 1.01) to the ester C-1 (δ_C_ 180.3), the methine CH-2 (δ_C_ 50.5) and the oxymethine CH-3 (δ_C_ 80.3); from the methyl doublet in C-4 (δ_H_ 1.11) to CH-3, the methine CH-4 (δ_C_ 46.9) and the oxymethine CH-5 (δ_C_ 88.3); from the methyl singlet in C-6 (δ_H_ 1.52) to CH-5, the oxygenated quaternary carbon C-6 (δ_C_ 84.4), and the methylene CH_2_-7 (δ_C_ 44.7); from the methyl doublet in C-8 (δ_H_ 0.95) to CH_2_-7, the methine CH-8 (δ_C_ 41.5) and the spiroketal C-9 (δ_C_ 121.2); from the methyl in C-10 (δ_H_ 1.82) to C-9, and the ethylenic carbons C-10 (δ_C_ 139.9) and CH-11 (δ_C_ 130.0); from the methyl singlet in C-12 (δ_H_ 1.25) to CH-11, the oxygenated quaternary carbon C-12 (δ_C_ 90.4) and the oxymethine CH-13 (δ_C_ 80.7); and from the methyl triplet CH_3_-15 (δ_H_ 0.84) to the methylene CH_2_-14 (δ_C_ 25.1) and CH-13 (Figure 4). The assignment of the sugar as D-desosamine and L-mycarose was achieved by comparison with that of megalomicin C1 (**3**). The HMBC correlation of H-5 to the anomeric carbon of D-desosamine at δ_C_ 106.9 and from H-3 to the anomeric carbon of L-mycarose at δ_C_ 99.3 allowed us to position these sugars. The chemical shifts of C-6 and C-12 (84.4 and 90.4, respectively) were in good agreement with the presence of 6,9:9,12-diepoxy. Compound **7** was, thus, identified as 3″,4″-di-*O*-acetyl-9-deoxo-6,12-dideoxy-6,9:9,12-diepoxyerythromycin D.

**Table 3:** ^1^H and ^13^C NMR chemical shifts of **7** and megalomicin C1 (**3**) in CD_3_OD. no: not observed.

	7		Megalomicin C1 (3)	
No	δ_H_ (Multiplicity, *J*)	δ_C_	δ_H_ (Multiplicity, *J*)	δ_C_
1	-	180.3	-	178.1
2	2.53 (qd, 7.4, 6.0 Hz)	50.5	2.94 (dq, 10.4, 7.2 Hz)	46.3
2-CH_3_	1.01 (d, 7.4 Hz)	16.0	1.20 (d, 7.2 Hz)	16.8
3	4.03 (d, 6.0 Hz)	80.3	4.55 (dd, 10.4, 1.5 Hz)	83.1
4	2.23 (dq, 9.6, 7.2 Hz)	46.9	2.04 (pd, 7.6, 6.2, 1.5 Hz)	39.1
4-CH_3_	1.11 (d, 7.2 Hz)	12.5	1.14 (d, 7.6 Hz)	10.5
5	3.43 (d, 9.6 Hz)	88.3	3.95 (d, 6.2 Hz)	83.6
6	-	84.4	-	82.3
6-CH_3_	1.52 (s)	31.3	1.58 (s)	19.8
7	2.31 (dd, 13.5, 12.8 Hz) 1.68 (dd, 12.8, 6.2 Hz)	44.7	2.05 (d, 15.1 Hz) 1.70 (d, 15.1 Hz)	39.8
8	2.44 (dqd, 13.5, 7.0, 6.2 Hz)	41.5	3.23 (q, 6.9 Hz)	38.9
8-CH_3_	0.95 (d, 7.0 Hz)	12.3	1.15 (d, 7.0 Hz)	12.5
9	-	121.2	-	no
10	-	139.9	2.55 (m)	46.9
10-CH_3_	1.82 (d, 1.6 Hz)	14.2	1.15 (d, 7.0 Hz)	19.2
11	5.61 (q, 1.6 Hz)	130.0	3.61 (d, 1.4 Hz)	70.5
12	-	90.4	-	76.0
12-CH_3_	1.25 (s)	23.4	1.19 (s)	17.6
13	4.89 (overlapped)	80.7	5.19 (dd, 11.3, 2.3 Hz)	78.6
14	1.69 (overlapped) 1.40 (overlapped)	25.1	1.90 (qd, 7.4, 2.3 Hz) 1.54 (dq, 11.3, 7.4 Hz)	22.2
15	0.84 (t, 7.3 Hz)	10.6	0.85 (t, 7.4 Hz)	10.9
D-desosamine				
1′	4.15 (d, 7.3 Hz)	106.9	4.55 (d, 7.1 Hz)	103.6
2′	3.39 (overlapped)	no	3.38 (d, 7.1 Hz)	71.5
3′	no	no	no	no
4′	1.84 (overlapped) 1.33 (overlapped)	32.0	1.91 (m) 1.41 (m)	31.7
5′	3.54 (m)	70.0	3.65 (p, 10.4, 6.3 Hz)	69.1
6′	1.22 (d, 6.1 Hz)	21.3	1.28 (d, 6.0 Hz)	22.0
3′N(CH_3_)_2_	2.49 (brs)	no	2.55/2.84	41.8
L-megasamine				
1″	-	-	4.98 (t, 6.8, 5.6 Hz)	91.0
2″	-	-	3.34 (overlapped) 1.83 (m)	30.6
3″	-	-	no	62.8
4″	-	-	3.82 (t, 1.8, 1.4 Hz)	66.3
5″	-	-	4.30 (qd, 7.4, 1.4 Hz)	75.1
6″	-	-	1.23 (d, 7.4 Hz)	15.2
3′N(CH_3_)_2_	-	-	2.55/2.84	41.8
L-mycarose				
1″′	4.99 (d, 4.6 Hz)	99.3	5.03 (d, 4.5 Hz)	99.0
2″′	3.34 (overlapped) 1.80 (dd, 15.3, 4.7 Hz)	36.5	3.34 (overlapped) 1.83 (m)	37.3
3″′	-	79.7	-	79.7
3″′-CH_3_	1.42 (s)	22.7	1.45 (s)	22.9
4″′	4.61 (d, 9.8 Hz)	79.1	4.60 (d, 9.6 Hz)	78.9
5″′	4.38 (dq, 9.8, 6.8 Hz)	64.2	4.25 (dq, 9.6, 6.1 Hz)	64.4
6″′	1.15 (d, 6.3 Hz)	17.6	1.20 (d, 6.1 Hz)	19.2
3″′a	-	172.4	-	172.7
3″′b	2.01 (s)	22.8	2.13 (s)	20.7
4″′a	-	172.2	-	172.0
4″′b	2.15 (s)	20.6	2.13 (s)	23.6

Even though there was only a small amount of microbial extract (about 250 mg), the isolation method allowed for seven pure molecules to be separated and identified using NMR and ESI^+^-HRMS analyses. The new NMR data, particularly the ^13^C spectra in CD_3_OD/CDCl_3_, provided valuable insights for known structures where such data were previously absent in the literature. These results highlight that the new *Micromonospora* SH-82 species is able to produce bioactive metabolites belonging to the biosynthetic pathway of megalomicin, as demonstrated in other species of the genera [60]. As parts of these metabolites are labile, some of the molecules described are probably artefacts, as discussed.

#### 3.2.2. Evaluation of the Chemical Diversity and Biosynthetic Potential of *Micromonospora* sp. SH-82 Through Ion Identity Molecular Networking

**Figure 5:**
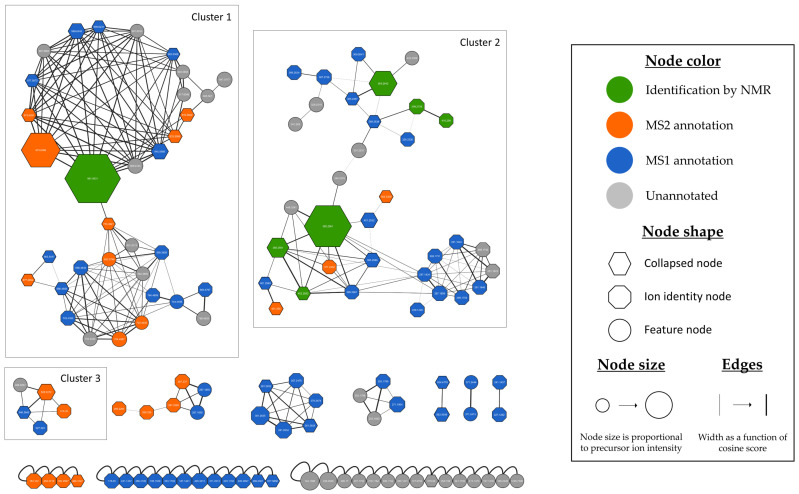
Ion Identity Molecular Network (IIMN) of the crude extract of *Micromonospora* sp. SH-82. Nodes are colored according to identification level: green for metabolites isolated and identified by NMR, orange for features annotated by MS2 indicating potential identification of microbial metabolites by computational tools, blue for features annotated by MS1 revealing identification of the adduct and associated molecular formula and grey for unannotated features.

The delipidated ethyl acetate extract and purified metabolites were analyzed via UHPLC-ESI^+^-HRMS/MS to further explore the chemical diversity and potential metabolic novelty of *Micromonospora* sp. SH-82 and to annotate potential additional minor metabolites. MS1 and MS2 data were processed through the MZmine 3.6.0 software [38] and the GNPS platform [41] to generate the Ion Identity Molecular Network (IIMN) [40] displayed in Figure 5. A combination of several bioinformatic pipelines was used for the annotation of detected features (*m*/*z* at a specific retention time). For each feature, MS2 spectra were compared to the GNPS databases providing experimental mass spectra and to the in silico fragmentation database of natural products (ISDB) [45] and LOTUS database [47] provided through the timaR pipeline [46]. In parallel, MS1 and MS2 spectra were processed through SIRIUS 5.8.2 software [44]. The results obtained from the different tools were compared, and only the most consistent annotations were retained. These were established either through propagation from metabolites isolated and identified by NMR, visible in the network, or by identifying the same annotation candidate structure using two distinct tools.

All these computational strategies combined with NMR structural elucidation of major compounds of the crude extract allowed us to propose different levels of annotation [68] for each feature of the network, as illustrated by the node color. The first level, in green, concerns metabolites isolated and identified by NMR. The second level, in orange, corresponds to metabolites annotated on the basis of their MS2 spectra and cluster consistency. The third level, in blue, represents the MS1 annotation of features, for which only molecular formulae were calculated based on the adduct type and the exact mass measured. Finally, the last, least precise level, in grey, corresponds to unannotated features. The IIMN enabled us to connect and collapse different ion species of the same molecule (mainly adducts) into a single node, called “collapsed node”. This network comprises 126 features (nodes), with 50% of them gathered into the two main clusters.

**Figure 6:**
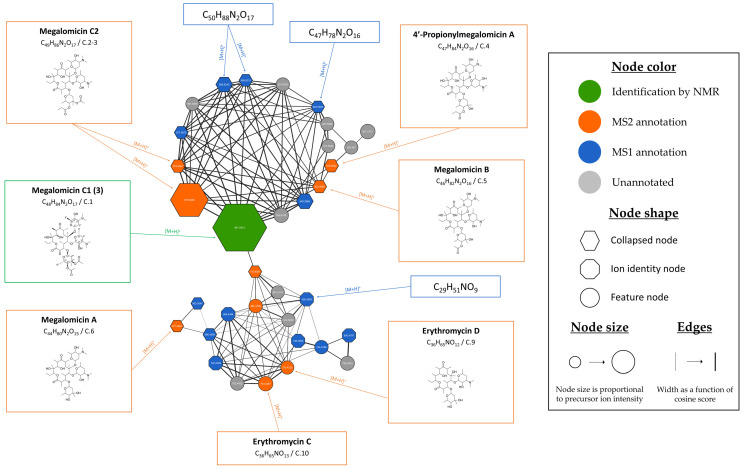
Focus over cluster 1 harboring megalomicin and erythromycin derivatives. Nodes are colored according to identification level: green for metabolites isolated and identified by NMR, orange for features annotated by MS2 indicating potential identification of microbial metabolites by bioinformatics tools, blue for features annotated by MS1 revealing identification of the adduct and associated molecular formula and grey for unannotated features.

Six of the seven isolated microbial metabolites isolated were found within the IIMN. Twenty-four nodes were annotated using bioinformatic tools as metabolites listed in natural product databases. Their detailed information, including their compound ID, their *m*/*z* ratio and given retention time, their adduct type, and their putative compound name and percentage of similarity to known compound in the databases, is provided in Table S5. Moreover, different EICs representing the annotated compounds are provided in Figure S57. For 60 nodes, the molecular formula has been calculated. Clusters 1 and 2 are the largest within the MN. Cluster 1 displayed in Figure 6 harbors 34 nodes related to megalomicins and erythromycins derivatives.

**Figure 7:**
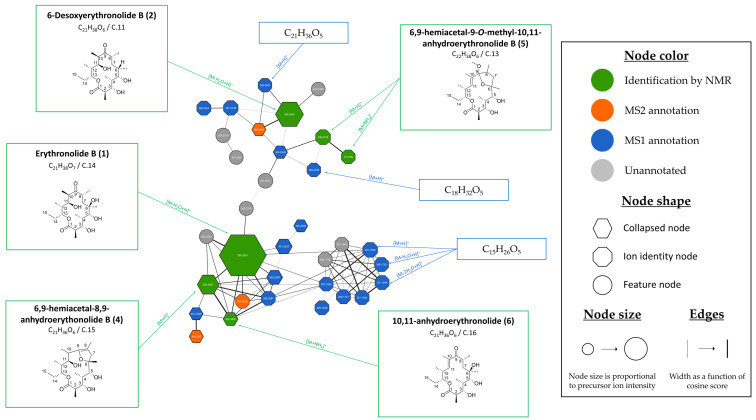
Focus over cluster 2 harboring erythronolide derivatives. Nodes are colored according to identification level: green for metabolites isolated and identified by NMR, orange for features annotated by MS2 indicating potential identification of microbial metabolites by bioinformatics tools, blue for features annotated by MS1 revealing identification of the adduct and associated molecular formula and grey for unannotated features.

The upper part of cluster 1 harbors one of the seven purified compounds, megalomicin C1 (**3**) (C.1, *m*/*z* 961.5931 [M+H]^+^, calculated for C_48_H_85_N_2_O_17_, 961.5848, Δ8.6 ppm). Cluster 1 contains six nodes (C.1–C.6) annotated as belonging to the megalomicins, such as megalomicin A (C.6, *m*/*z* 877.5640 [M+H]^+^, calcd for C_44_H_81_N_2_O_15_, 877.5637, Δ0.3 ppm) with a high similarity score (92.24%) provided by the SIRIUS pipeline. The lower part of the cluster gathers features annotated as erythromycin D (C.9, *m*/*z* 704.4587 [M+H]^+^, calcd for C_36_H_66_NO_12_, 704.4585, Δ0.3 ppm), erythromycin C (C.10, *m*/*z* 720.4535 [M+H]^+^, calcd for C_36_H_66_NO_13_, 720.4534, Δ0.1 ppm) and two other putative erythromycin derivatives (C7–C8) represented by orange nodes. The detection of these molecules is consistent with the study of the megalomicin biosynthesis pathway, which reveals the presence of these two erythromycins as precursors [59,60]. Further, 70% of the nodes remain unannotated (grey) or only by their molecular formula (blue), suggesting the possible existence of new megalomicin derivatives. The second major cluster comprises compounds belonging to the erythronolide family, encompassing 34 nodes, as illustrated in Figure 7.

Inspection of cluster 2 revealed the grouping of five of the seven purified compounds (**1**,**2** and **4**–**6**). Despite the large size of the cluster, one-third of the nodes are collapsed as erythronolides tend to generate several adducts, such as [M+H]^+^, [M-H_2_O+H]^+^, [M+NH_4_]^+^ and [M+Na]^+^ when ionized. This phenomenon is illustrated in Figure S58 for erythronolide B (**1**) (C.14, *m*/*z* 385.2561 [M-H_2_O+H]^+^, calcd for C_21_H_37_O_6_, 385.2590, Δ7.6 ppm). Due to the analytical conditions and their pronounced structural similarity, most of the megalomicins and the erythronolides tended to coelute, making the deconvolution step rather challenging, which complicated the analysis of the molecular network and the annotation of the features.

Erythronolides, erythromycins and megalomicins identified in this network, along with the metabolites annotated in cluster 3 (Figure S59), are all part of the megalomicin biosynthetic pathway [60]. The fact that these metabolites are linked to this pathway reinforces the relevance of the annotations, as their presence is explained by their role in this biosynthetic process. The identification of macrolides is consistent with the presence of genetic clusters responsible for their synthesis in this strain. The PKS-type BGC located in region 11 has a similarity rate of 94% to the one responsible for megalomicin synthesis from *Micromonospora megalomicea* (Figure S60) [60] Compounds **5**, **6**, and **7** exhibit a rare double bond at C10–C11 within this type of structure. This structural characteristic could be either genetically encoded [69] or the result of a spontaneous reaction [70].

Compounds **5.1**, **5.2**, and **7**, identified by NMR, could not be specifically localized within the IIMN of the crude extract. These compounds could be transformation byproducts, formed during the isolation process or sample storage. It is important to note that erythronolides, including compound **4** described in the literature as a degradation product, are sensitive to changes in acidic environments [63,70]. These modifications can occur during culture through acidification of the medium by microorganisms or following the initial extraction involving the use of formic acid in the isolation process.

The large number of nodes not annotated (grey) or only by their molecular formula (blue) indicates the presence of several potential novel metabolites. The challenges associated with isolating, conserving, and identifying these molecules highlight the necessity to produce larger quantities of extracts and improve analysis and purification techniques in order to isolate a greater number of novel microbial metabolites. This work as well as previous studies [18,19] highlight the potential of *Micromonospora* sp. SH-82 for the production of bioactive metabolites. The isolation and characterization of some of these metabolites confirmed the presence of bioactive molecular families, further emphasizing the interest in this strain.

Additionally, the identification of biosynthetic gene clusters (BGCs) encoding molecules of interest, such as loseolamycin, but not detected in our extracts, suggests that some BGCs might be cryptic. Moreover, a significant number of PKS- and NRPS-type BGCs remain unassociated with known metabolites, highlighting the relevance of combining genomic, metabolomic, and molecular biology approaches to activate these genes [49]. For instance, transferring the mich BGC from *Micromonospora* sp. SCSIO 07395 to *Streptomyces albus* enabled the production of five new benzoxazole alkaloids that were not detected in the original strain [71]. This integrated strategy not only enhances our ability to uncover novel bioactive compounds but also contributes to improving database annotations by linking BGCs to their metabolic products. Such insights reinforce the interest in *Micromonospora* sp. SH-82 for future investigations in biotechnology and drug discovery.

### 3.3. Biological Activity

The ethyl acetate extract of *Micromonospora* sp. SH-82 and compounds **1**, **2**, **3**, and **5** were tested for their cytotoxic activity in three cell lines, MDA-MB-231, HCT-116 and RPE1. The first two cell lines were utilized as models to discover new drugs against colon and breast cancer, respectively. RPE1 was used to assess the cytotoxicity of the samples on a non-cancerous cell line, a critical factor for their potential future use as medications. The raw extract was tested at 10 and 1 µg/mL, while the pure molecules were tested at 10 and 1 µM. The results in Table 4 display the percentages of cell viability.

The tested samples did not exhibit any cytotoxic activity on the MDA-MB-231 and HCT-116 cell lines. The difference in the cytotoxic activity of the crude extract compared to previous results could be due to the delipidation performed in this study [19]. Biological activity may reside in the apolar fraction or result from the synergy between polar and apolar compounds. The results on the non-cancerous RPE1 cell line are crucial in the context of potential medicinal use. They show that our samples demonstrate no toxicity towards healthy cells, displaying viability percentages close to 100%, thus paving the way for exploring these metabolites for other biological activities.

Despite the lack of activity of our molecules on cancerous cell lines, the literature has highlighted that various erythromycins, when used alone or in combination, have demonstrated chemopreventive and inhibitory activities on cancer cells [72,73]. These data suggest the possibility of isolating molecules from this family for testing on various molecular targets to discover novel anticancer agents.

The microbial extracts and megalomicin C1 (**3**) were also tested for their antiplasmodial activity against a strain of *Plasmodium falciparum* 3D7. The concentrations inhibiting 50% of parasite growth (IC_50_) were measured for the crude extract (µg/mL) and for the pure molecule (µM) (Table 5).

**Figure 8:**
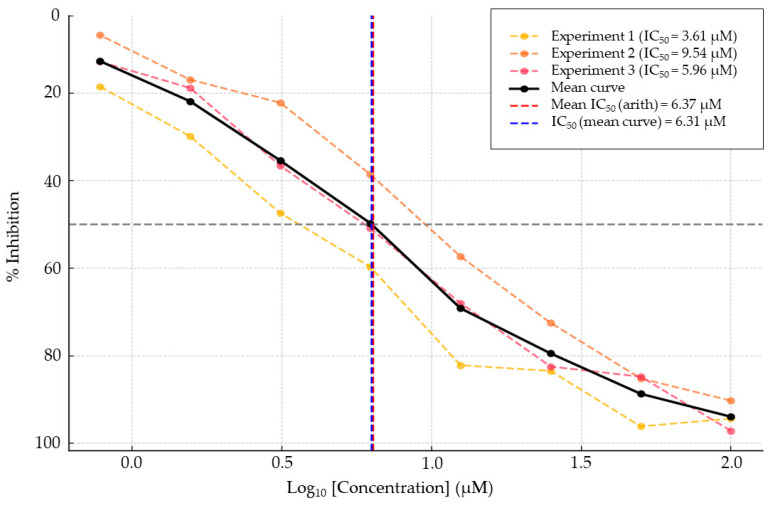
Dose–response curves from three independent experiments evaluating the activity of megalomicin C1 (compound **3**). Each curve represents the percentage of inhibition as a function of the log_10_ concentration. Vertical dashed lines indicate the arithmetic mean IC_50_ (red) and the IC_50_ obtained from the average dose–response curve (blue).

The measured IC_50_ for the crude extract (16.29 ± 1.22 µg/mL) is remarkable, suggesting the possible presence of bioactive molecules. The activity of the crude extract might be related, therefore, to the presence of megalomicins, already known for their antiparasitic activities against various parasites [56]. Earlier, a study also compared the antiparasitic activities of erythromycins and megalomicins against a strain of *Trypanosoma cruzi* [56], highlighting only the activity of the latter. Dose–response curves from three independent experiments evaluating the activity of megalomicin C1 (compound **3**) are presented in Figure 8.

Results from an isolated metabolite showed promising activity for megalomicin C1 (**3**) with an IC_50_ of 6,37 ± 2.99 µM (6,12 ± 2.87 µg/mL). This value was obtained from three independent experiments and corresponds to the arithmetic mean of the IC_50_ values. The chemical analysis of the crude extract revealed the presence of numerous microbial metabolites, with a significant amount of macrolides. Among them, megalomicins were identified, exhibiting interesting antibiotic and antiviral activities [10,56,58,60]. It would, therefore, be relevant to attempt the isolation of other known megalomicins, as well as those present in low abundance in the extract, in order to evaluate their antiplasmodial potential.

The chemical diversity observed in *Micromonospora* sp. SH-82 reflects a rich secondary metabolism. In addition to megalomicins, erythromycin was also annotated in the extract, a well-known antibiotic widely used to treat respiratory infections such as bronchitis [74]. The co-occurrence of both known and potentially novel macrolides highlights the versatile biosynthetic capacity of this species.

Another important aspect lies in the spontaneous formation of erythromycin or erythronolide-like derivatives. Several reports in the literature show that semi-synthetic analogues of known macrolides, such as clarithromycin and azithromycin, exhibit improved stability and an expanded spectrum of activity compared to their parent compound [74,75]. These examples underscore the potential of naturally modified or spontaneously transformed derivatives as promising sources of new pharmacologically relevant structures [76,77].

Moreover, several BGCs of the PKS and NRPS types remain unassigned to known metabolites, suggesting the presence of cryptic biosynthetic pathways. Integrated approaches combining genomics, metabolomics, and synthetic biology could allow for the activation of these silent BGCs and the discovery of novel chemical scaffolds [12,16].

Beyond their pharmaceutical potential, marine actinomycetes such as Micromonospora represent valuable resources for biotechnology. They are known producers of thermostable enzymes (e.g., cellulases, lipases), antifungal agents, natural pigments, and bioactive metabolites with agricultural or environmental applications [78,79]. Their adaptation to extreme marine environments also promotes the emergence of unique biosynthetic clusters. A broader comparison with other marine strains and the exploration of their metabolomes could help uncover new biological activities or potential industrial applications.

## 4. Conclusions

This study highlights the relevance of a multidisciplinary approach to explore a rare marine-derived actinobacterium, *Micromonospora* sp. SH-82, as a source of new bioactive natural products. The selected strain, isolated from the sponge *Scopalina hapalia* (ML-263), was characterized genomically as a new species (maximum ANI value of only 83%), with a high number of biosynthetic gene clusters (BGCs), totaling 23 identified, underscoring its significant potential for secondary metabolite production. Notably, polyketide synthase (PKS) types predominate, playing a crucial role in the biosynthesis of important compounds such as macrolides, and include several unannotated clusters potentially involved in the production of yet-unknown but bioactive metabolites.

This work led to the isolation and characterization of seven microbial metabolites belonging to the chemical families of erythronolides, erythromycins, and megalomicins. Among them, three potentially new structures were described, possessing atypical structural features such as double bonds or hemiacetal formation: 6,9-hemiacetal-9-*O*-methyl-10,11-anhydroerythronolide B (**5**), 10,11-anhydroerythronolide B (**6**), and 3″,4″-di-*O*-acetyl-9-deoxo-6,12-dideoxy-6,9:9,12-diepoxyerythromycin D (**7**). Molecular networking combined with NMR analysis enabled the annotation of 24 microbial metabolites. Genomic and literature data confirmed the presence of key intermediates such as erythronolides and erythromycin, which are involved in megalomicin biosynthesis. Their detection in the crude extract is, therefore, consistent with the predicted biosynthetic pathways. The presence of a significant number of unidentified metabolites, including megalomicins, suggests potential interest in the discovery of new structures. Megalomicin C1 (**3**) showed interesting antiplasmodial activity against *Plasmodium falciparum* 3D7) with an IC_50_ of 6.37 ± 2.99 µM. Moreover, none of the tested compounds showed cytotoxicity, reinforcing their pharmacological potential. In addition, the spontaneous formation of analogues through rearrangements or chemical modifications of known macrolides may lead to improved pharmacological profiles, such as enhanced stability or a broader spectrum of action, as observed with some semi-synthetic erythromycin derivatives.

Despite technical challenges related to metabolite yield and structural elucidation, the chemical diversity observed and the genomic richness of *Micromonospora* sp. SH-82 underline its value for drug discovery and biotechnological exploitation. Further efforts should focus on the activation of silent BGCs through approaches such as co-culture or heterologous expression, as well as through large-scale production to enable full characterization of low-abundance metabolites. Future biological evaluations should expand to a broader panel of pathogens (including resistant strains) and cancer models to fully assess the therapeutic potential of these compounds.

## Figures and Tables

**Table 4:** Cytotoxic activity of the crude extract and pure compounds (**1**–**3** and **5**) against three cell lines (MDA-MB-231, HCT-116 and RPE1) expressed as a percentage of cell viability.

	Percentage of Cell Viability ^a^					
	MDA-MB-231 Cell Line		HCT-116 Cell Line		RPE1 Cell Line	
Concentration Tested ^b^	10 µg/mL or 10 µM	1 µg/mL or 1 µM	10 µg/mL or 10 µM	1 µg/mL or 1 µM	10 µg/mL or 10 µM	1 µg/mL or 1 µM
*Micromonospora* sp. SH-82 extract	101 ± 1.22	101 ± 0.67	93.1 ± 1.82	98.3 ± 1.73	94.55 ± 1.89	98.34 ± 1.48
Erythronolide B (**1**)	100 ± 0.76	107 ± 0.86	104 ± 1.16	101 ± 0.5	97.28 ± 1.03	101.53 ± 1.58
6-deoxyerythronolide B (**2**)	101 ± 1.13	105 ± 1.56	103 ± 3.56	101 ± 1.62	95.28 ± 0.96	99.08 ± 1.40
Megalomicin C1 (**3**)	98.6 ± 1.38	102 ± 1.74	97.3 ± 2.22	100 ± 1.66	103.84 ± 1.94	104.19 ± 1.32
Erythronolide B derivative (**5**)	99 ± 3.28	100 ± 1.74	99.7 ± 0.59	99.1 ± 0.32	103.38 ± 1.77	103.19 ± 1.52

^a^ Percentage of cell viability was evaluated by two independent assays in triplicate. ^b^ Concentration tested in µg/mL for the extract and in µM for the pure molecules.

**Table 5:** Antiplasmodial activity of the crude extract and the pure compound megalomicin C1 (**3**) expressed by the median inhibitory concentrations (IC_50_) against *Plasmodium falciparum* 3D7.

	IC_50_ (µg/mL or µM) ^a,b^
*Micromonospora* sp. SH-82 extract	16.29 ± 1.22 µg/mL
Megalomicin C1 (**3**)	6.12 ± 2.87 µg/mL 6.37 ± 2.99 µM

^a^ Half-maximal inhibitory concentrations are the means ± standard deviations calculated from three independent assays against *P. falciparum* 3D7 strain. ^b^ Artemisinin was used as a positive control, IC_50_: 6.36 ng/mL.

## Data Availability

The original contributions presented in this study are included in the article. Further inquiries can be directed to the corresponding author.

## Supplementary Materials

The following supporting information can be downloaded at: https://www.mdpi.com/article/10.3390/microorganisms13092045/s1, Figure S1: Macroscopic observation of *Micromonospora* sp. SH-82 culture with amberlite XAD-16 on A1BFe+C solid medium at 7, 14, and 21 days. Figure S2: 16s rRNA based phylogenetic tree showing distinct clustering of *Micromonospora* sp. SH-82 among 62 *Micromonospora* strains. Figure S3: Illustration depicting ANI based on 64 genomes of *Micromonospora*. Figure S4: Purification method for *Micromonospora* sp. SH-82 extract, (a) chromatogram UHPLC-ELSD (C18 50 × 2.1 mm, 1.7 m; 0–100% ACN in 10 min), (b) chromatogram HPLC-ELSD optimized (C18 250 × 4.6 mm, 5 m; 10–50% ACN in 60 min), (c) chromatogram HPLC-ELSD semi-preparative “dry load” injection (C18 250 × 19 mm, 5 m; 10–50% ACN in 60 min). Table S1: 1H and 13C NMR chemical shifts of compound **1** in CD_3_OD and erythronolide B reported by Mulzer et al. (1991) [61] in DMSO-*d*6. Figure S5: 1H NMR spectrum of erythronolide B (1) in CD_3_OD at 600 MHz. Figure S6: COSY NMR spectrum of erythronolide B (1) in CD_3_OD. Figure S7: Edited HSQC NMR spectrum of erythronolide B (1) in CD_3_OD. Figure S8: HMBC NMR spectrum of erythronolide B (1) in CD_3_OD. Figure S9: ROESY NMR spectrum of erythronolide B (1) in CD_3_OD. Figure S10: ESI^+^-HRMS data in positive mode of erythronolide B (1). Table S2: 1H and 13C NMR chemical shifts of compound **2** in CDCl3 and 13C NMR chemical shifts of 6-desoxyerythronolide B reported by Nourse, J. G. and J. D. Roberts (1975) [63] in CDCl3. Figure S11: 1H NMR spectrum of 6-deoxyerythronolide B (2) in CDCl3 at 600 MHz. Figure S12: COSY NMR spectrum of 6-deoxyerythronolide B (2) in CDCl3. Figure S13: Edited HSQC NMR spectrum of 6-deoxyerythronolide B (2) in CDCl3. Figure S14: HMBC NMR spectrum of 6-deoxyerythronolide B (2) in CDCl3. Figure S15: ROESY NMR spectrum of 6-deoxyerythronolide B (2) in CDCl3. Figure S16: ESI^+^-HRMS data in positive mode of 6-deoxyerythronolide B (2). Table S3: 1H and 13C NMR chemical shifts of compound **3** in CD_3_OD and 13C NMR chemical shifts of megalomicin C1 reported by Bartner, P. et al. (1979) [65] in CDCl3. n.o.: not observed. Figure S17: 1H NMR spectrum of megalomicin C1 (3) in CD_3_OD at 600 MHz. Figure S18: COSY NMR spectrum of megalomicin C1 (3) in CD_3_OD. Figure S19: 13C-DEPTQ NMR spectrum of megalomicin C1 (3) in CD_3_OD at 151 MHz. Figure S20: Edited HSQC NMR spectrum of megalomicin C1 (3) in CD_3_OD. Figure S21: HMBC NMR spectrum of megalomicin C1 (3) in CD_3_OD. Figure S22: ROESY NMR spectrum of megalomicin C1 (compound **3**) in CD_3_OD. Figure S23: ESI^+^-HRMS data in positive mode of megalomicin C1 (3). Table S4: 1H and 13C NMR chemical shifts of compound **4** in CD_3_OD and 13C NMR chemical shifts of 6,9-hemiacetal-8,9-anhydroerythonolide B reported by Nourse J.G. and Roberts (1975) [63] J.D. in 4:1 (*v*/*v*) mixtures of CDCI3 and CH2CI2. Figure S24: 1H NMR spectrum of 6,9-hemiacetal-8,9-anhydroerythonolide B (4) in CD_3_OD at 600 MHz. Figure S25: COSY NMR spectrum of 6,9-hemiacetal-8,9-anhydroerythonolide B (4) in CD_3_OD. Figure S26:13C-DEPTQ NMR spectrum of 6,9-hemiacetal-8,9-anhydroerythonolide B (4) in CD_3_OD at 151 MHz. Figure S27: Edited HSQC NMR spectrum of 6,9-hemiacetal-8,9-anhydroerythonolide B (4) in CD_3_OD. Figure S28: HMBC NMR spectrum of 6,9-hemiacetal-8,9-anhydroerythonolide B (4) in CD_3_OD. Figure S29: ROESY NMR spectrum of 6,9-hemiacetal-8,9-anhydroerythonolide B (4) in CD_3_OD. Figure S30: ESI^+^-HRMS data in positive mode of 6,9-hemiacetal-8,9-anhydroerythonolide B (4). Figure S31: 1H NMR spectrum of 6,9-hemiacetal-9-*O*-methyl-10,11-anhydroerythronolide B (5) in CD_3_OD at 600 MHz. Figure S32: COSY NMR spectrum of 6,9-hemiacetal-9-*O*-methyl-10,11-anhydroerythronolide B (5) in CD_3_OD. Figure S33: 13C-DEPTQ NMR spectrum of 6,9-hemiacetal-9-*O*-methyl-10,11-anhydroerythronolide B (compound **5**) in CD_3_OD at 151 MHz. Figure S34: Edited HSQC NMR spectrum of 6,9-hemiacetal-9-*O*-methyl-10,11-anhydroerythronolide B (5) in CD_3_OD. Figure S35: HMBC NMR spectrum of 6,9-hemiacetal-9-*O*-methyl-10,11-anhydroerythronolide B (5) in CD_3_OD. Figure S36: ROESY NMR spectrum of 6,9-hemiacetal-9-*O*-methyl-10,11-anhydroerythronolide B (5) in CD_3_OD. Figure S37: ESI^+^-HRMS data in positive mode of 6,9-hemiacetal-9-*O*-methyl-10,11-anhydroerythronolide B (5) in microbial raw extract. Figure S38: 1H NMR spectrum of 6,9-hemiacetal-9-hydroxy-10,11-anhydroerythronolide B (5.1) and 8-*epi*-10,11-anhydroerythronolide B (5.2) in CD_3_OD at 600 MHz. Figure S39: COSY NMR spectrum of 6,9-hemiacetal-9-hydroxy-10,11-anhydroerythronolide B (5.1) and 8-*epi*-10,11-anhydroerythronolide B (5.2) in CD_3_OD at 600 MHz. Figure S40: Edited HSQC NMR spectrum of 6,9-hemiacetal-9-hydroxy-10,11-anhydroerythronolide B (5.1) and 8-*epi*-10,11-anhydroerythronolide B (5.2) in CD_3_OD at 600 MHz. Figure S41: HMBC NMR spectrum of 6,9-hemiacetal-9-hydroxy-10,11-anhydroerythronolide B (5.1) and 8-*epi*-10,11-anhydroerythronolide B (5.2) in CD_3_OD at 600 MHz. Figure S42: ROESY NMR spectrum of 6,9-hemiacetal-9-hydroxy-10,11-anhydroerythronolide B (5.1) and 8-*epi*-10,11-anhydroerythronolide B (5.2) in CD_3_OD at 600 MHz. Figure S43: ESI^+^-HRMS data in positive mode of 6,9-hemiacetal-9-hydroxy-10,11-anhydroerythronolide B (5.1) and 8-*epi*-10,11-anhydroerythronolide B (5.2) one year later. Figure S44: 1H NMR spectrum of 10,11-anhydroerythronolide B (6) in CD_3_OD at 600 MHz. Figure S45: COSY NMR spectrum of 10,11-anhydroerythronolide B (6) in CD_3_OD. Figure S46: 13C-DEPTQ NMR spectrum of 10,11-anhydroerythronolide B (6) in CD_3_OD at 151 MHz. Figure S47: Edited HSQC NMR spectrum of 10,11-anhydroerythronolide B (6) in CD_3_OD. Figure S48: HMBC NMR spectrum of 10,11-anhydroerythronolide B (6) in CD_3_OD. Figure S49: ROESY NMR spectrum of 10,11-anhydroerythronolide B (6) in CD_3_OD. Figure S50: ESI^+^-HRMS data in positive mode of 10,11-anhydroerythronolide B (6). Figure S51: 1H NMR spectrum of 3″,4″-di-*O*-acetyl- 9-deoxo-6,12-dideoxy-6,9:9,12-diepoxyerythromycin D (7) in CD_3_OD at 600 MHz. Figure S52: COSY NMR spectrum of 3″,4″-di-*O*-acetyl- 9-deoxo-6,12-dideoxy-6,9:9,12-diepoxyerythromycin D (7) in CD_3_OD. Figure S53: Edited HSQC NMR spectrum of 3″,4″-di-*O*-acetyl- 9-deoxo-6,12-dideoxy-6,9:9,12-diepoxyerythromycin D (7) in CD_3_OD. Figure S54: HMBC NMR spectrum of 3″,4″-di-*O*-acetyl- 9-deoxo-6,12-dideoxy-6,9:9,12-diepoxyerythromycin D (7) in CD_3_OD. Figure S55: ROESY NMR spectrum of 3″,4″-di-*O*-acetyl- 9-deoxo-6,12-dideoxy-6,9:9,12-diepoxyerythromycin D (7) in CD_3_OD. Figure S56: ESI^+^-HRMS data in positive mode of 3″,4″-di-*O*-acetyl- 9-deoxo-6,12-dideoxy-6,9:9,12-diepoxyerythromycin D (7). Table S5: Summary table of annotations from the Ion Identity Molecular Network of *Micromonospora* sp. SH-82. Figure S57: Different EICs representing the annotated compounds. (a) EIC 1000–700 *m*/*z*, RT: 1.3–2.4 min; (b) EIC 750–500 *m*/*z*, RT: 1.9–3.3 min; (c) EIC 450–350 *m*/*z*, RT: 2.4–3.7 min; (d) EIC 300–100 *m*/*z*, RT: 4.0–5.7 min. Figure S58: ESI^+^-HRMS data in positive mode of erythronolide B (1) (*m*/*z* 385,2561 [M-H_2_O+H]^+^, C_21_H_38_O_7_) with the various adducts identified. Figure S59: Zoom-in on other clusters with MS2 annotation. Nodes are colored according to identification level: green for metabolites isolated and identified by NMR, orange for features annotated by MS2 indicating potential identification by bioinformatics tools, blue for features annotated by MS1 revealing identification of the adduct and associated molecular formula and grey for unannotated features. Figure S60: Similarity between the BGC from *Micromonospora* sp. SH-82 genome analysis (top) and the BGC0000092 from *Micromonospora megalomicea* responsible for megalomicin biosynthesis (bottom). Table S6: Batch mode use for data processing with MzMine 3 software. Table S7: Parameters used to create IIMNs on the GNPS platform.
