# Supplementary material for: New Marine Actinobacteria Strain, Micromonospora sp. SH-82: Characterization, Specialized Metabolites and Biological Activities
Source: Microorganisms. 2025 Sep 2;13(9):2045. doi: 10.3390/microorganisms13092045 (PMC12471853; doi:10.3390/microorganisms13092045)
Supplement: Supplementary file 1 [file microorganisms-13-02045-s001.zip › microorganisms-3674116-supplementary.pdf]

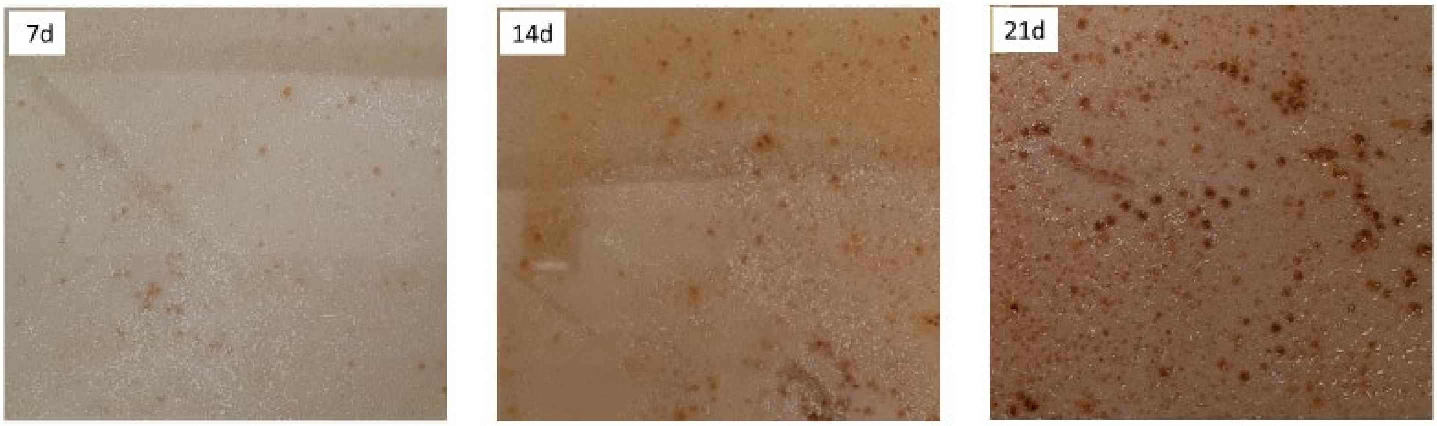

Figure S1: Macroscopic observation of *Micromonospora* sp. SH-82 culture with amberlite XAD-16 on A1BFe+C solid medium at 7, 14, and 21 days.

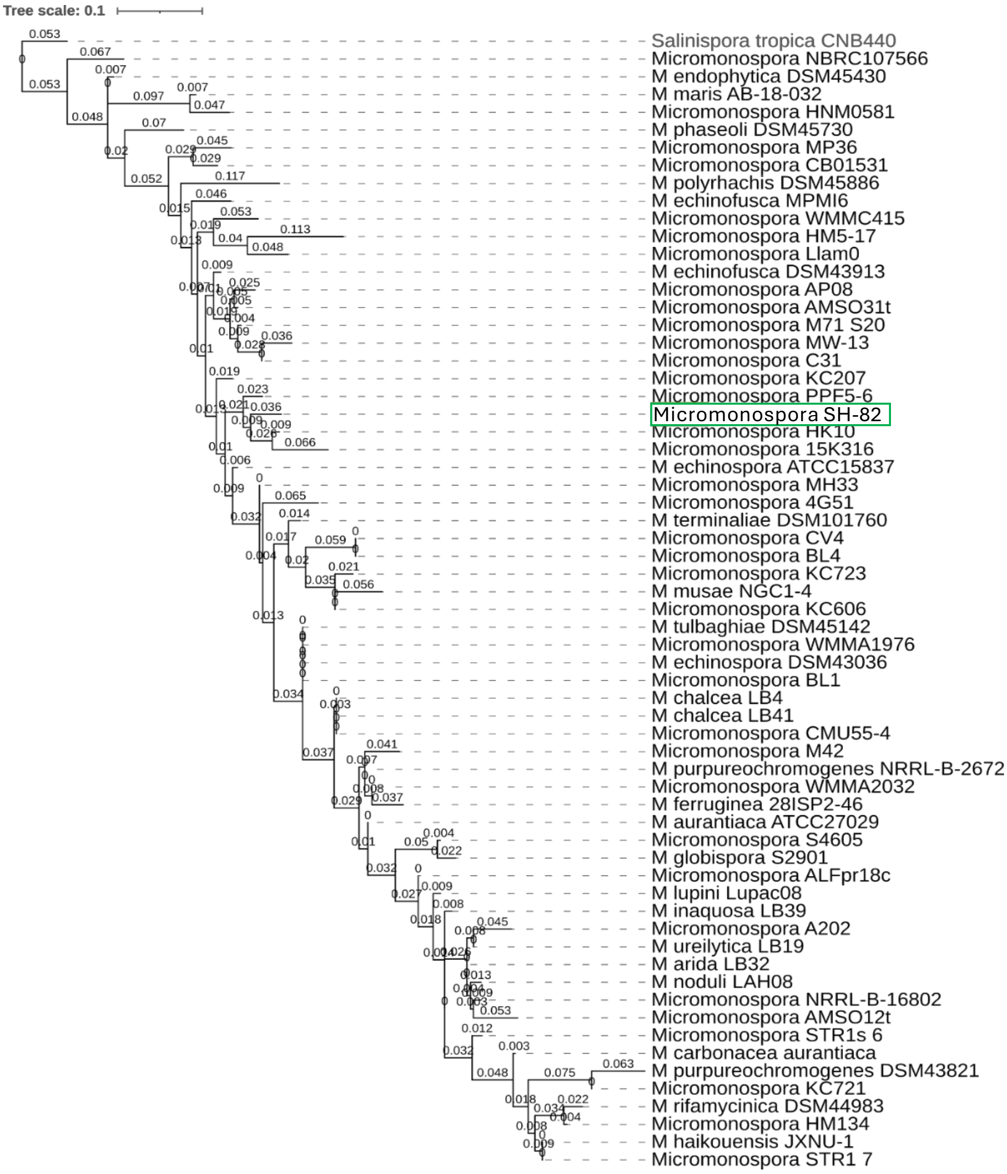

Figure S2: 16s rRNA based phylogenetic tree showing distinct clustering of *Micromonospora* sp. SH-82 among 62 *Micromonospora* strains.

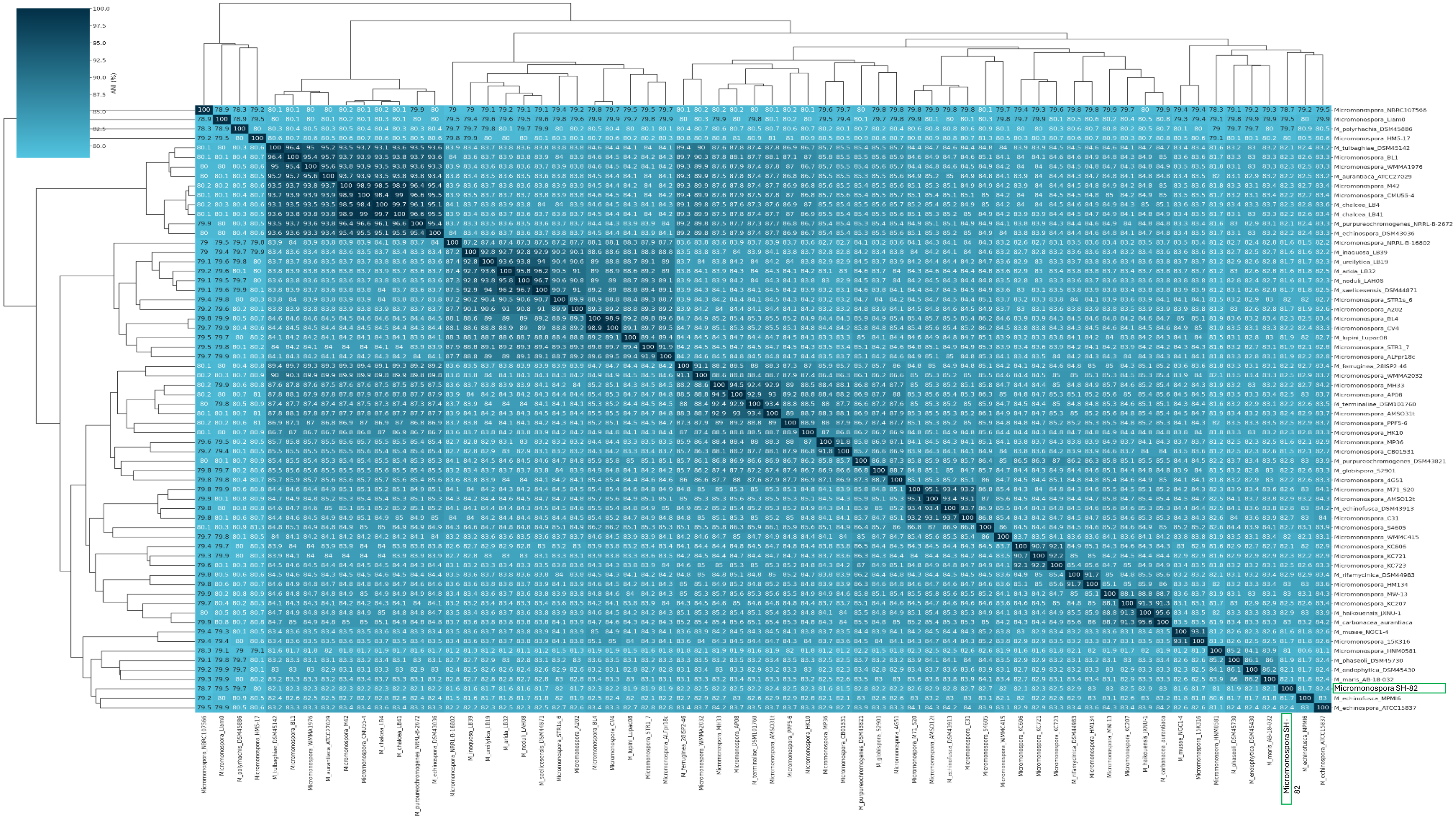

Figure S3: Illustration depicting ANI based on 64 genomes of *Micromonospora* strains

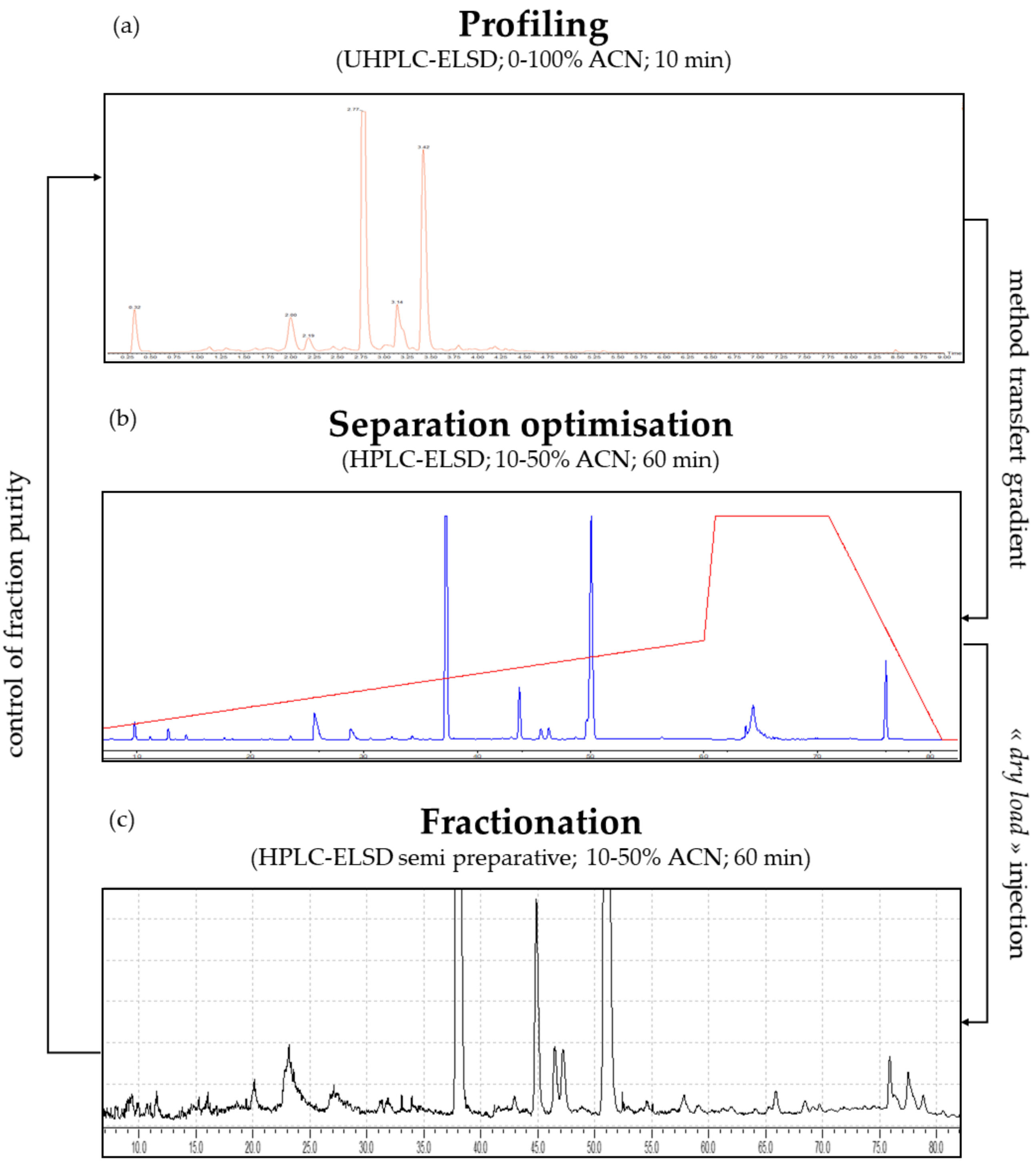

Figure S4: Purification method for *Micromonospora sp.* SH-82 extract, (**a**) chromatogram UHPLC-ELSD (C18 50 x 2.1 mm, 1.7 µm; 0-100% ACN in 10 min), (**b**) chromatogram HPLC-ELSD optimised (C18 250 x 4.6 mm, 5 µm; 10-50% ACN in 60 min), (**c**) chromatogram HPLC-ELSD semi-preparative "dry load" injection (C18 250 x 19 mm, 5 µm; 10-50% ACN in 60 min).

Table S1: ^{1}H and ^{13}C NMR chemical shifts of compound **1** in CD_3OD and erythronolide B reported by Mulzer *et al*. (1991) in DMSO-d_6.

	Compound **1** in CD_3OD		Erythronolide B [31] in DMSO-d_6	
No	δ_H (Multiplicity, *J*)	δ_C	δ_H (Multiplicity, *J*)	δ_C
1	-	177.6	-	174.9
2	2.69 (dq, 10.5,6.7Hz)	45.1	2.50 (m)	43.3
2-CH_3	1.19 (d, 6.7 Hz)	15.5	1.09 (d, 7.0 Hz)	15.0
3	3.58 (dd, 10.5,1.5 Hz)	80.3	3.51 (dd, 10.0,5.2 Hz)	77.5
4	2.16 (overlapped)	37.3	2.01 (m)	35.7
4-CH_3	1.01 (d, 7.2 Hz)	8.1	0.88 (d, 7.3 Hz)	7.6
5	3.52 (d, 2.9 Hz)	82.6	3.36-3.44	79.9
6	-	75.8		74.1
6-CH_3	1.30 (s)	26.4	1.18 (s)	26.3
7	1.98 (dd, 14.7,9.3 Hz)<br>1.39 (dd, 14.7,4.2 Hz)	38.8	1.85 (dd, 15.0,7.0 Hz)<br>1.19 (m)	37.7
8	2.72 (dqd, 9.3,7.0,4.2 Hz)	45.5	2.66 (m)	42.0
8-CH_3	1.14 (d, 7.0 Hz)	18.3	1.04 (d, 6.9 Hz)	17.3
9	-	220.5		216.3
10	3.03 (qd, 6.9,1.8 Hz)	41.3	2.84 (m)	40.5
10-CH_3	0.96 (d, 6.9 Hz)	9.7	0.85 (d, 7.5 Hz)	8.2/9.0
11	3.95 (dd, 10.1,1.8 Hz)	71.3	3.85 (dd, 10.5,4.5 Hz)	69.5
12	1.66 (dq, 10.1,6.5 Hz)	41.5	1.36-1.71	39.8
12-CH_3	0.95 (d, 6.5 Hz)	9.7	0.85 (d, 7.5 Hz)	8.2/9.0
13	5.44 (dd, 9.7,4.6 Hz)	76.1	5.34 (dq, 9.5,5.0 Hz)	73.6
14	1.74 (ddq, 14.0,9.6,7.4 Hz)<br>1.51 (dqd, 14.0,7.4,4.6 Hz)	26.9	1.36-1.71	25.4
15	0.89 (t, 7.4 Hz)	10.9	0.80 (t, 7.6 Hz)	10.2

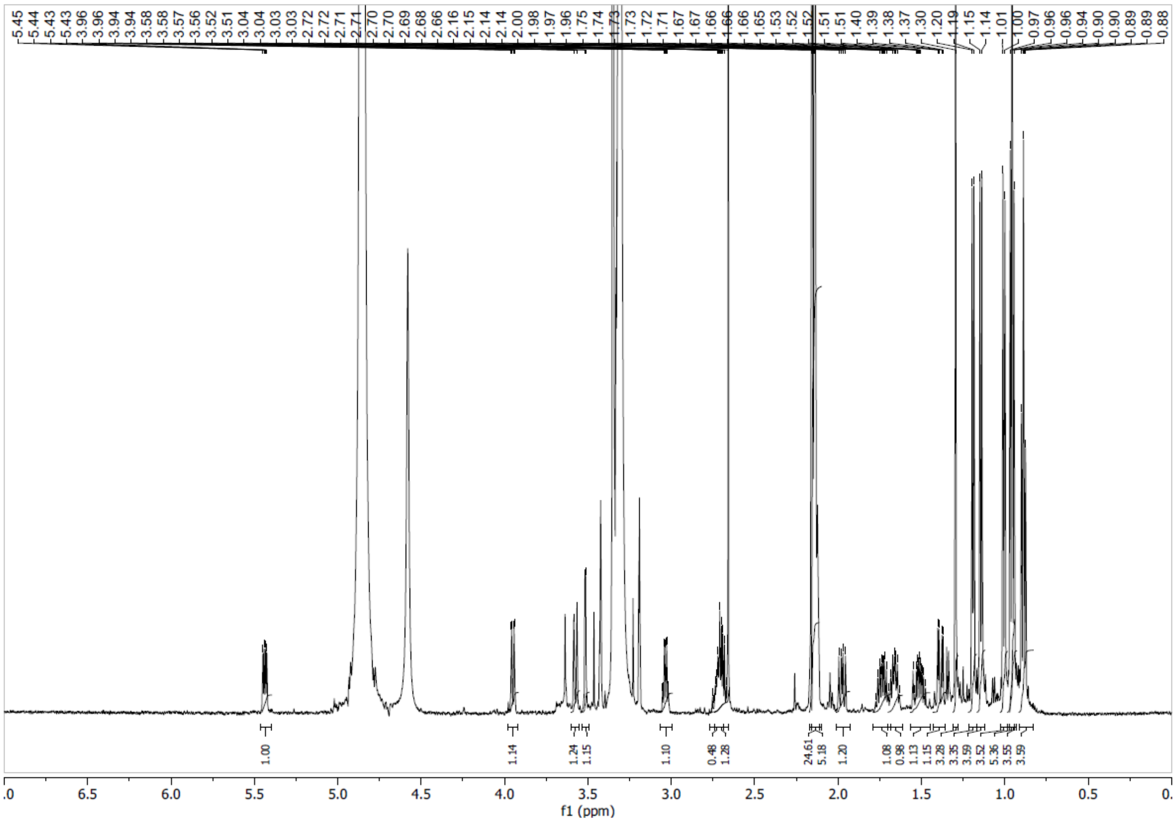

Figure S5: ^{1}H NMR spectrum of erythronolide B (**1**) in CD_3OD at 600 MHz.

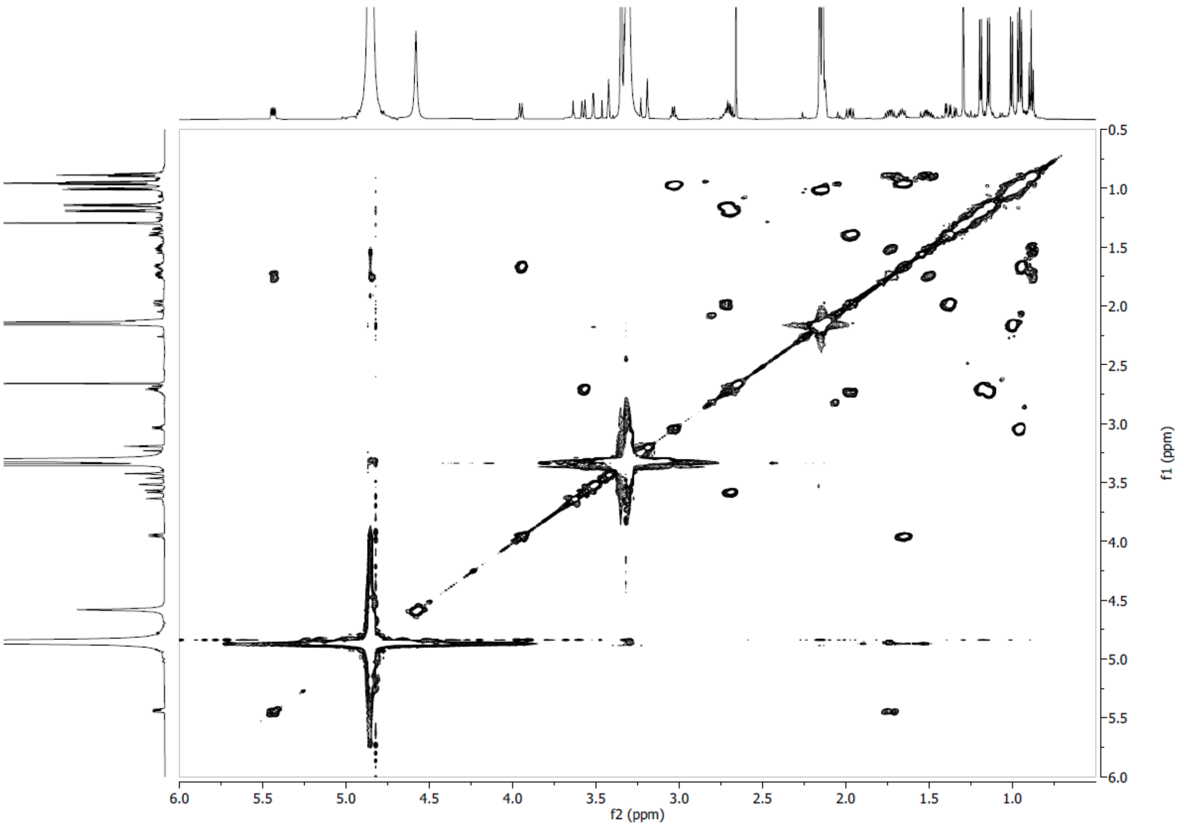

Figure S6: COSY NMR spectrum of erythronolide B (**1**) in CD_3OD.

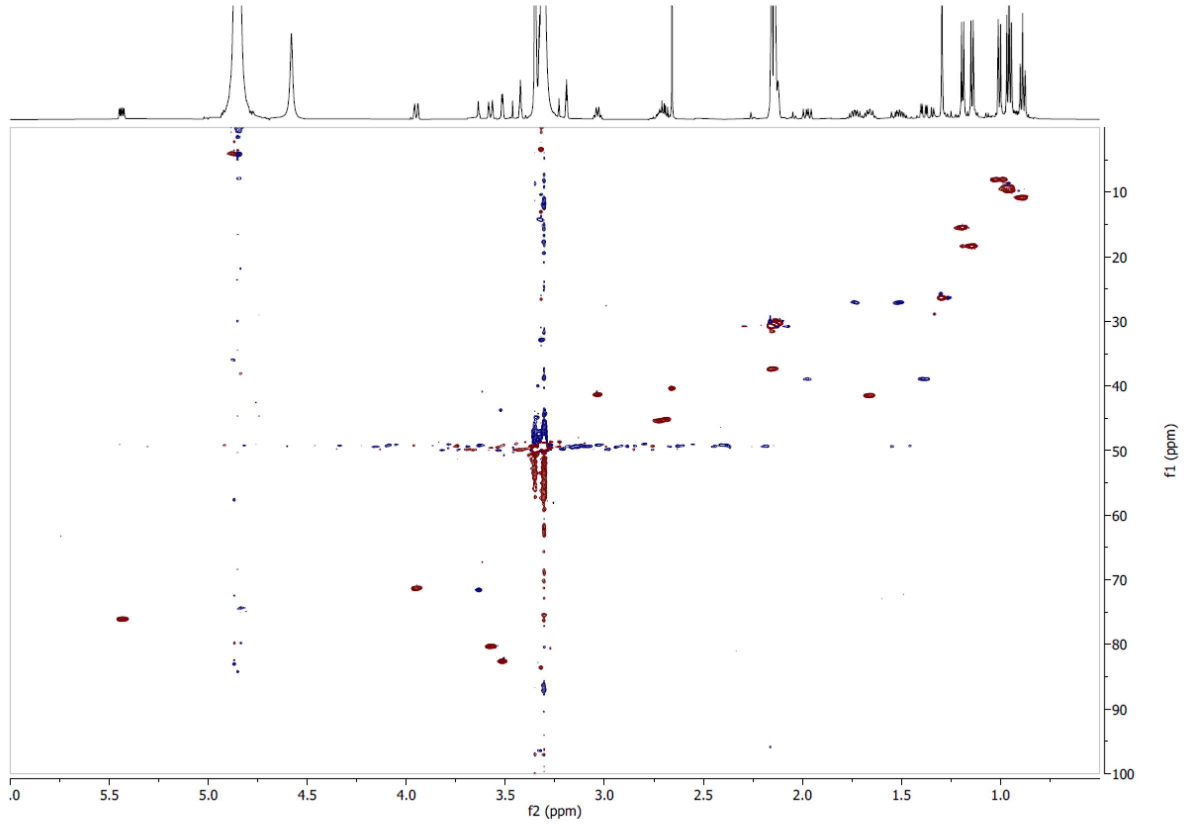

Figure S7: Edited HSQC NMR spectrum of erythronolide B (**1**) in CD_3OD.

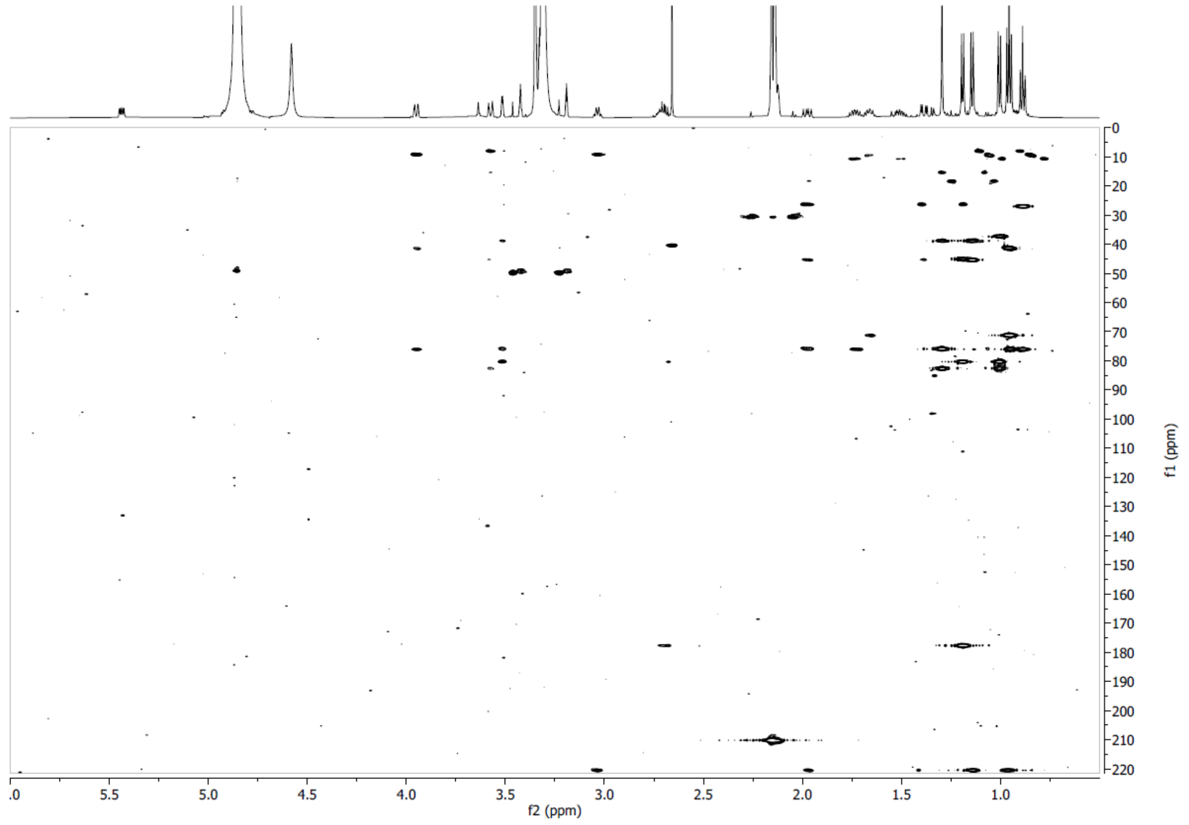

Figure S8: HMBC NMR spectrum of erythronolide B (**1**) in CD_3OD.

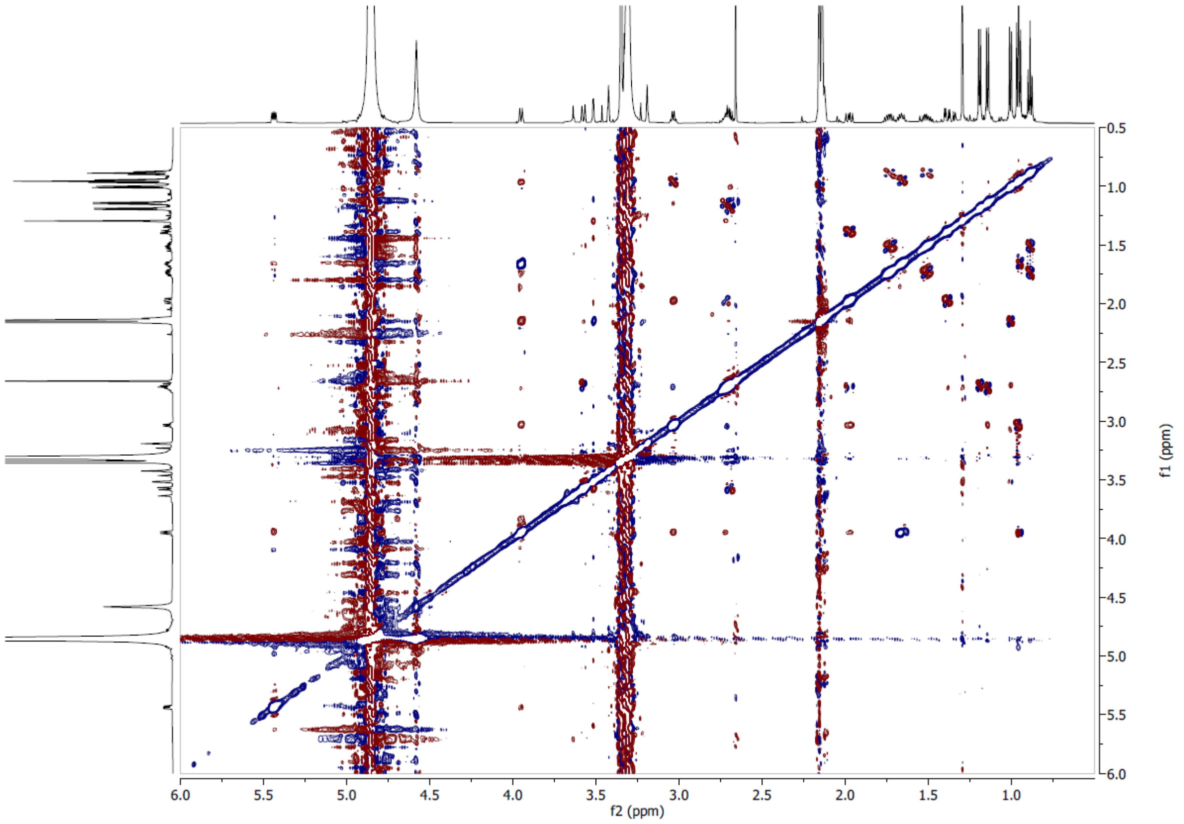

Figure S9: ROESY NMR spectrum of erythronolide B (**1**) in CD_3OD.

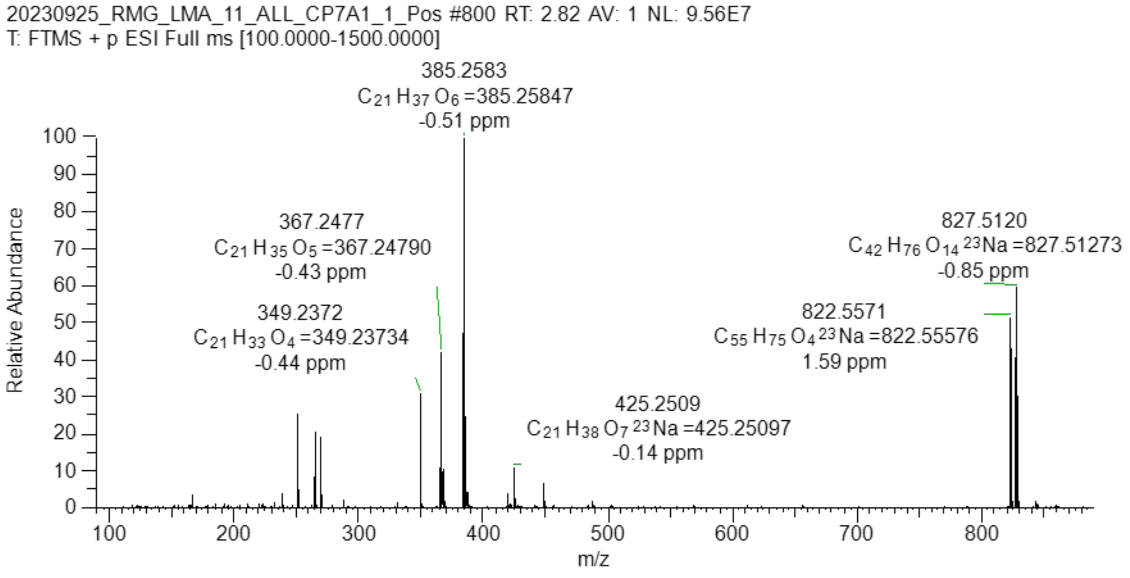

Figure S10: ESI+-HRMS data in positive mode of erythronolide B (**1**).

Table S2: ^{1}H and ^{13}C NMR chemical shifts of compound **2** in $CDCl_3$ and ^{13}C NMR chemical shifts of 6-desoxyerythronolide B reported by Nourse, J. G. and J. D. Roberts (1975) in $CDCl_3$.

	Compound **2** in $CDCl_3$		6-desoxyerythronolide B (Nourse, J. G. and J. D. Roberts, 1975) in $CDCl_3$
No	δ_H (Multiplicity, *J*)	δ_C	δ_C
1	-	178.6	178.7
2	2.79 (dq, 10.3,6.8 Hz)	44.2	43.7
2-CH_3	1.30 (d, 6.8 Hz)	15.0	14.8/13.4
3	3.92 (d, 10.3 Hz)	79.8	79.6
4	1.86 (overlapped)	37.6	37.8
4-CH_3	1.07 (d, 7.0 Hz)	7.1	7.0/6.3
5	4.01 (dd, 5.2,2.3 Hz)	76.7	76.5
6	2.03 (overlapped)	35.7	35.6
6-CH_3	1.05 (d, 6.3 Hz)	16.7	16.8
7	1.67 (overlapped)<br>1.25 (overlapped)	37.8	37.8
8	2.63 (dqd, 9.0,6.4,3.6 Hz)	39.4	40
8-CH_3	1.05 (d, 6.4 Hz)	13.4	6.3/7.0
9	-	213.6	214.9
10	2.76 (qd, 6.8,2.4 Hz)	43.6	44.2
10-CH_3	1.02 (d, 6.8 Hz)	6.4	13.4/14.8
11	3.68 (dd, 10.2,2.4 Hz)	71.0	71.2
12	1.73 (overlapped)	40.6	40.8
12-CH_3	0.89 (d, 7.0 Hz)	9.3	9.2
13	5.15 (ddd, 9.5,4.1,1.5 Hz)	76.5	76.5
14	1.52 (dqd, 14.4,7.4,4.1 Hz)<br>1.82 (overlapped)	25.9	26.6
15	0.93 (t, 7.4 Hz)	11.0	10.5

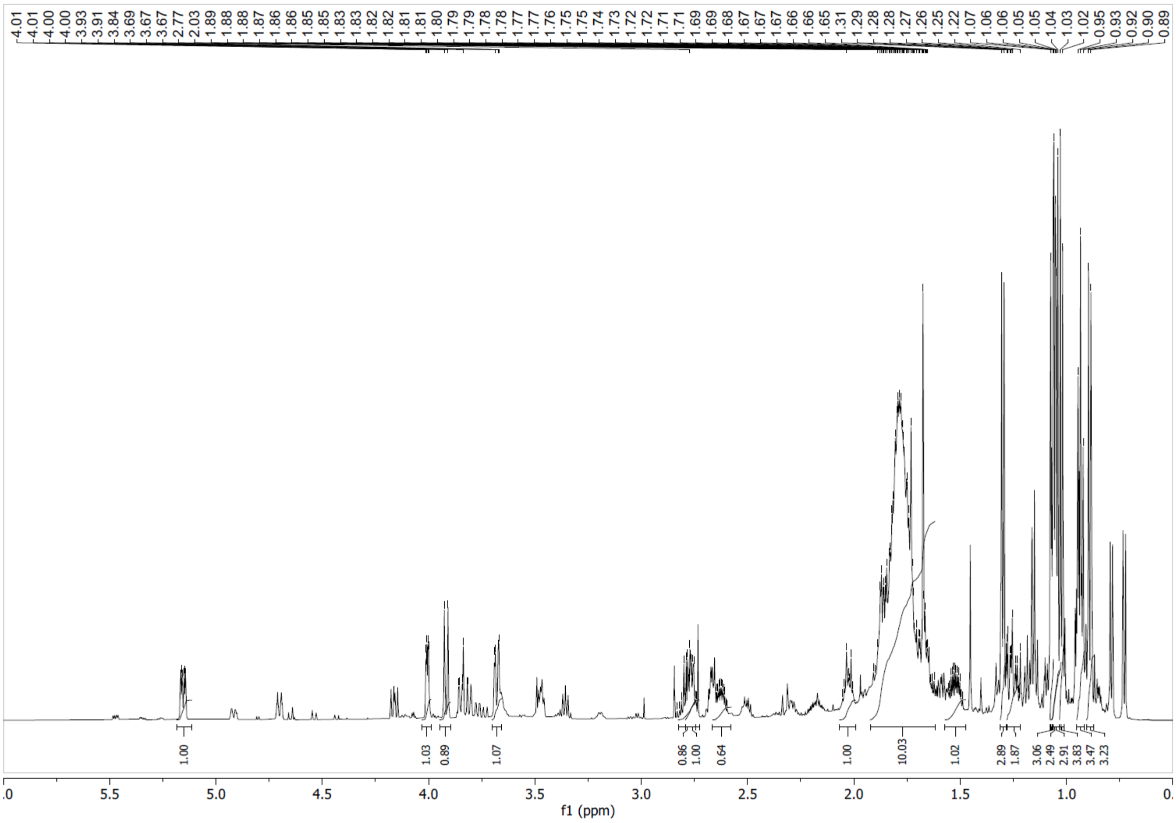

Figure S11 : ^{1}H NMR spectrum of 6-deoxyerythronolide B (**2**) in $CDCl_3$ at 600 MHz.

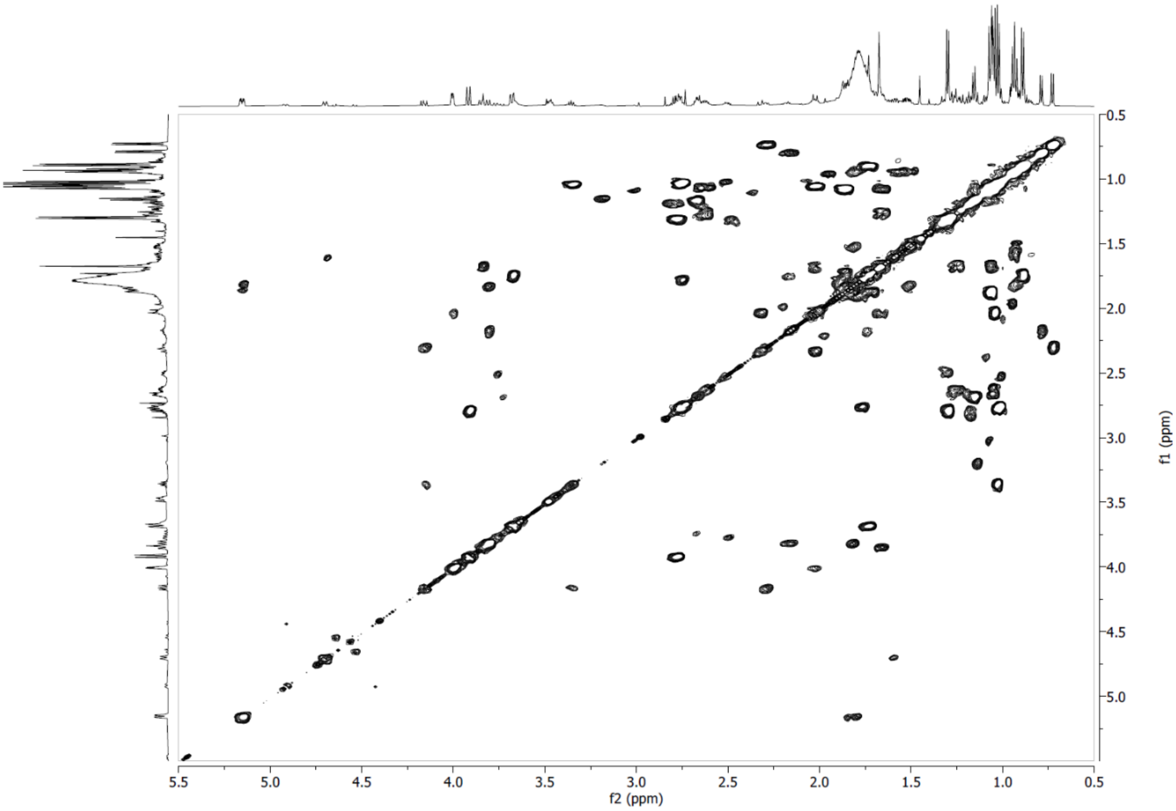

Figure S12: COSY NMR spectrum of 6-deoxyerythronolide B (**2**) in $CDCl_3$.

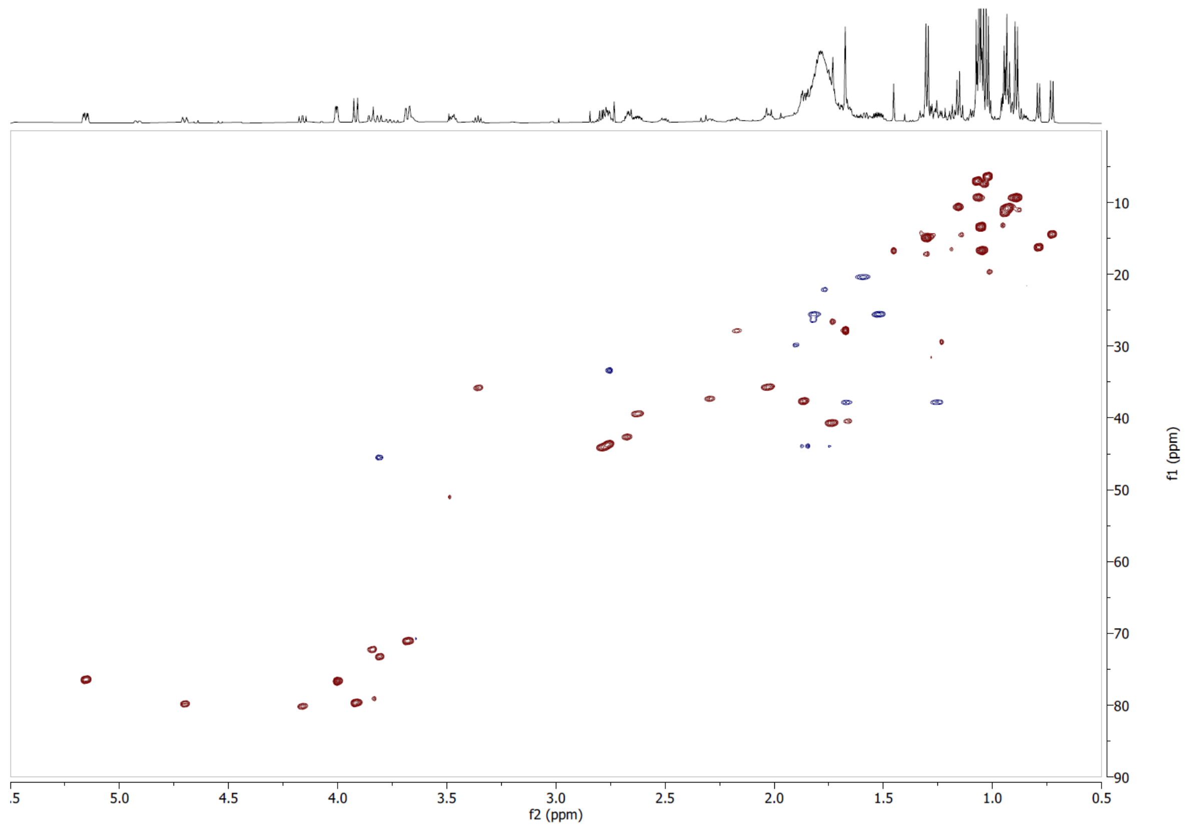

Figure S13: Edited HSQC NMR spectrum of 6-deoxyerythronolide B (**2**) in $CDCl_3$.

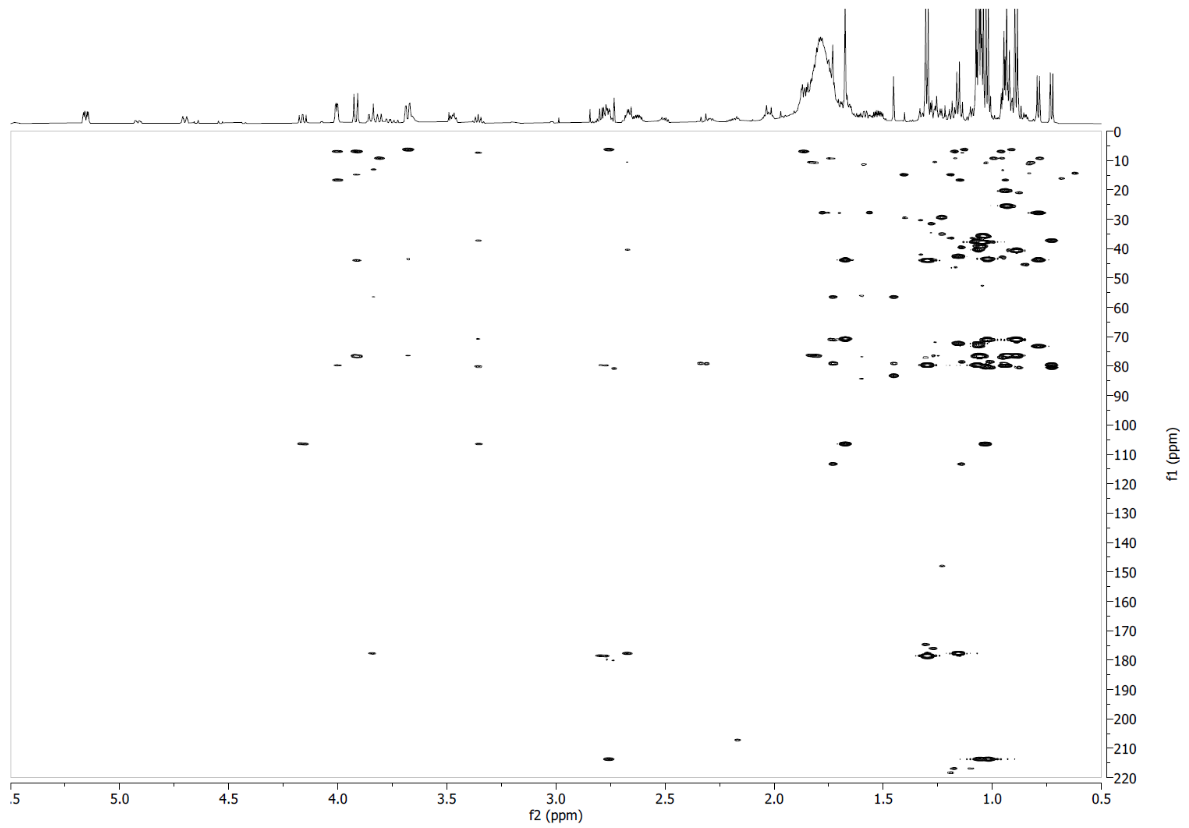

Figure S14: HMBC NMR spectrum of 6-deoxyerythronolide B (**2**) in $CDCl_3$.

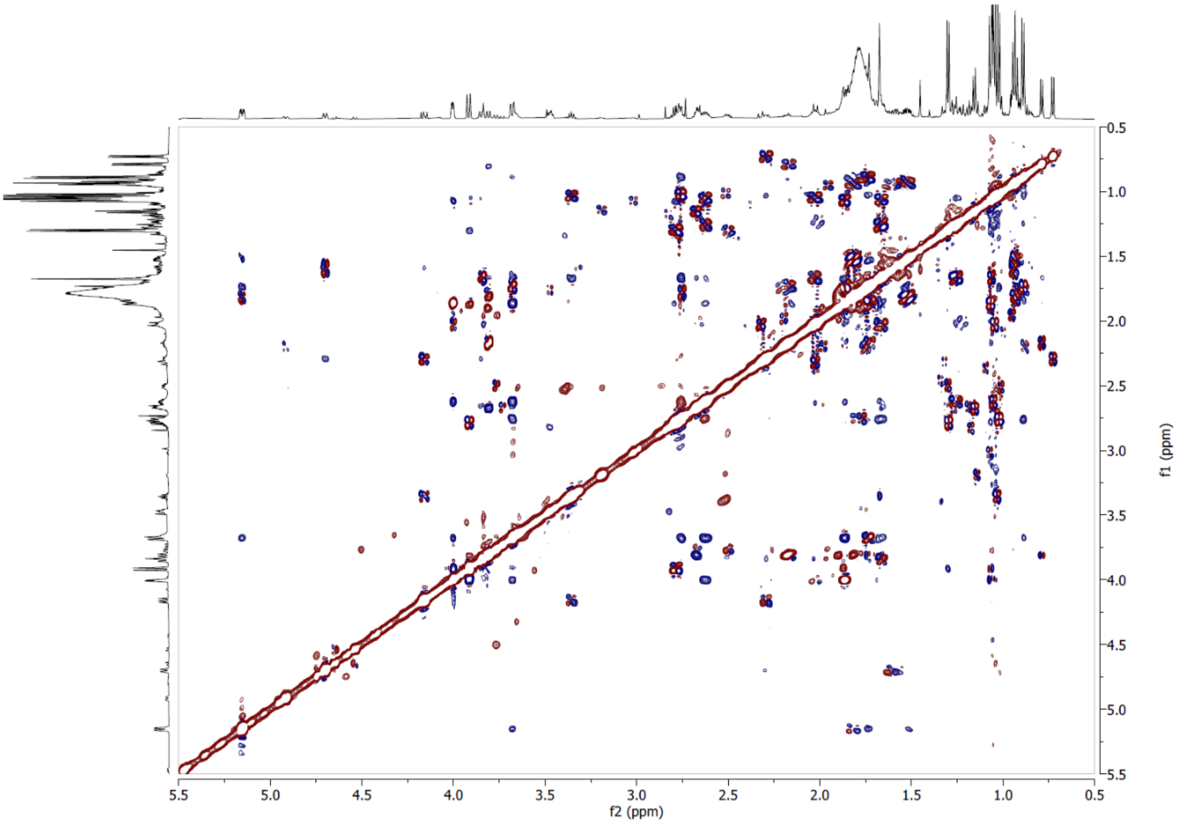

Figure S15: ROESY NMR spectrum of 6-deoxyerythronolide B (**2**) in $CDCl_3$.

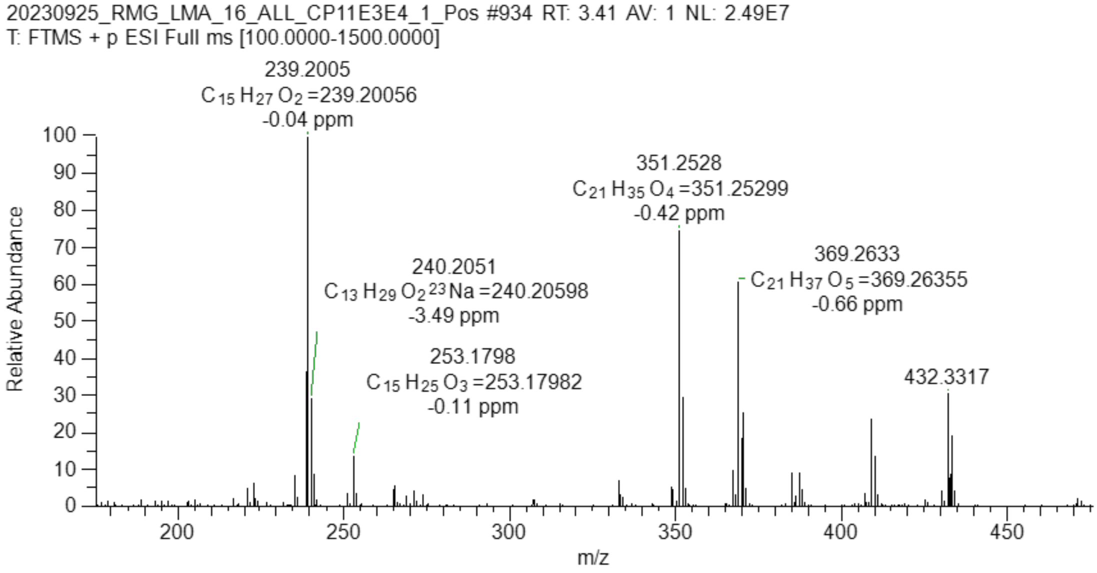

Figure S16: ESI⁺-HRMS data in positive mode of 6-deoxyerythronolide B (**2**).

Table S3: ^{1}H and ^{13}C NMR chemical shifts of compound **3** in CD_3OD and ^{13}C NMR chemical shifts of megalomicin C1 reported by Bartner, P. *et al*. (1979) in $CDCl_3$. n.o.: not observed.

	Compound **3** in CD_3OD		Megalomicin C1 [35] in $CDCl_3$
No	δ_H (Multiplicity, *J*)	δ_C	δ_C
1	-	178.1	175.8
2	2.94 (dq, 10.4,7.2 Hz)	46.3	44.8
2-CH_3	1.20 (d, 7.2 Hz)	16.8	18.8
3	4.55 (dd, 10.4,1.5 Hz)	83.1	81.8
4	2.04 (pd, 7.6,6.2,1.5 Hz)	39.1	37.2
4-CH_3	1.14 (d, 7.6 Hz)	10.5	9.8
5	3.95 (d, 6.2 Hz)	83.6	83.0
6	-	82.3	80.1
6-CH_3	1.58 (s)	19.8	16.2
7	2.05 (d, 15.1 Hz)<br>1.70 (d, 15.1 Hz)	39.8	39.0
8	3.23 (q, 6.9 Hz)	38.9	45.9
8-CH_3	1.15 (d, 7.0 Hz)	12.5	15.0
9	-	n.o.	221.4
10	2.55 (m)	46.9	37.4
10-CH_3	1.15 (d, 7.0 Hz)	19.2	12.4
11	3.61 (d, 1.4 Hz)	70.5	68.4
12	-	76.0	74.4
12-CH_3	1.19 (s)	17.6	16.2
13	5.19 (dd, 11.3,2.3 Hz)	78.6	76.6
14	1.90 (qd, 7.4,2.3 Hz)<br>1.54 (dq, 11.3,7.4 Hz)	22.2	21.2
15	0.85 (t, 7.4 Hz)	10.9	10.4
D-desosamine			
1'	4.55 (d, 7.1 Hz)	103.6	102.6
2'	3.38 (d, 7.1 Hz)	71.5	71.5
3'	n.o.	n.o.	65.5
4'	1.91 (m)<br>1.41 (m)	31.7	29.1
5'	3.65 (p, 10.4,6.3 Hz)	69.1	68.9
6'	1.28 (d, 6.0 Hz)	22.0	21.8
3'N(CH_3)$_2$	2.55/2.84	41.8	40.2
L-megosamine			
1''	4.98 (t, 6.8,5.6 Hz)	91.0	90.4
2''	3.34 (overlapped)<br>1.83 (m)	30.6	28.0

Table S3: *Continued.*

	Compound **3** in CD_3OD		Megalomicin C1 [35] in $CDCl_3$
No	δ_H (Multiplicity, *J*)	δ_C	δ_C
3''	no	62.8	59.4
4''	3.82 (t, 1.8,1.4 Hz)	66.3	73.5
5''	4.30 (qd, 7.4,1.4 Hz)	75.1	67.5
6''	1.23 (d, 7.4 Hz)	15.2	18.8
3'N(CH_3)$_2$	2.55/2.84	41.8	42.5
		L-mycarose	
1'''	5.03 (d, 4.5 Hz)	99.0	97.5
2'''	3.34 (overlapped)<br>1.83 (m)	37.3	36.4
3'''	-	79.7	78.0
3'''-CH_3	1.45 (s)	22.9	23.1
4'''	4.60 (d, 9.6 Hz)	78.9	78.0
5'''	4.25 (dq, 9.6,6.1 Hz)	64.4	62.7
6'''	1.20 (d, 6.1 Hz)	19.2	18.4
3'''a	-	172.7	170.5
3'''b	2.13 (s)	20.7	22.8
4'''a	-	172.0	170.0
4'''b	2.13 (s)	23.6	20.7

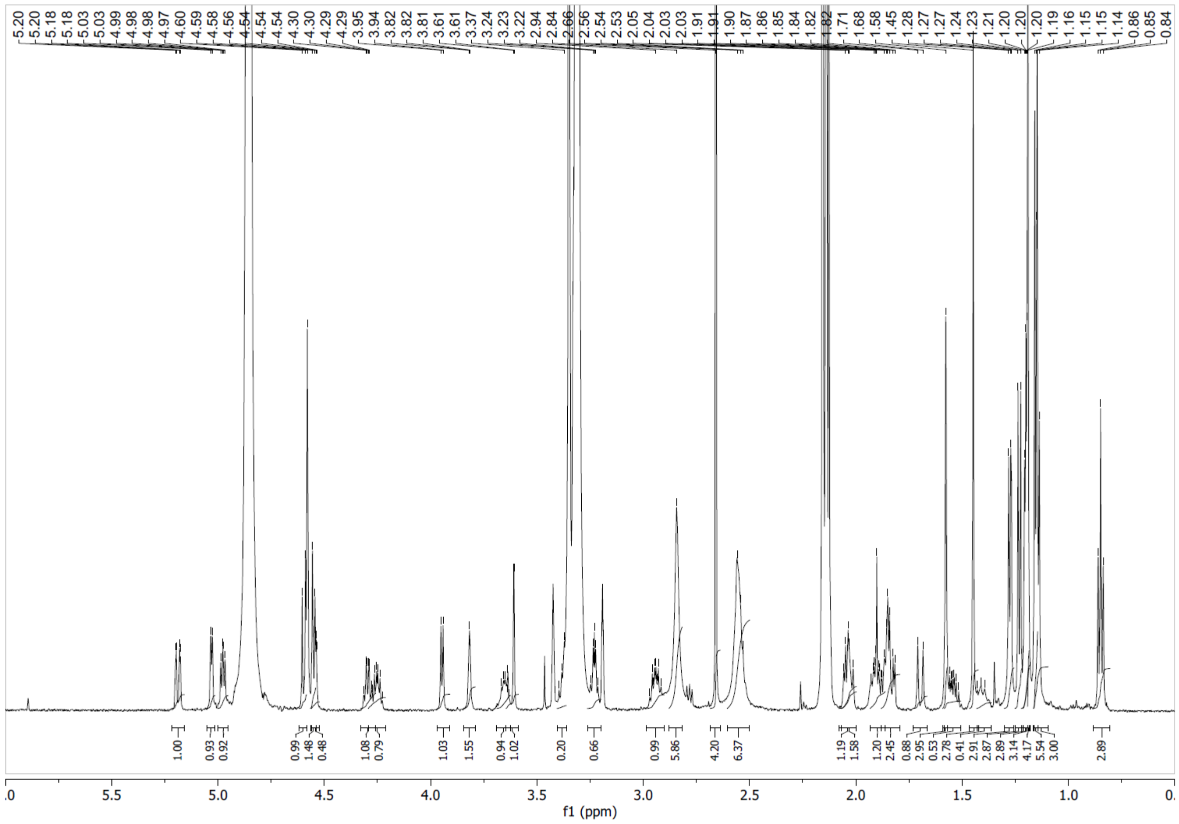

Figure S17 : ^{1}H NMR spectrum of megalomicin C1 (**3**) in CD_3OD at 600 MHz.

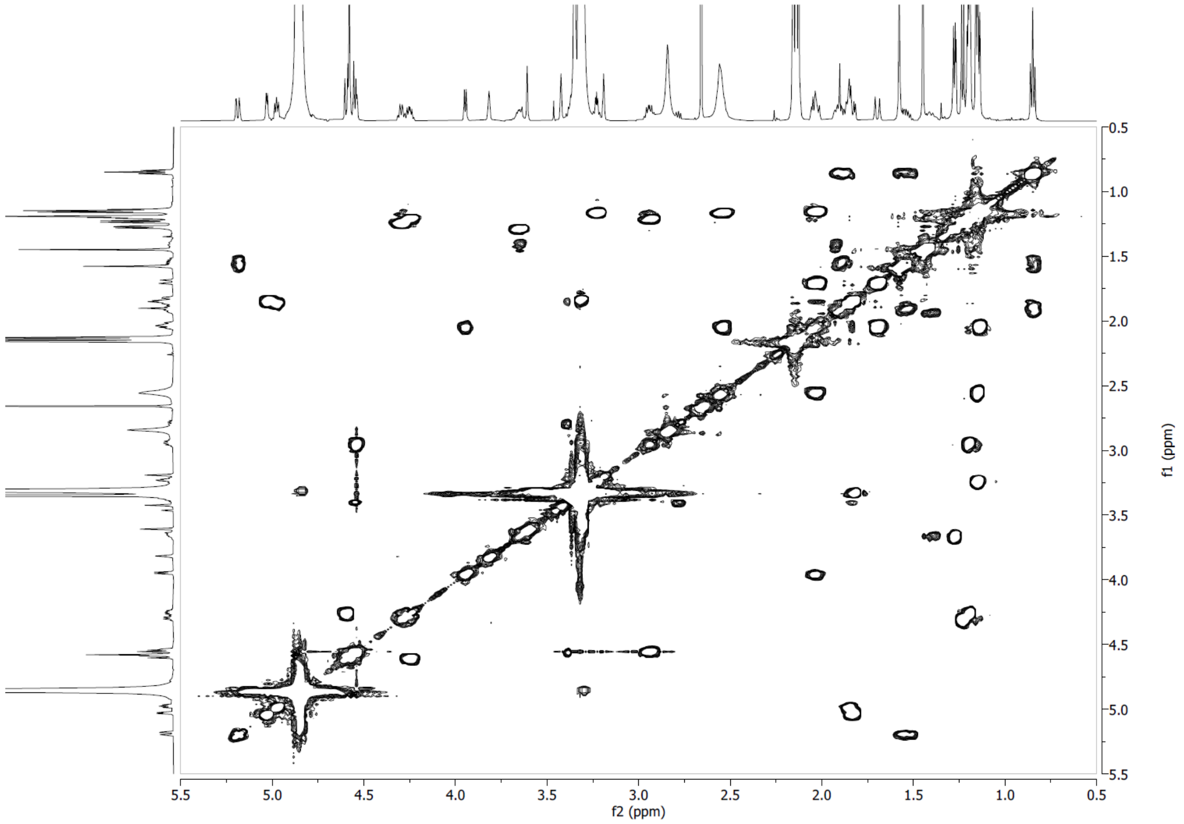

Figure S18 : COSY NMR spectrum of megalomicin C1 (**3**) in CD_3OD.

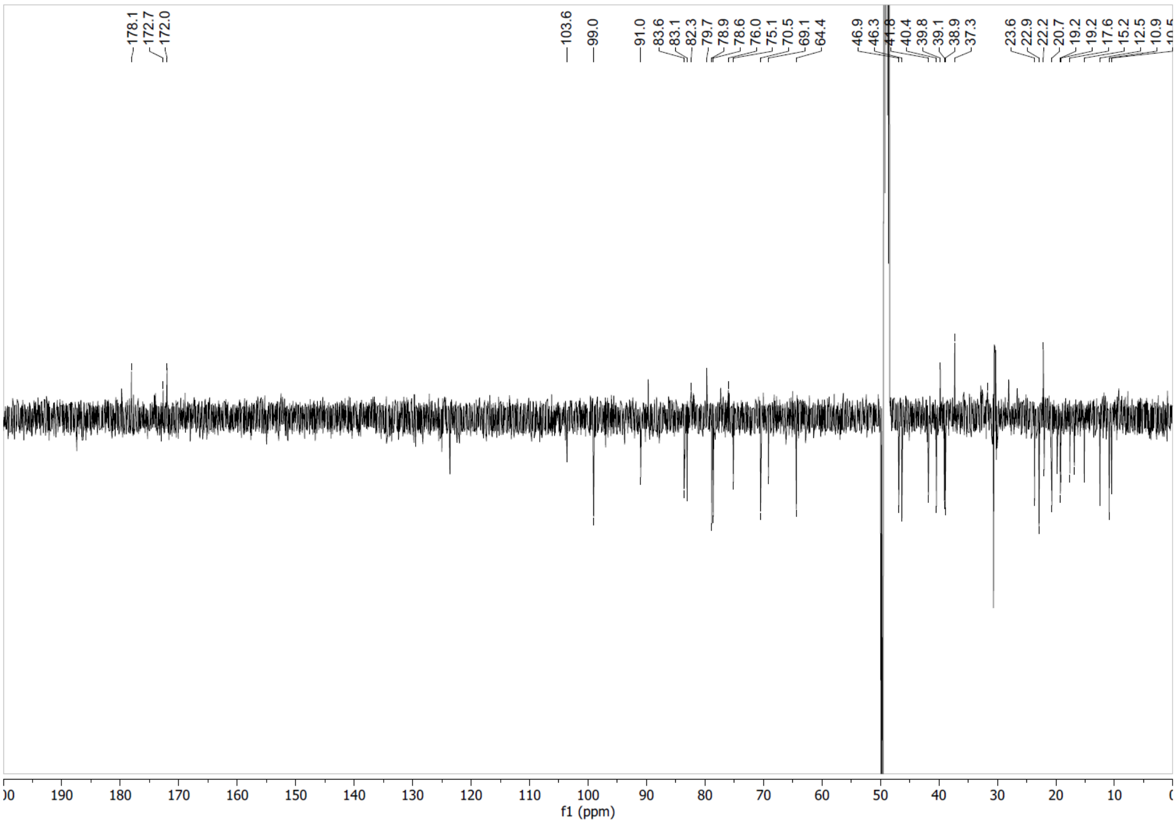

Figure S19 : ^{13}C-DEPTQ NMR spectrum of megalomicin C1 (**3**) in CD_3OD at 151 MHz.

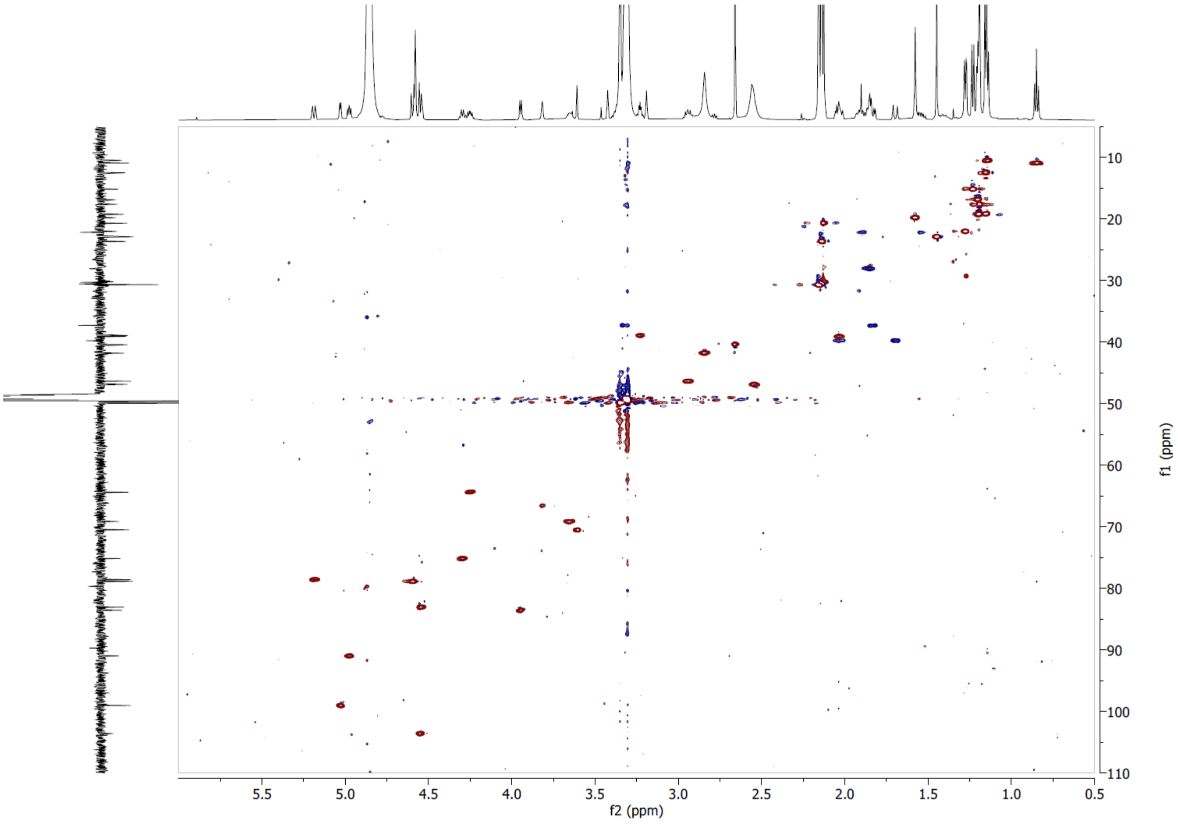

Figure S20 : Edited HSQC NMR spectrum of megalomicin C1 (**3**) in CD_3OD.

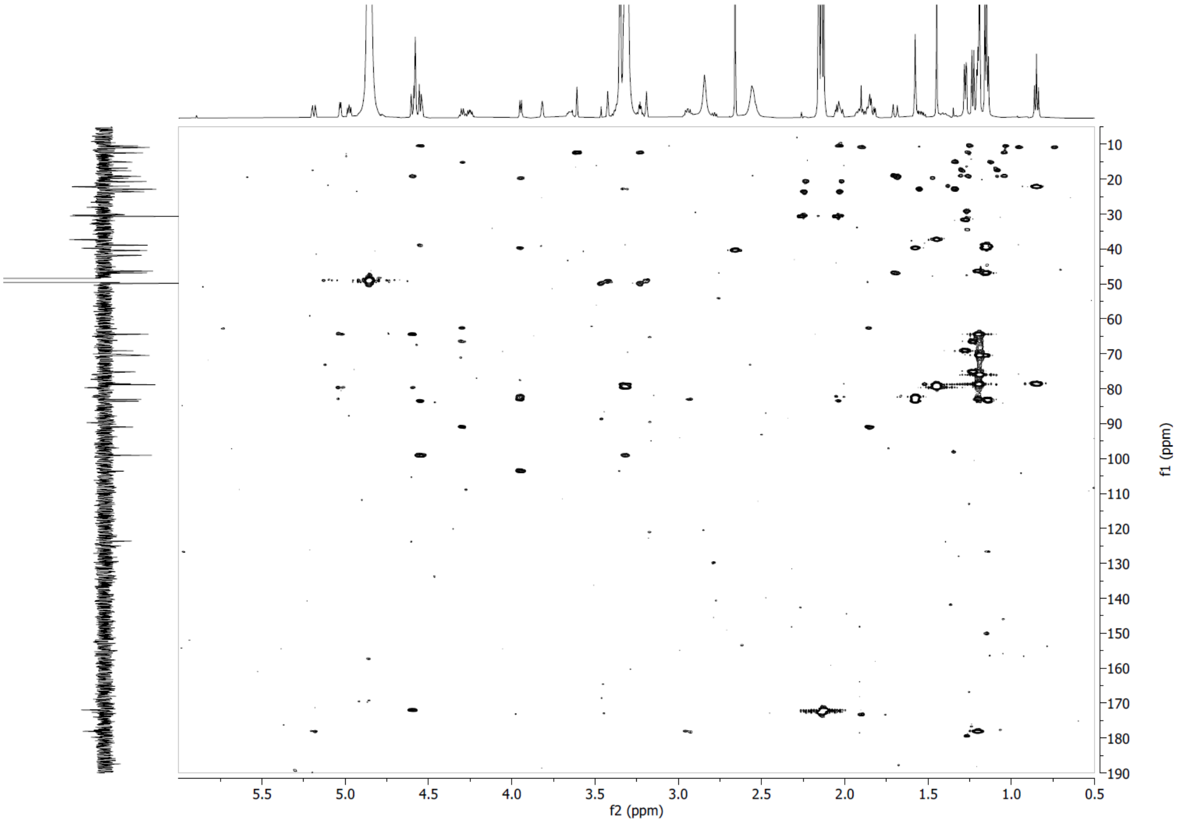

Figure S21 : HMBC NMR spectrum of megalomicin C1 (**3**) in CD_3OD.

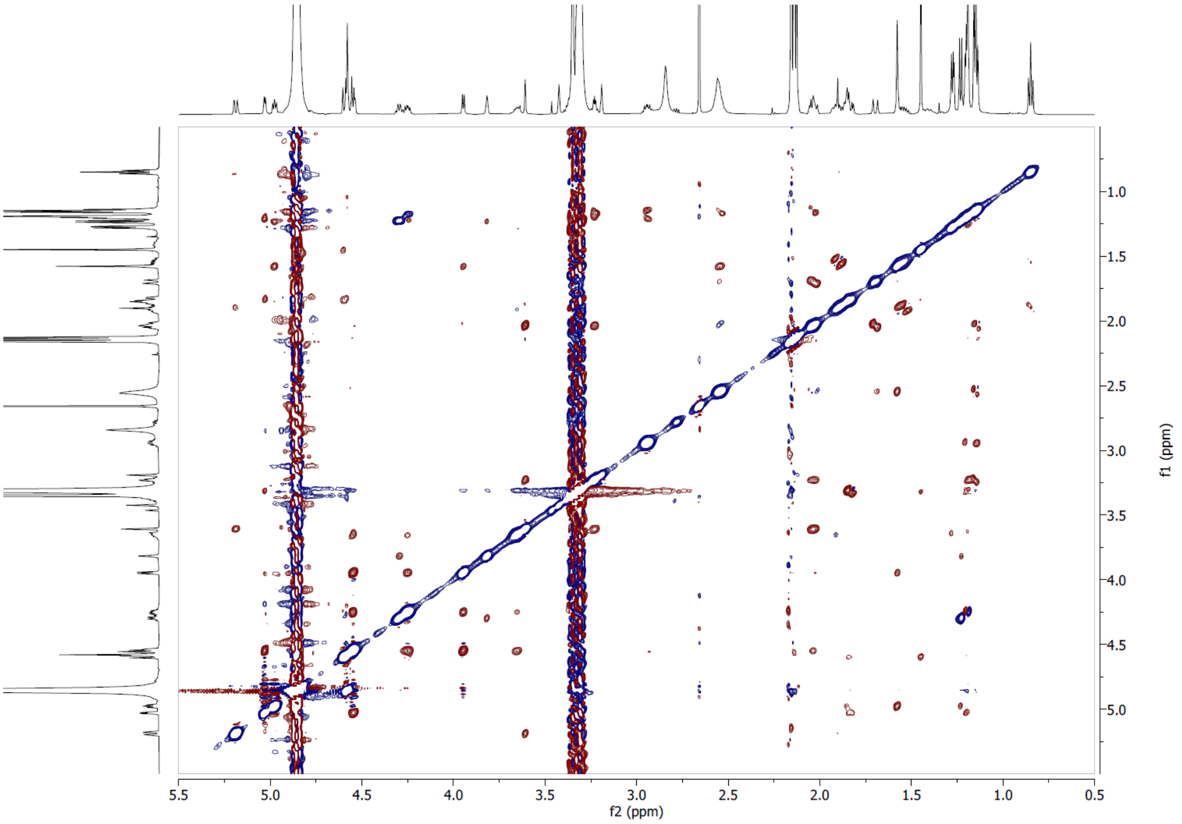

Figure S22 : ROESY NMR spectrum of megalomicin C1 (**3**) in CD_3OD.

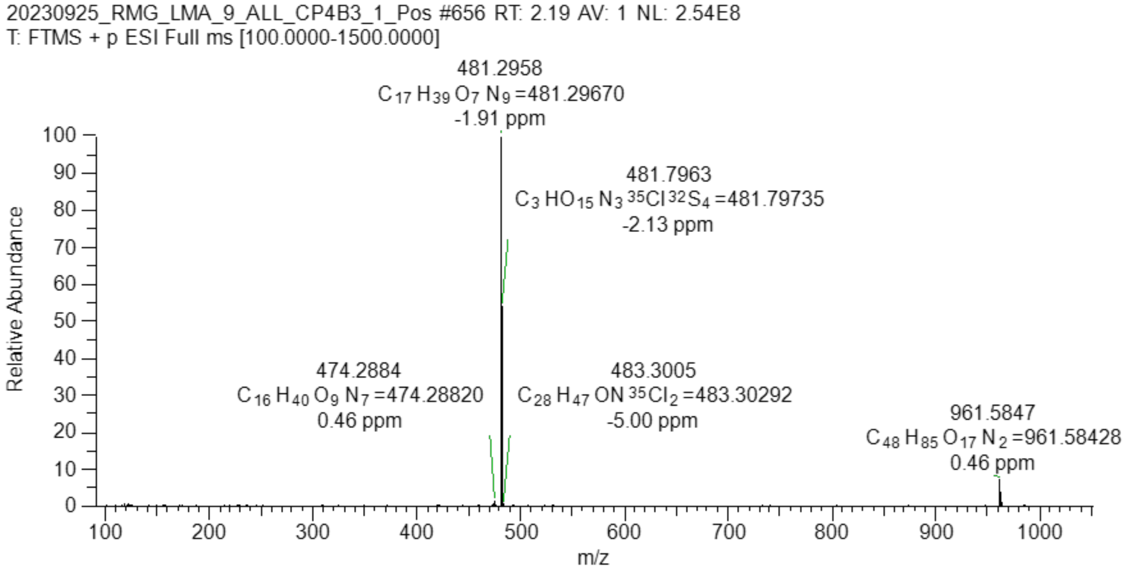

Figure S23 : ESI⁺-HRMS data in positive mode of megalomicin C1 (**3**).

Table S4 : ^{1}H and ^{13}C NMR chemical shifts of compound **4** in CD_3OD and ^{13}C NMR chemical shifts of 6,9-hemiacetal-8,9-anhydroerythonolide B reported by Nourse J.G. and Roberts J.D. (1975) in 4:1 (v/v) mixtures of $CDCI_3$ and CH_2CI_2.

	Compound **4** in CD_3OD		6,9-hemiacetal-8,9-anhydroerythonolide B [33] in 4:1 (v/v) mixtures of $CDCI_3$ and CH_2CI_2
No	δ_H (Multiplicity, *J*)	δ_C	δ_C
1	-	177.5	176.8
2	2.66 (dq, 10.4,6.7 Hz)	44.9	44.6
2-CH_3	1.16 (d, 6.7 Hz)	14.6	14.2
3	3.49 (dd, 10.4,1.4 Hz)	82.8	82.3
4	2.06 (qd, 7.0,1.4 Hz)	36.2	35.2
4-CH_3	0.95 (d, 7.0 Hz)	7.2	6.3
5	3.56 (s)	83.1	82.3
6	-	85.1	84.2
6-CH_3	1.34 (s)	28.9	28.5
7	2.81 (dq, 15.7,1.3 Hz)<br>2.07 (dq, 15.7,1.3 Hz)	43.3	42.5
8	-	102.5	101.9
8-CH_3	1.55 (t, 1.3 Hz)	12.5	12.2
9	-	152.5	151.4
10	2.61 (dq, 9.2,7.1 Hz)	35.4	34.1
10-CH_3	1.07 (d, 7.1 Hz)	16.3	15.3
11	3.56 (t, 9.2 Hz)	71.9	70.9
12	1.59 (dq, 9.2,7.5 Hz)	47.6	46.1
12-CH_3	0.93 (d, 7.5 Hz)	9.2	8.5
13	5.41 (dd, 9.5,4.9 Hz)	78.5	78.2
14	1.69 (ddq, 14.0,9.5,7.4 Hz)<br>1.48 (dqd, 14.0,7.4,4.9 Hz)	27.0	26.1
15	0.87 (t, 7.4 Hz)	10.8	10.5

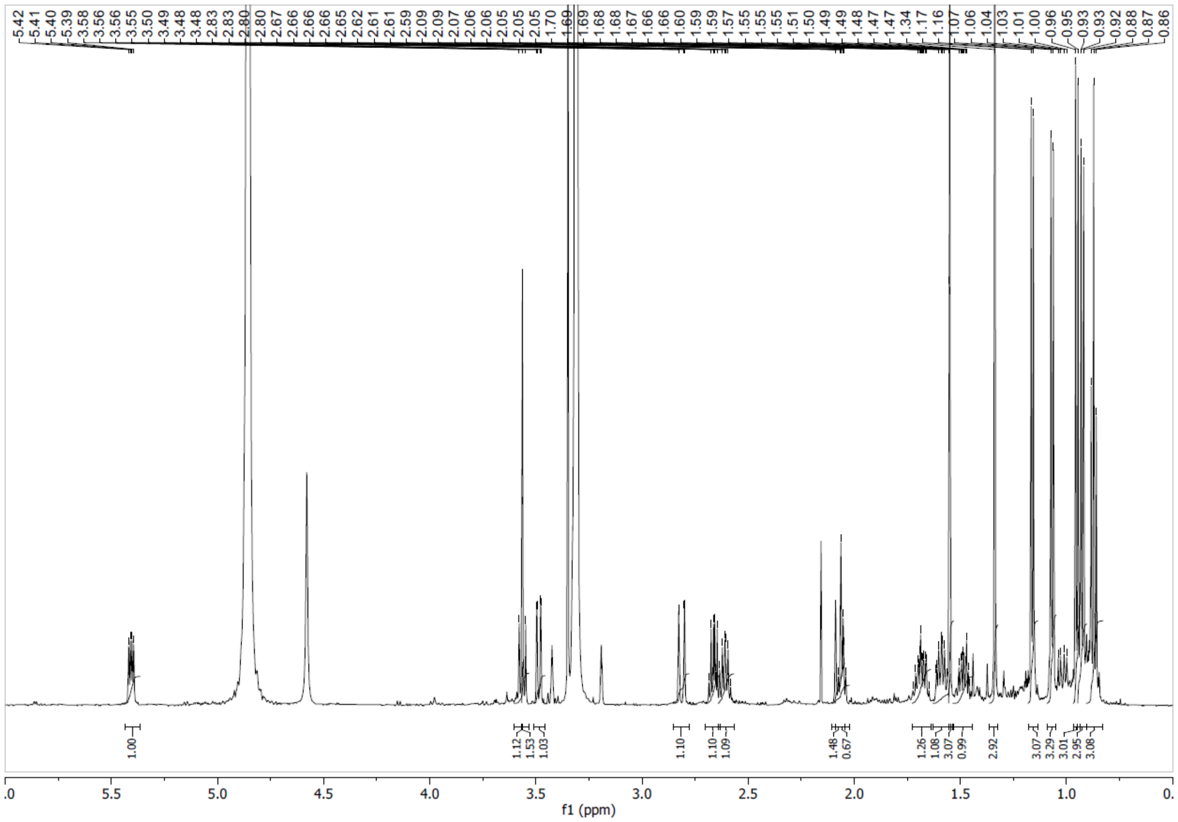

Figure S24 : ^{1}H NMR spectrum of 6,9-hemiacetal-8,9-anhydroerythonolide B (**4**) in CD_3OD at 600 MHz.

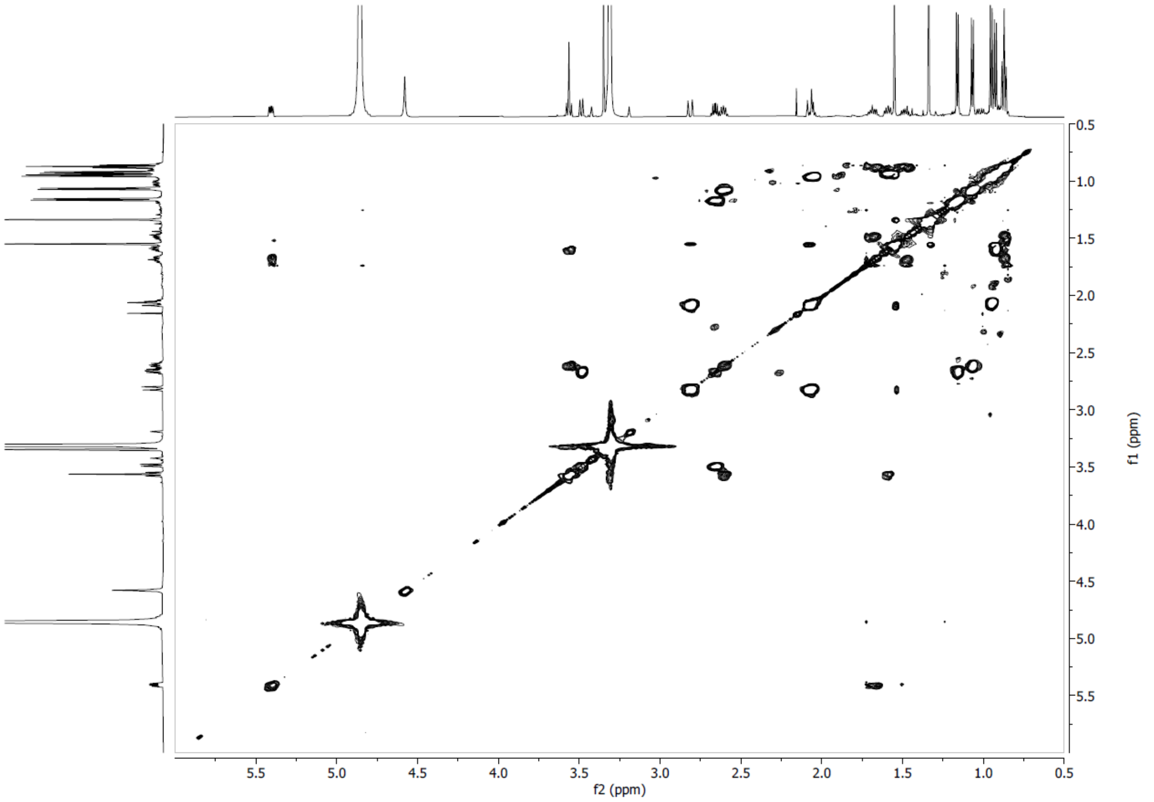

Figure S25 : COSY NMR spectrum of 6,9-hemiacetal-8,9-anhydroerythonolide B (**4**) in CD_3OD.

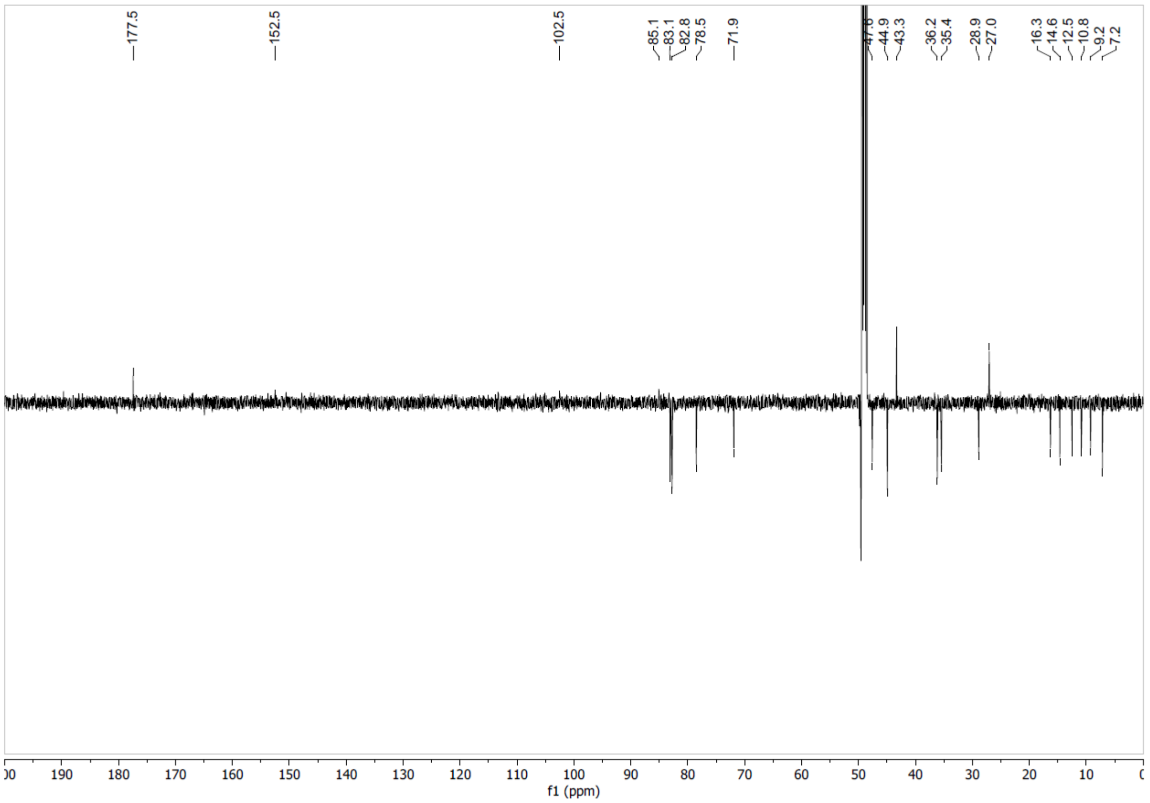

Figure S26 : ^{13}C-DEPTQ NMR spectrum of 6,9-hemiacetal-8,9-anhydroerythonolide B (**4**) in CD_3OD at 151 MHz.

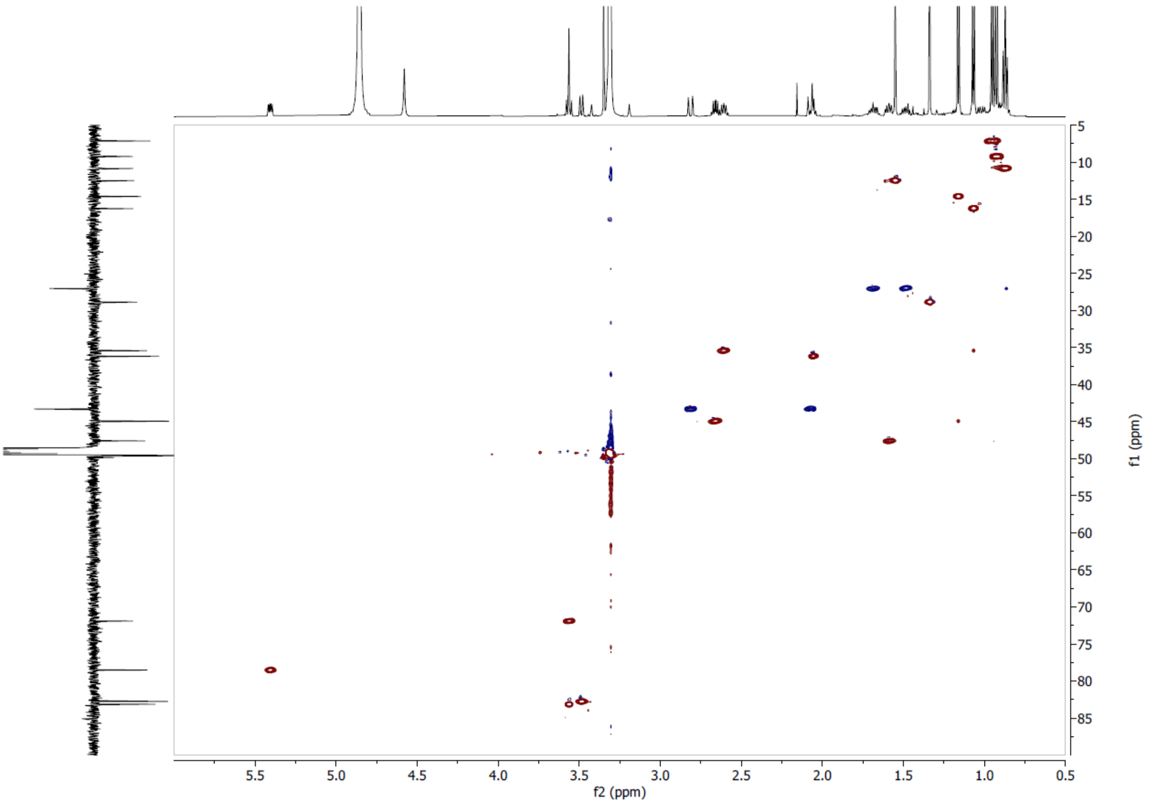

Figure S27 : Edited HSQC NMR spectrum of 6,9-hemiacetal-8,9-anhydroerythonolide B (**4**) in CD_3OD.

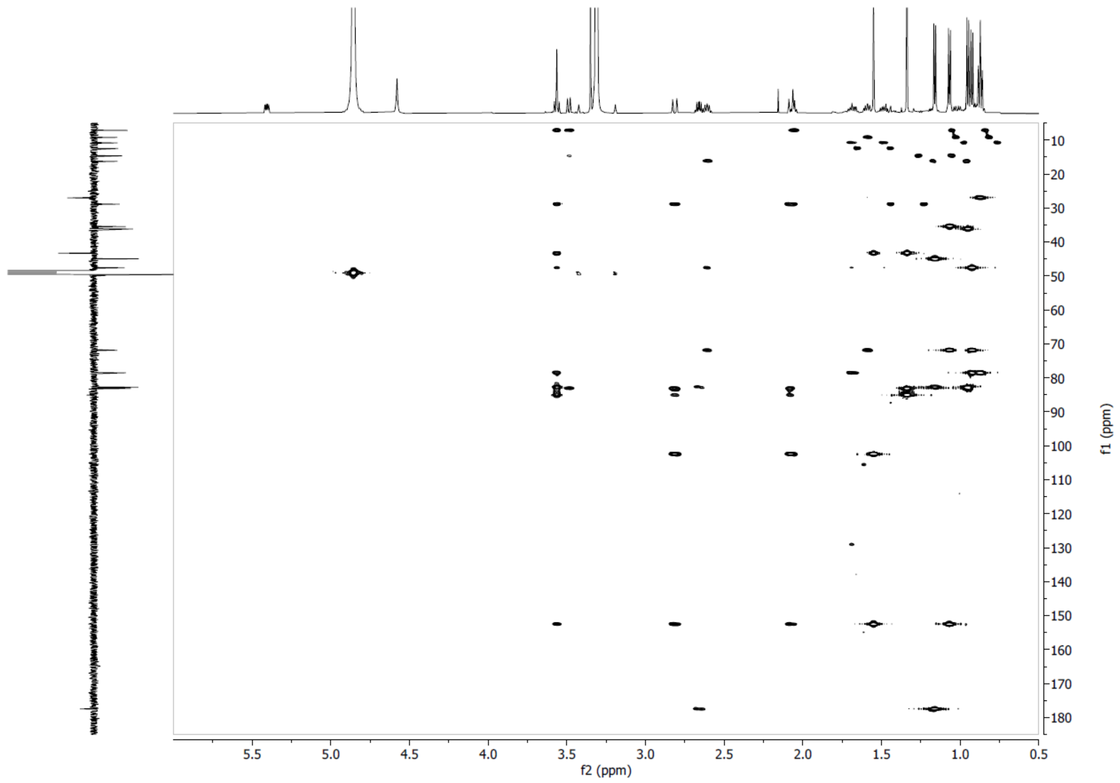

Figure S28 : HMBC NMR spectrum of 6,9-hemiacetal-8,9-anhydroerythonolide B (**4**) in CD_3OD.

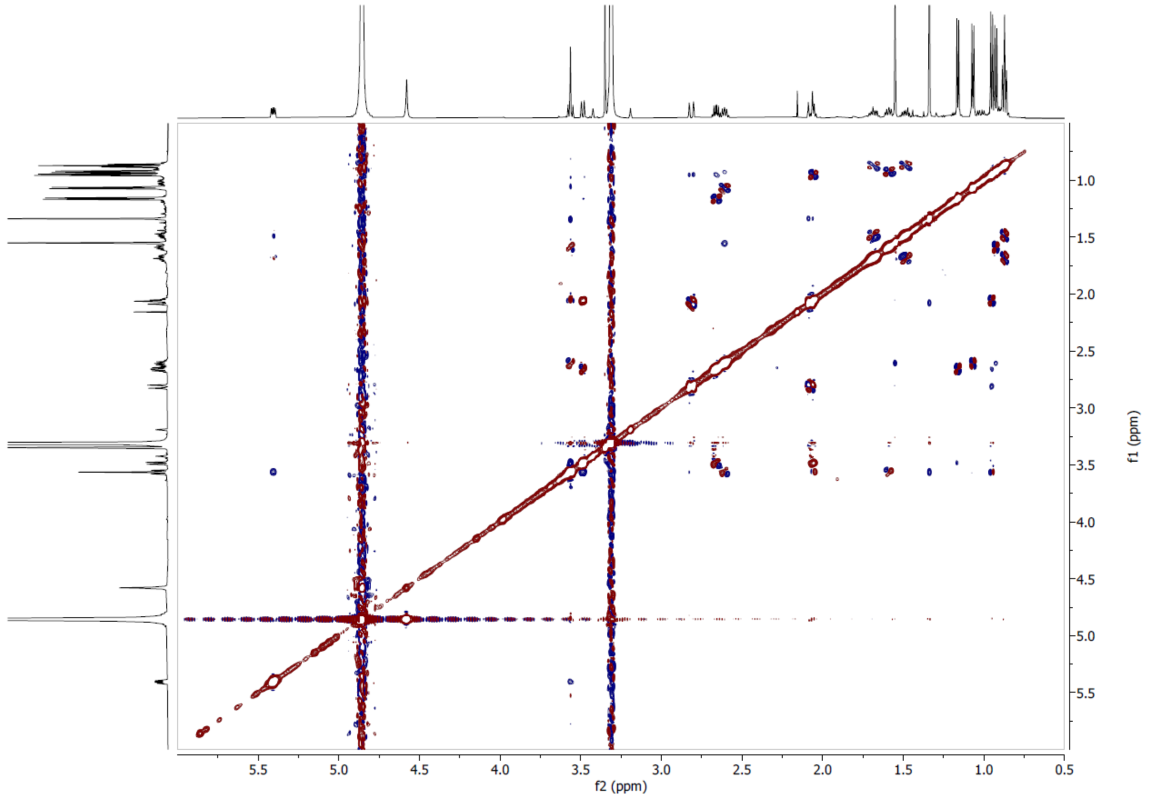

Figure S29 : ROESY NMR spectrum of 6,9-hemiacetal-8,9-anhydroerythonolide B (**4**) in CD_3OD.

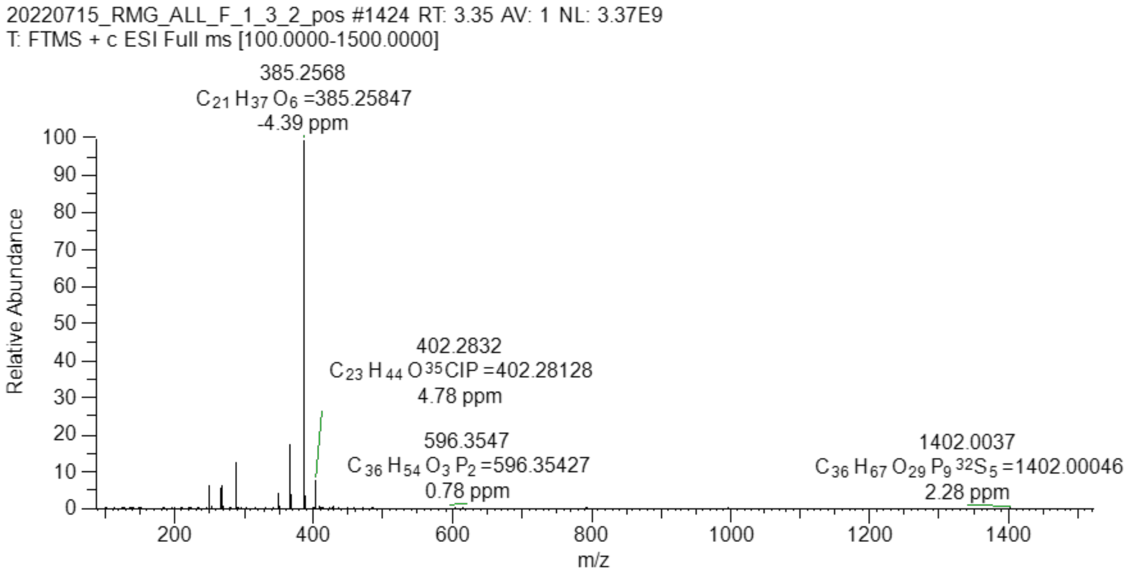

Figure S30 : ESI+-HRMS data in positive mode of 6,9-hemiacetal-8,9-anhydroerythonolide B (**4**).

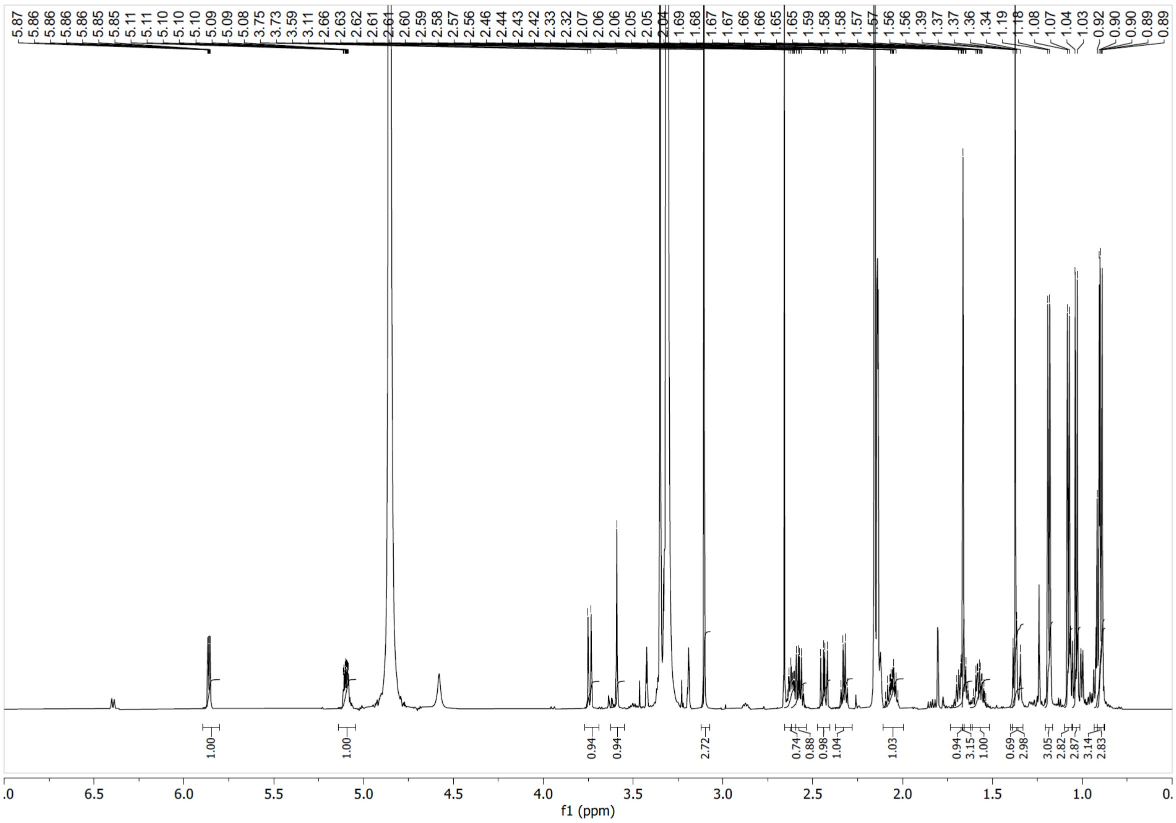

Figure S31 : ^{1}H NMR spectrum of 6,9-hemiacetal-9-*O*-methyl-10,11-anhydroerythronolide B (**5**) in CD_3OD at 600 MHz.

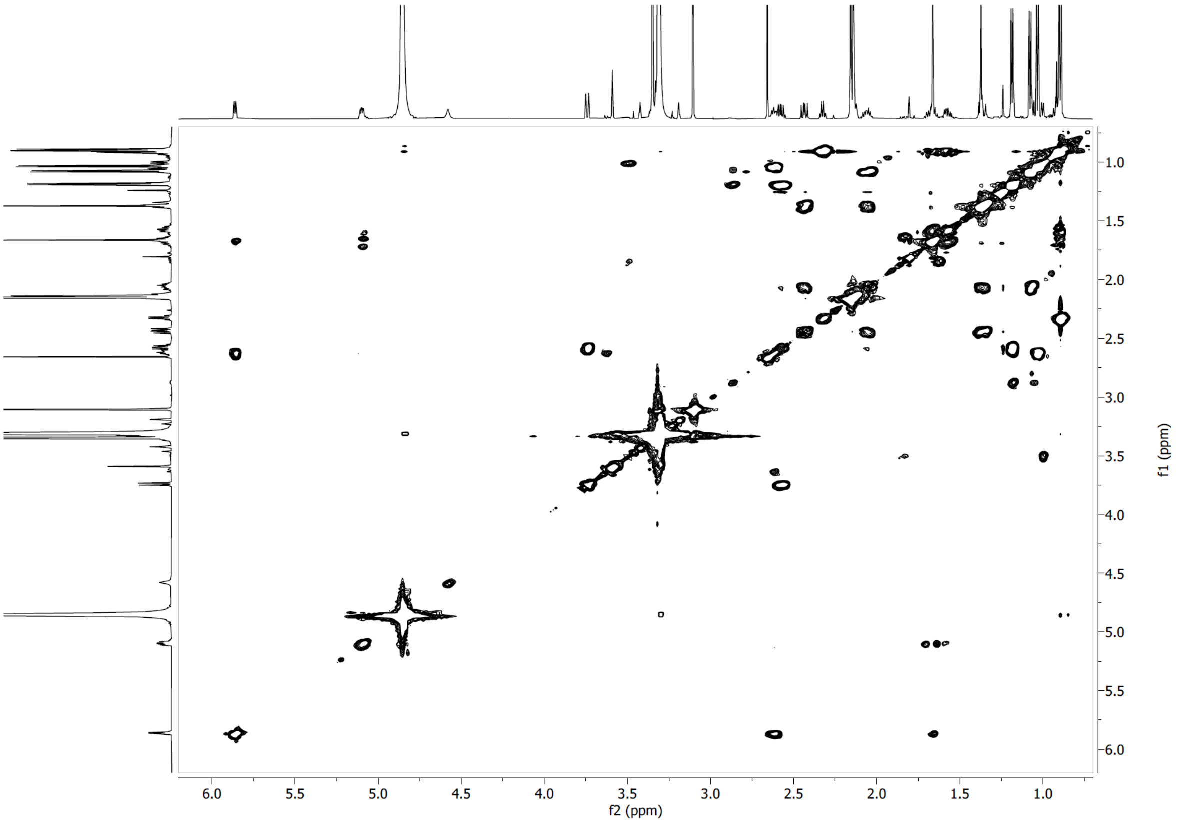

Figure S32 : COSY NMR spectrum of 6,9-hemiacetal-9-*O*-methyl-10,11-anhydroerythronolide B (**5**) in CD_3OD.

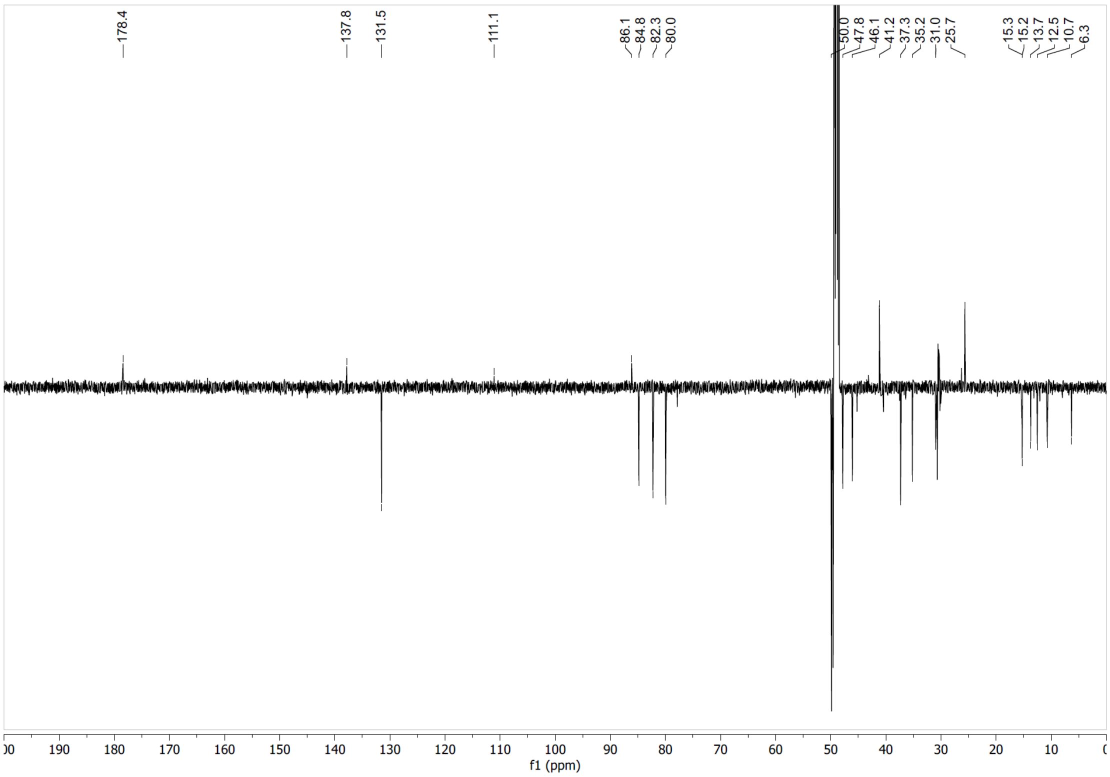

Figure S33 : ^{13}C-DEPTQ NMR spectrum of 6,9-hemiacetal-9-*O*-methyl-10,11-anhydroerythronolide B (compound **5**) in CD_3OD at 151 MHz.

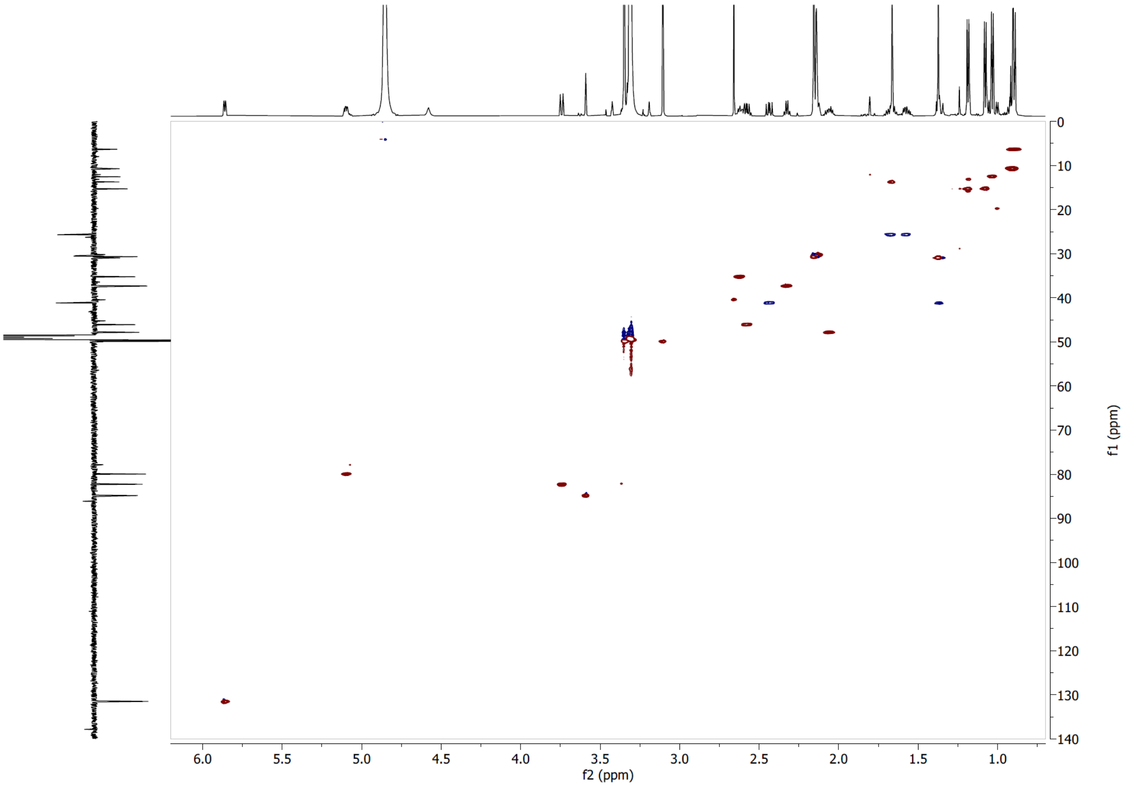

Figure S34 : Edited HSQC NMR spectrum of 6,9-hemiacetal-9-*O*-methyl-10,11-anhydroerythronolide B (**5**) in CD_3OD.

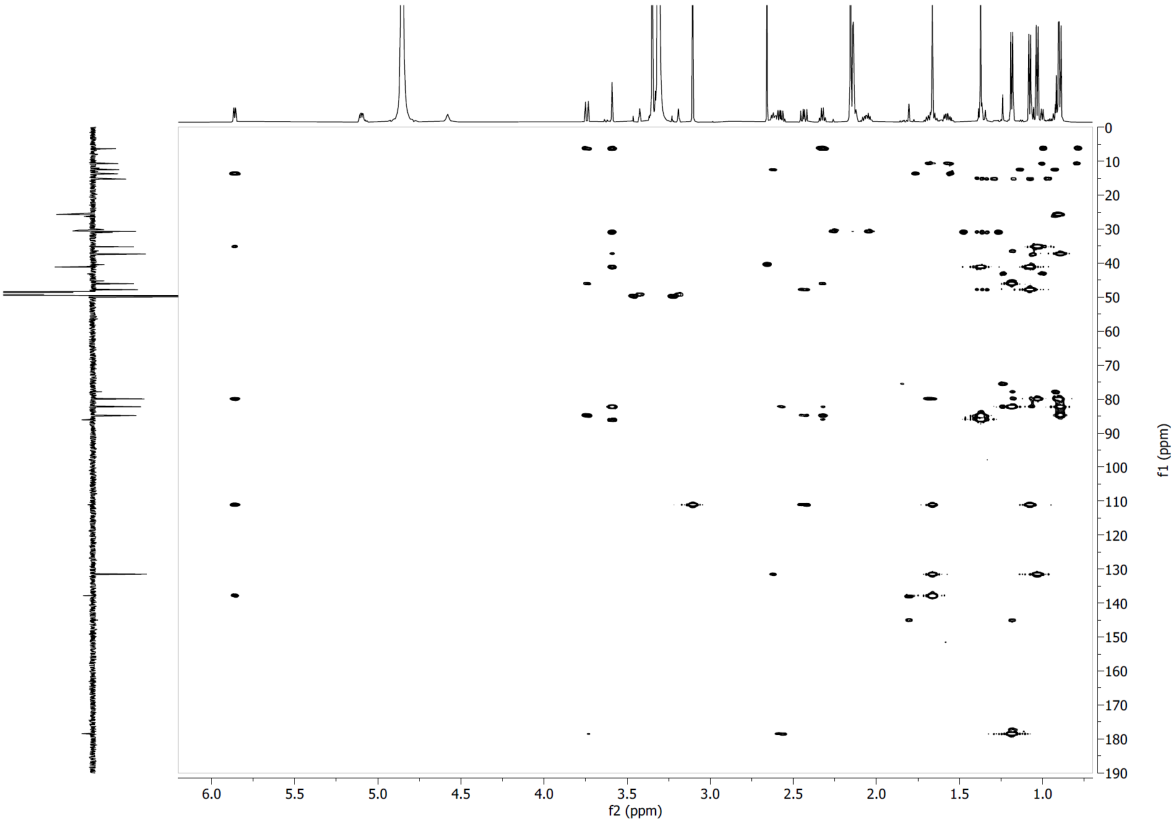

Figure S35 : HMBC NMR spectrum of 6,9-hemiacetal-9-*O*-methyl-10,11-anhydroerythronolide B (**5**) in CD_3OD.

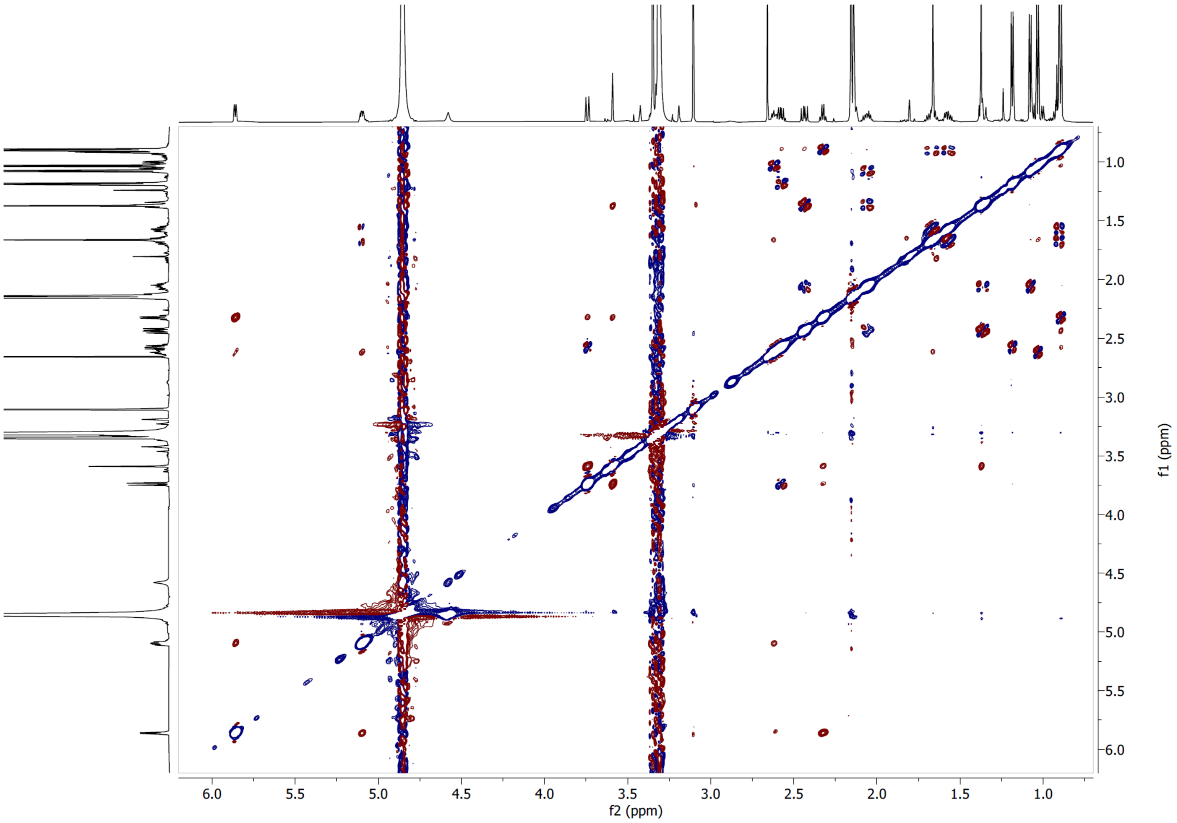

Figure S36 : ROESY NMR spectrum of 6,9-hemiacetal-9-*O*-methyl-10,11-anhydroerythronolide B (**5**) in CD_3OD.

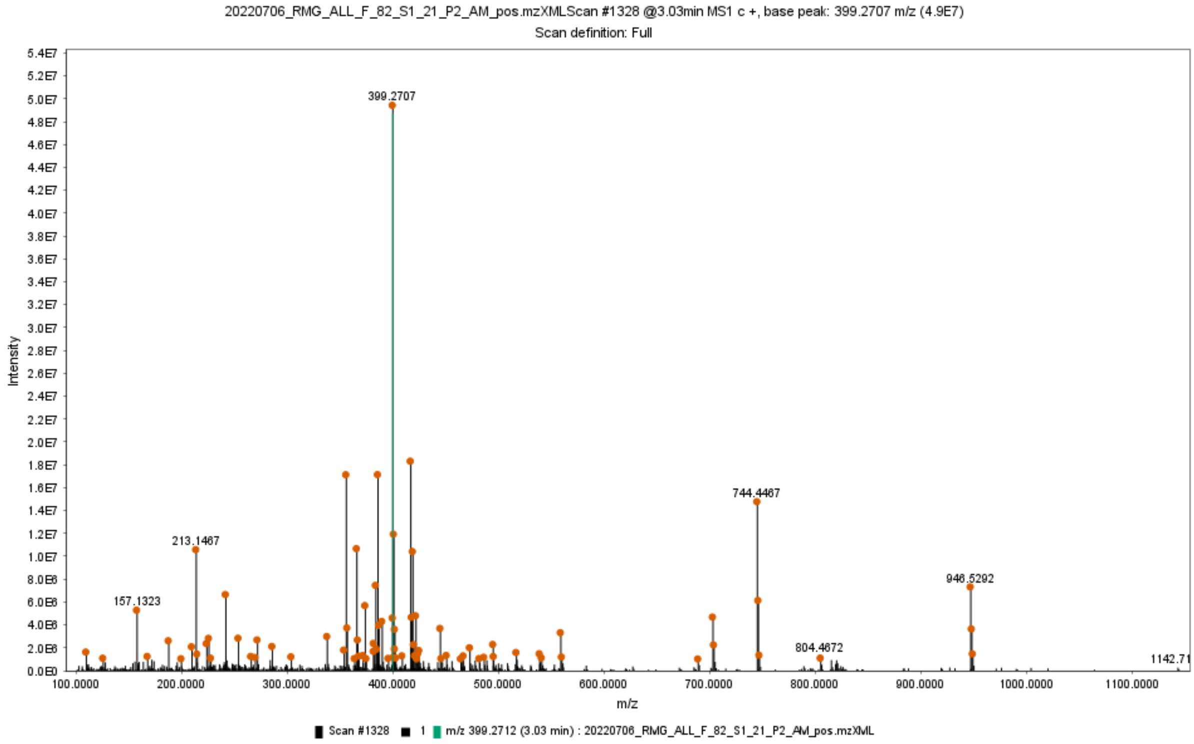

Figure S37 : ESI$^+$-HRMS data in positive mode of 6,9-hemiacetal-9-*O*-methyl-10,11-anhydroerythronolide B (**5**) in microbial raw extract.

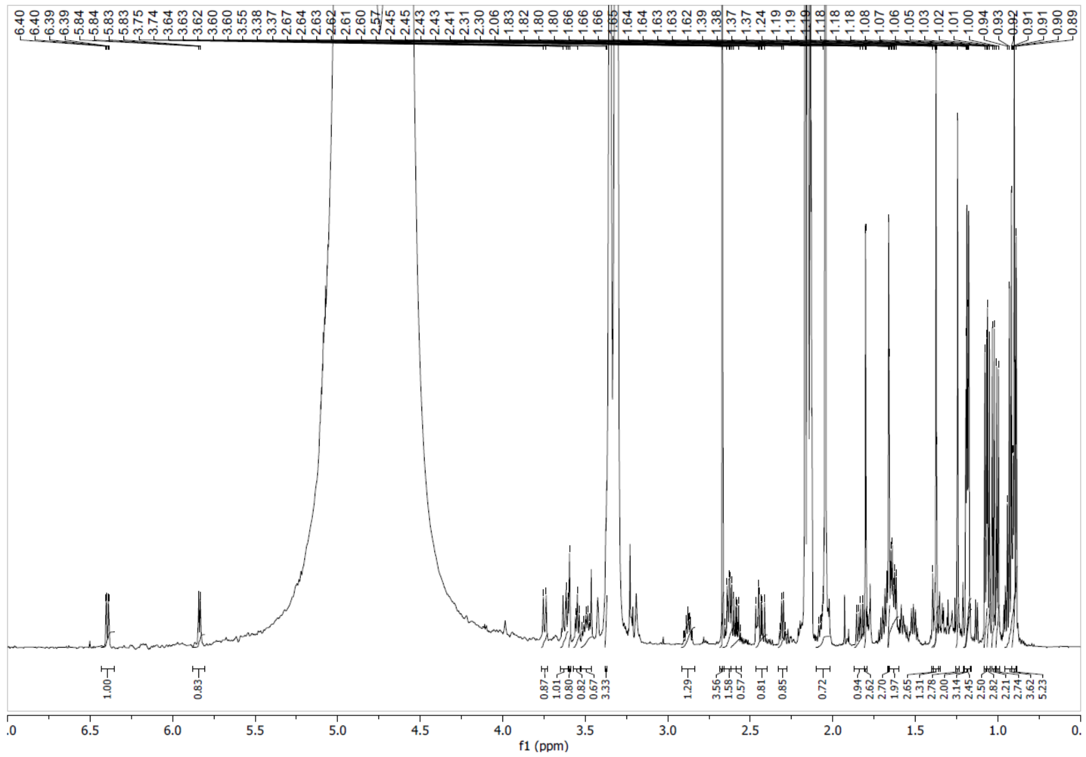

Figure S38 : ^{1}H NMR spectrum of 6,9-hemiacetal-9-hydroxy-10,11-anhydroerythronolide B (**5.1**) and 8-*epi*-10,11-anhydroerythronolide B (**5.2**) in CD_3OD at 600 MHz.

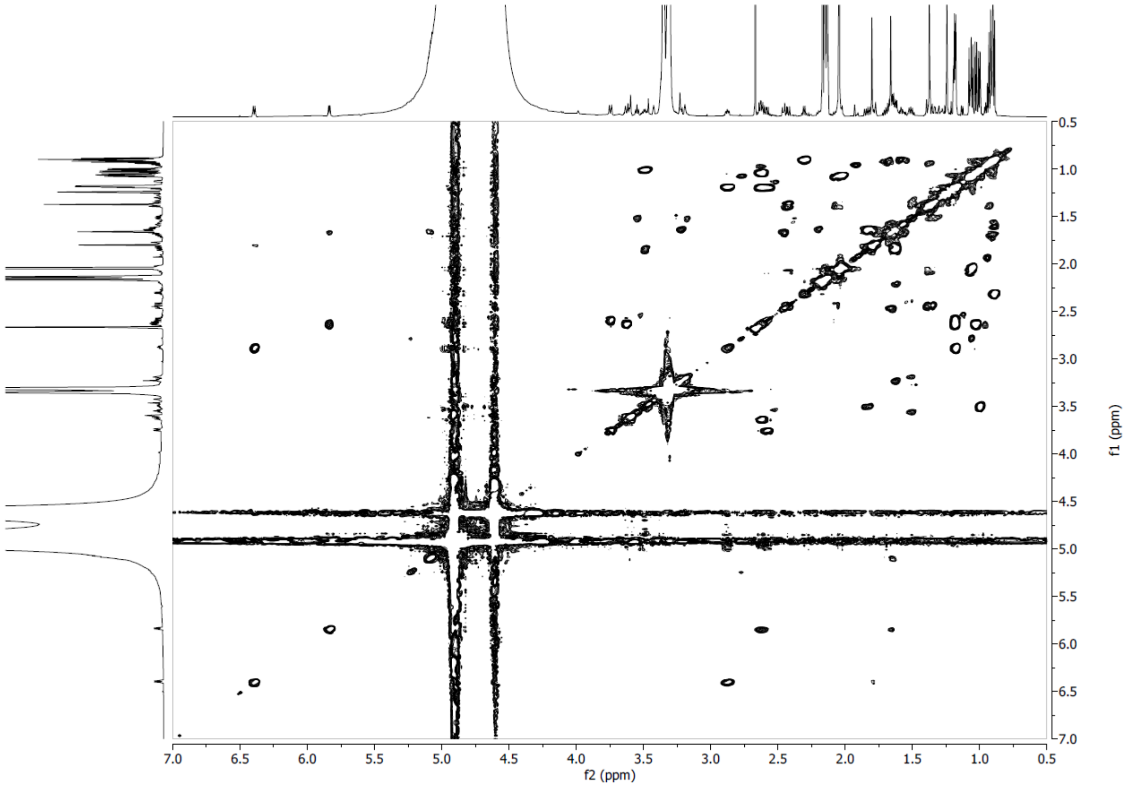

Figure S39 : COSY NMR spectrum of 6,9-hemiacetal-9-hydroxy-10,11-anhydroerythronolide B (**5.1**) and 8-*epi*-10,11-anhydroerythronolide B (**5.2**) in CD_3OD at 600 MHz.

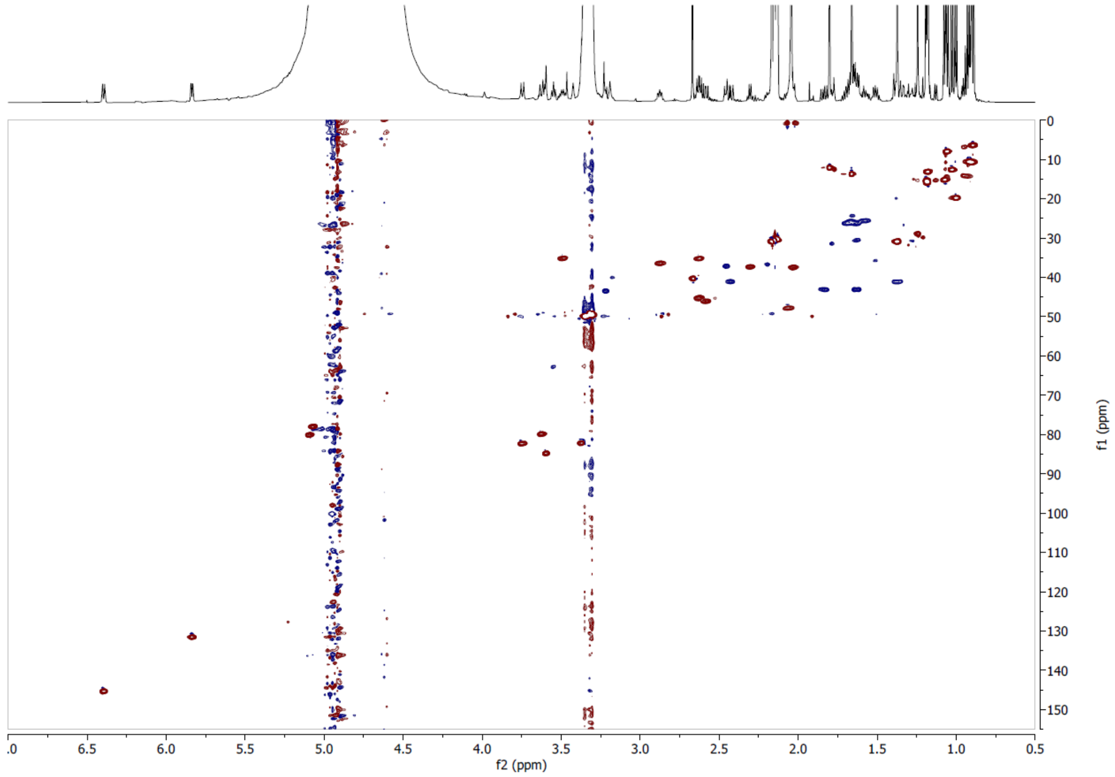

Figure S40 : Edited HSQC NMR spectrum of 6,9-hemiacetal-9-hydroxy-10,11-anhydroerythronolide B (**5.1**) and 8-*epi*-10,11-anhydroerythronolide B (**5.2**) in CD_3OD at 600 MHz.

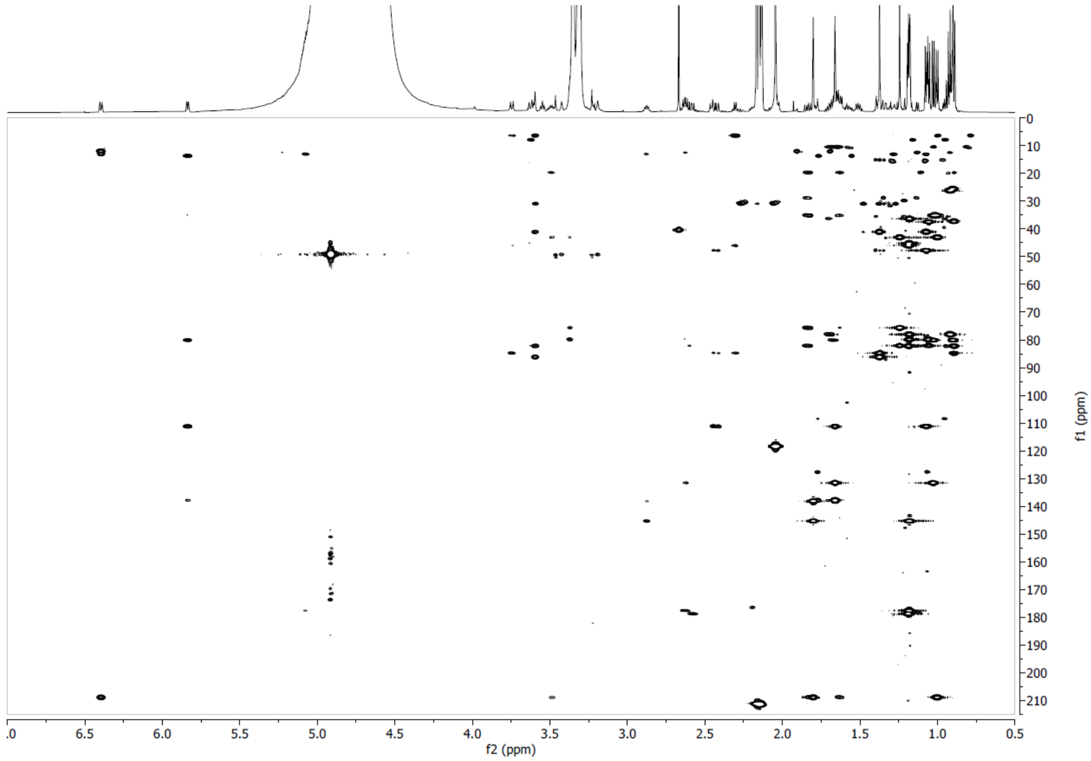

Figure S41 : HMBC NMR spectrum of 6,9-hemiacetal-9-hydroxy-10,11-anhydroerythronolide B (**5.1**) and 8-*epi*-10,11-anhydroerythronolide B (**5.2**) in CD_3OD at 600 MHz.

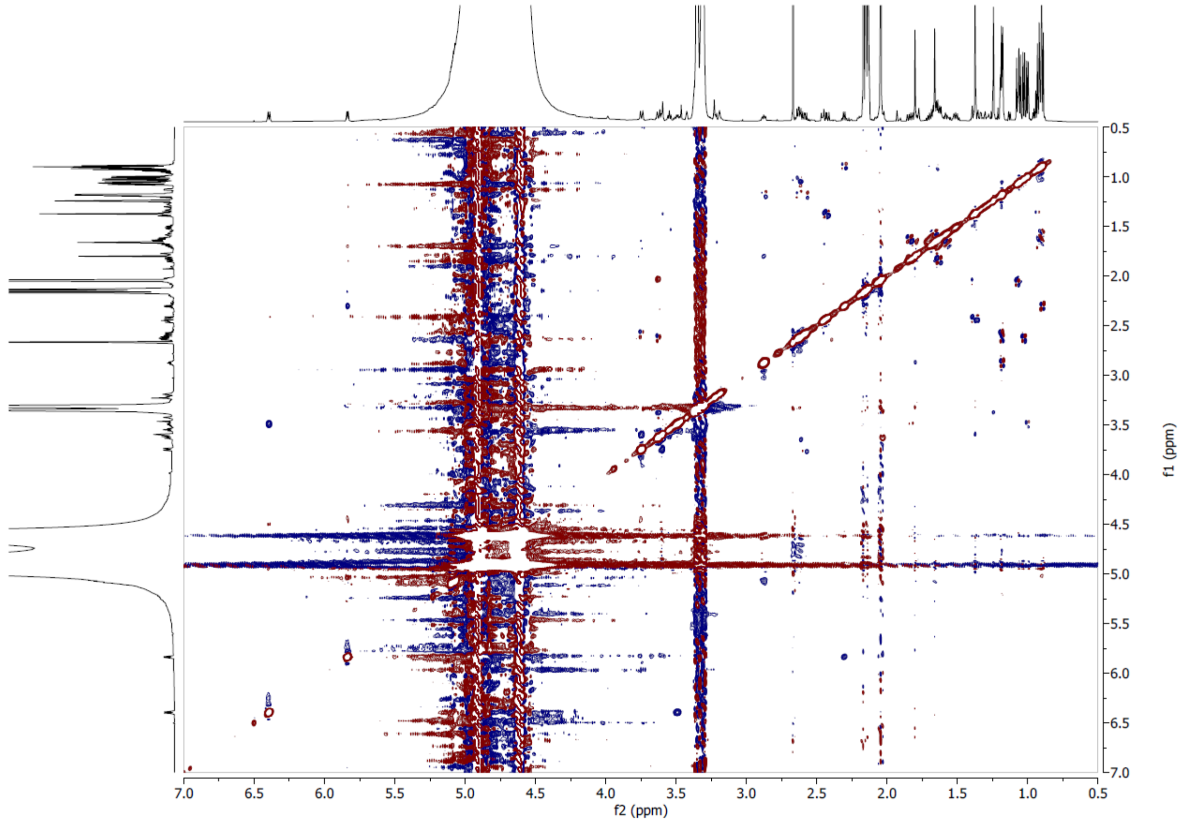

Figure S42 : ROESY NMR spectrum of 6,9-hemiacetal-9-hydroxy-10,11-anhydroerythronolide B (**5.1**) and 8-*epi*-10,11-anhydroerythronolide B (**5.2**) in CD_3OD at 600 MHz.

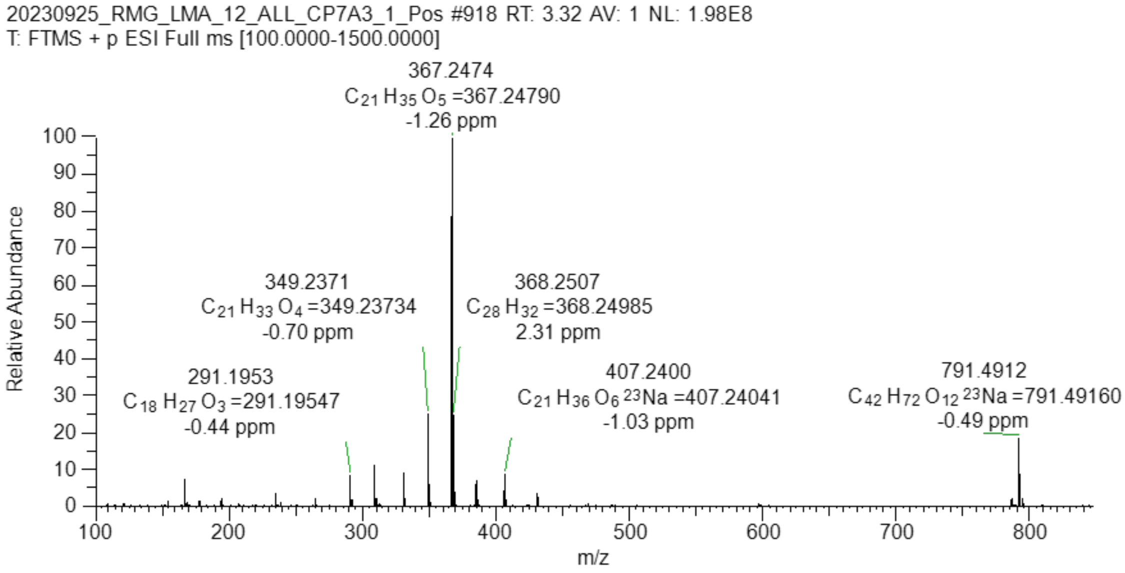

Figure S43 : ESI+-HRMS data in positive mode of 6,9-hemiacetal-9-hydroxy-10,11-anhydroerythronolide B (**5.1**) and 8-*epi*-10,11-anhydroerythronolide B (**5.2**) one year later.

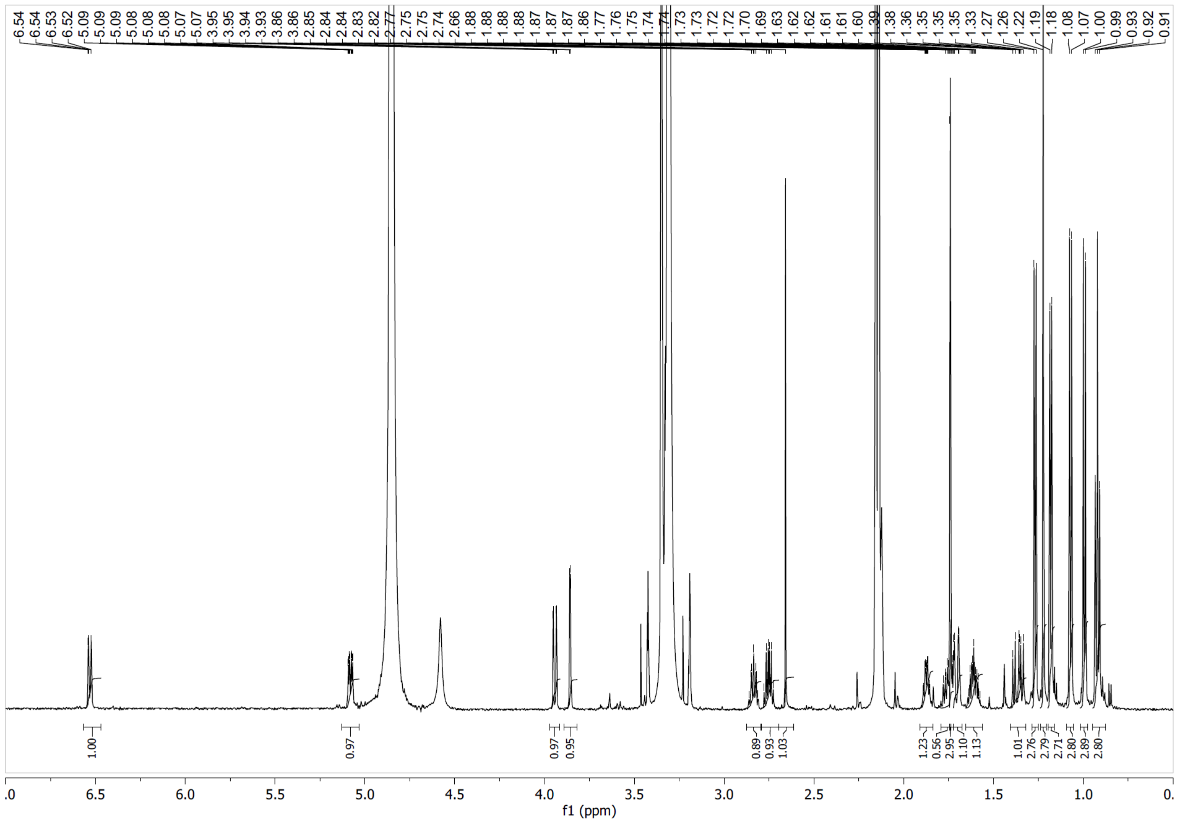

Figure S44 : ^{1}H NMR spectrum of 10,11-anhydroerythronolide B (**6**) in CD_3OD at 600 MHz.

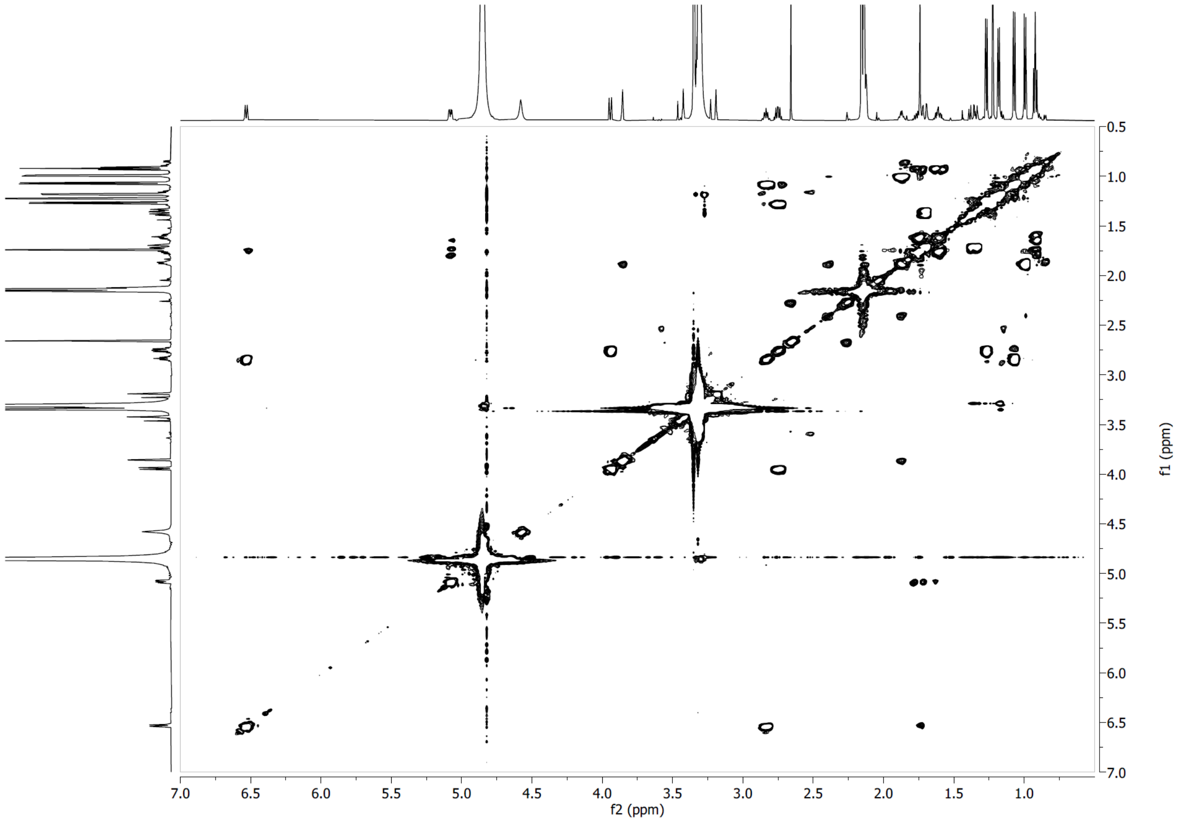

Figure S45 : COSY NMR spectrum of 10,11-anhydroerythronolide B (**6**) in CD_3OD.

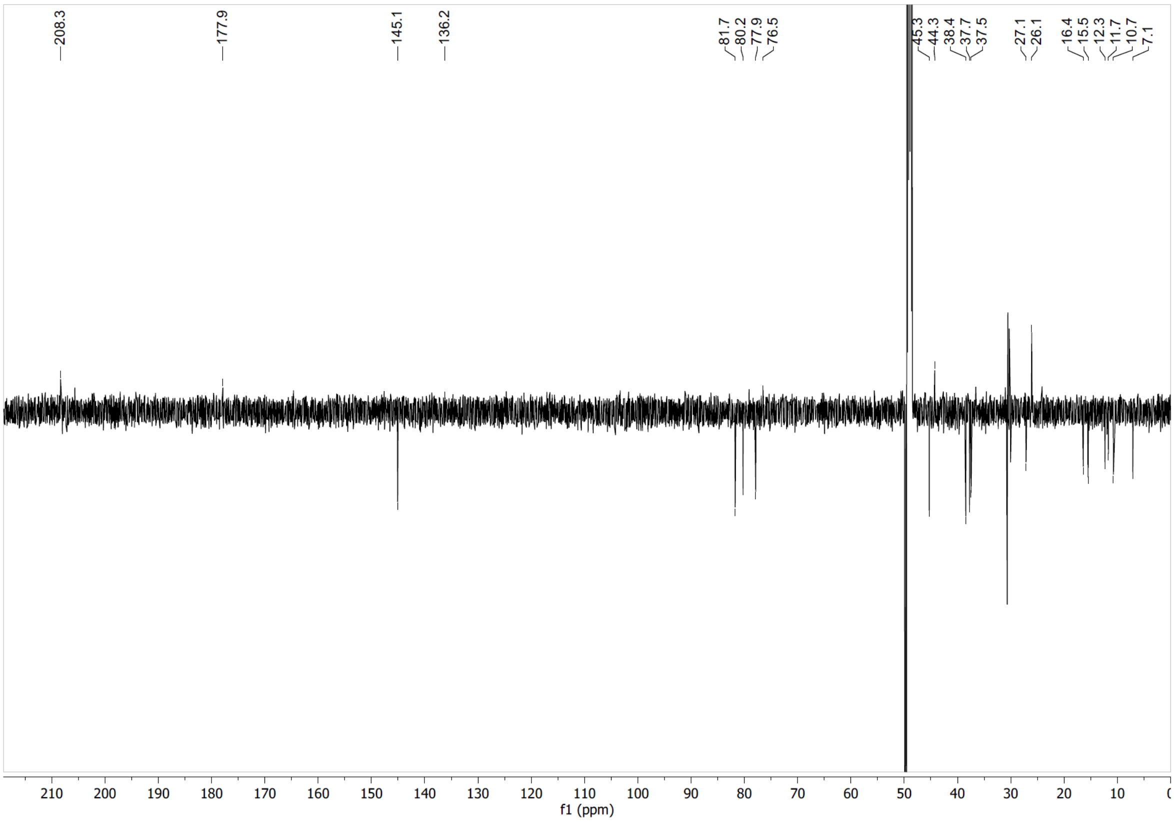

Figure S46 : ^{13}C-DEPTQ NMR spectrum of 10,11-anhydroerythronolide B (**6**) in CD_3OD at 151 MHz.

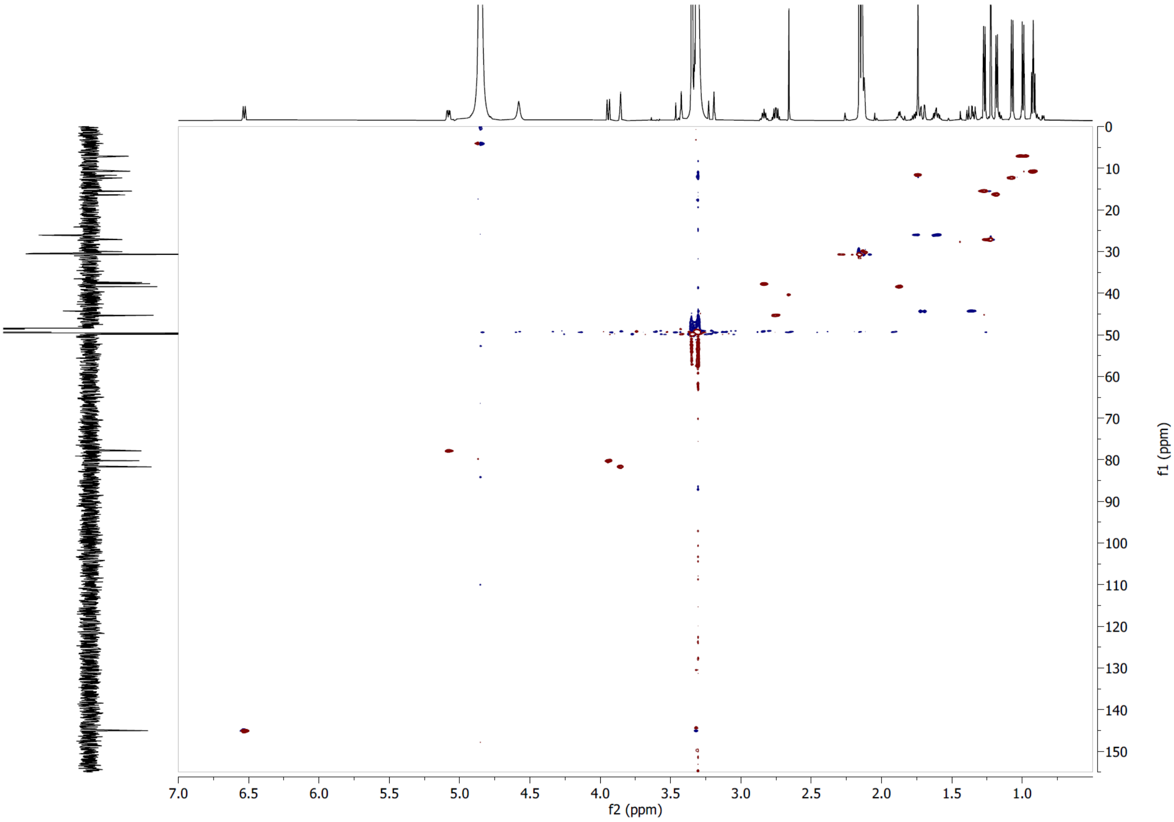

Figure S47 : Edited HSQC NMR spectrum of 10,11-anhydroerythronolide B (**6**) in CD_3OD.

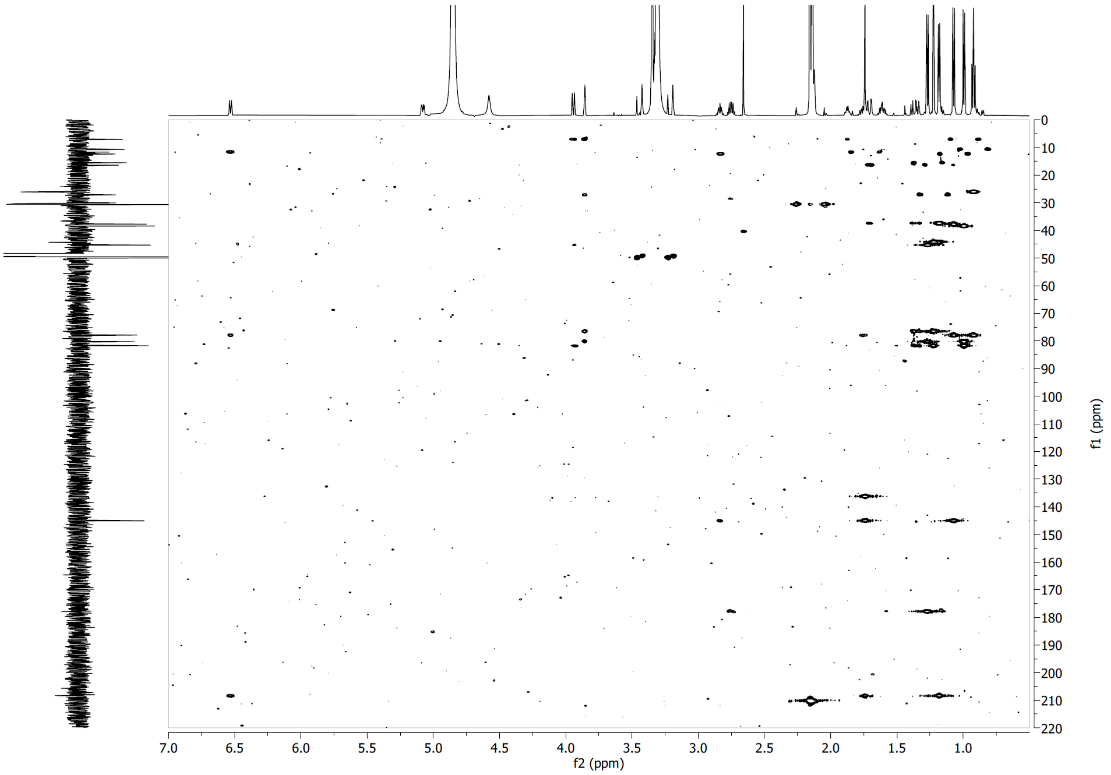

Figure S48 : HMBC NMR spectrum of 10,11-anhydroerythronolide B (**6**) in CD_3OD.

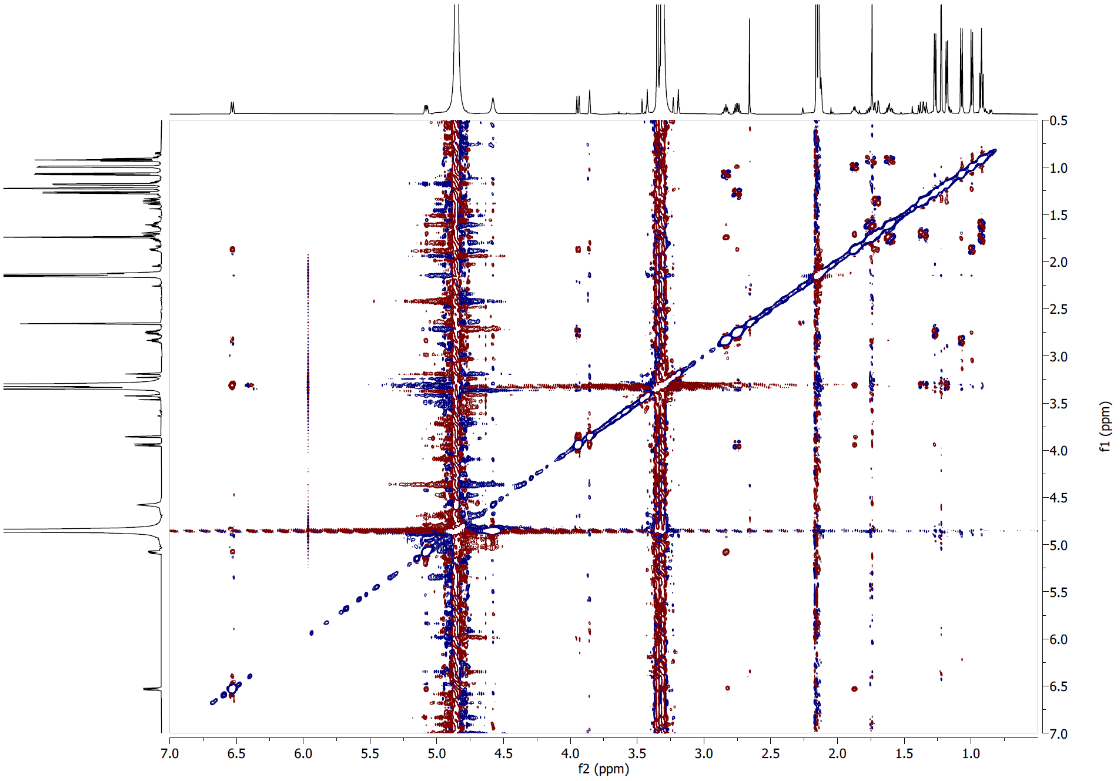

Figure S49 : ROESY NMR spectrum of 10,11-anhydroerythronolide B (**6**) in CD_3OD.

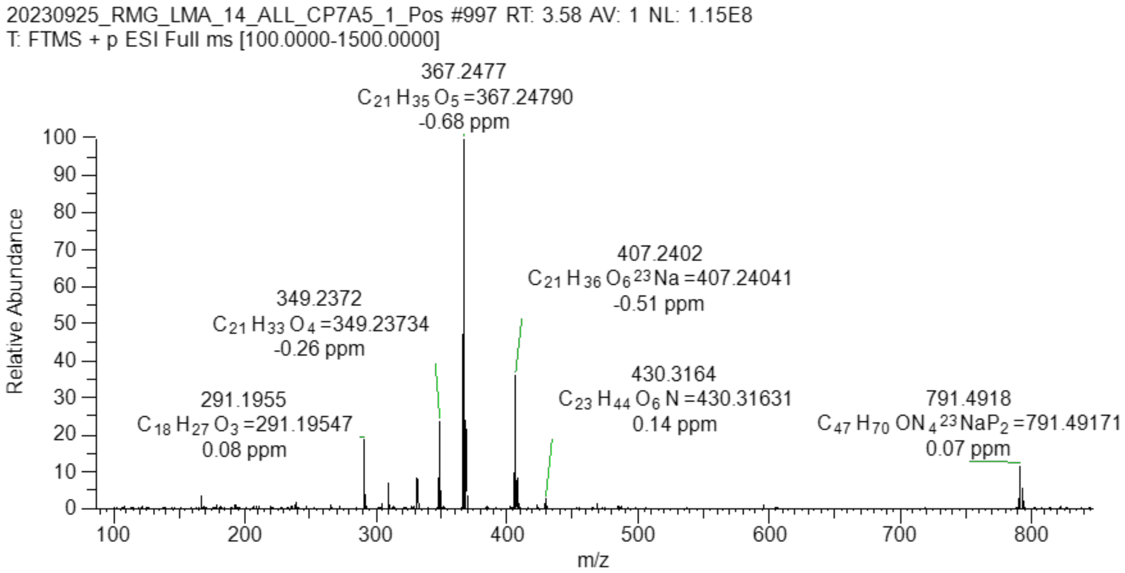

Figure S50 : ESI$^+$-HRMS data in positive mode of 10,11-anhydroerythronolide B (**6**).

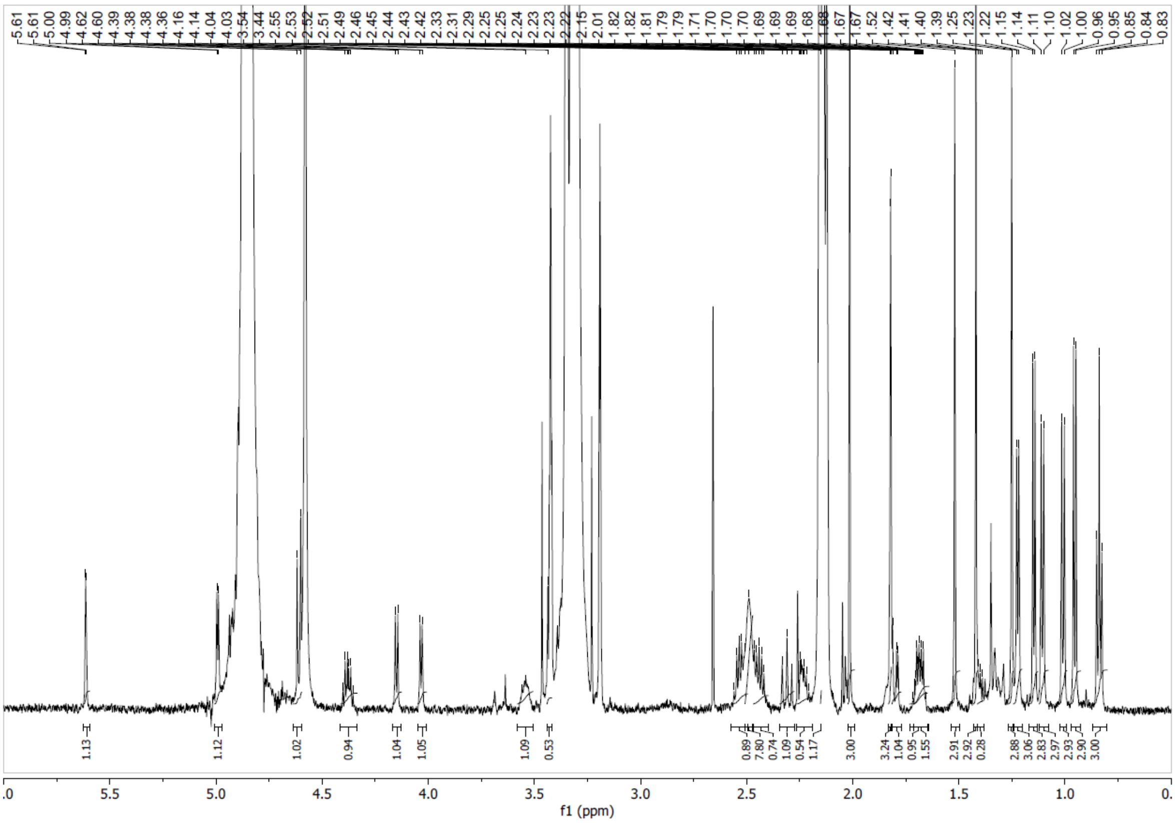

Figure S51 : ^{1}H NMR spectrum of 3'',4''-di-*O*-acetyl- 9-deoxo-6,12-dideoxy-6,9:9,12-diepoxyerythromycin D (**7**) in CD_3OD at 600 MHz.

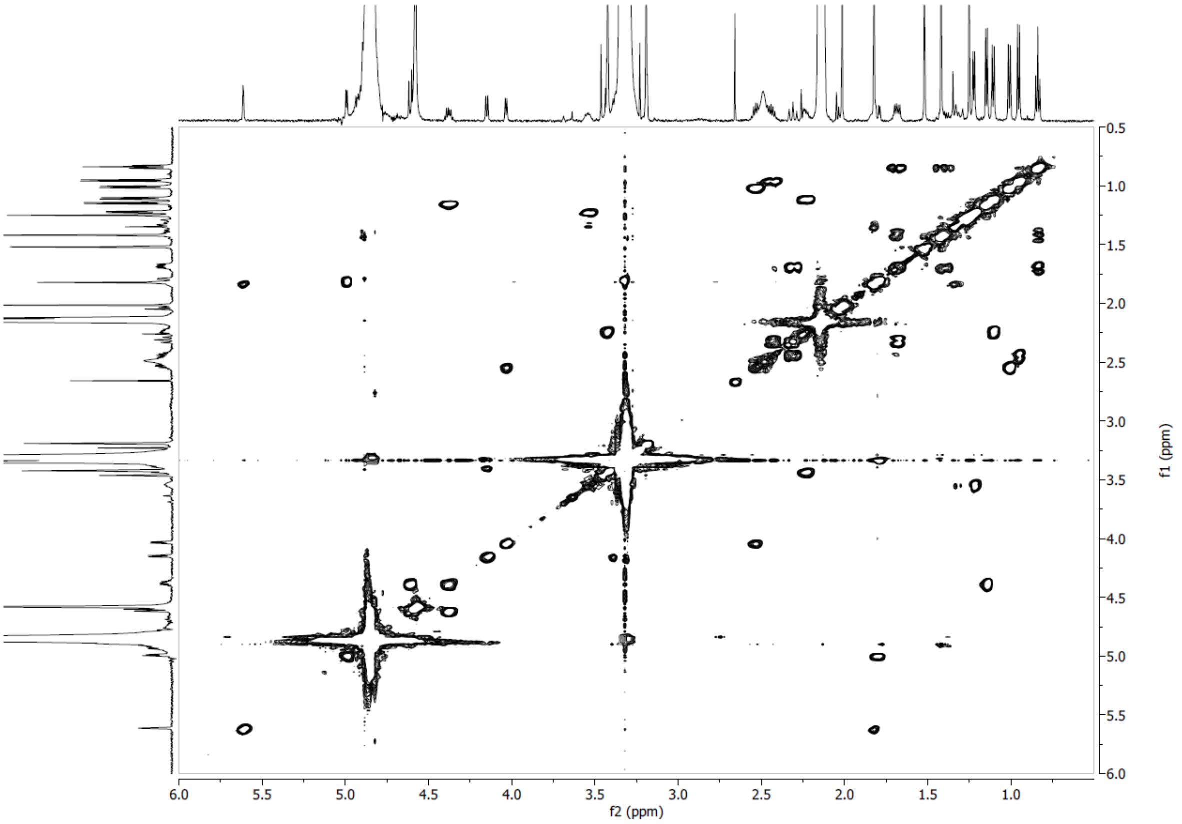

Figure S52: COSY NMR spectrum of 3'',4''-di-*O*-acetyl- 9-deoxo-6,12-dideoxy-6,9:9,12-diepoxyerythromycin D (**7**) in CD_3OD.

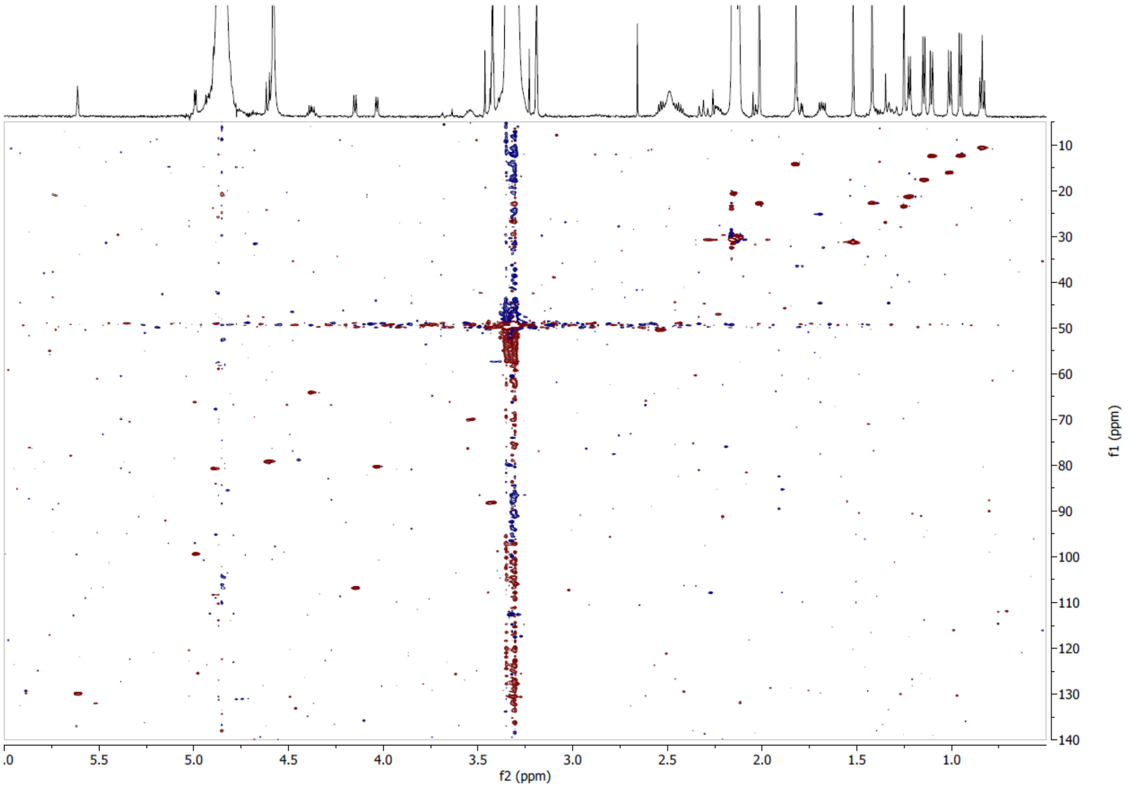

Figure S53 : Edited HSQC NMR spectrum of 3'',4''-di-*O*-acetyl- 9-deoxo-6,12-dideoxy-6,9:9,12-diepoxyerythromycin D (**7**) in CD_3OD.

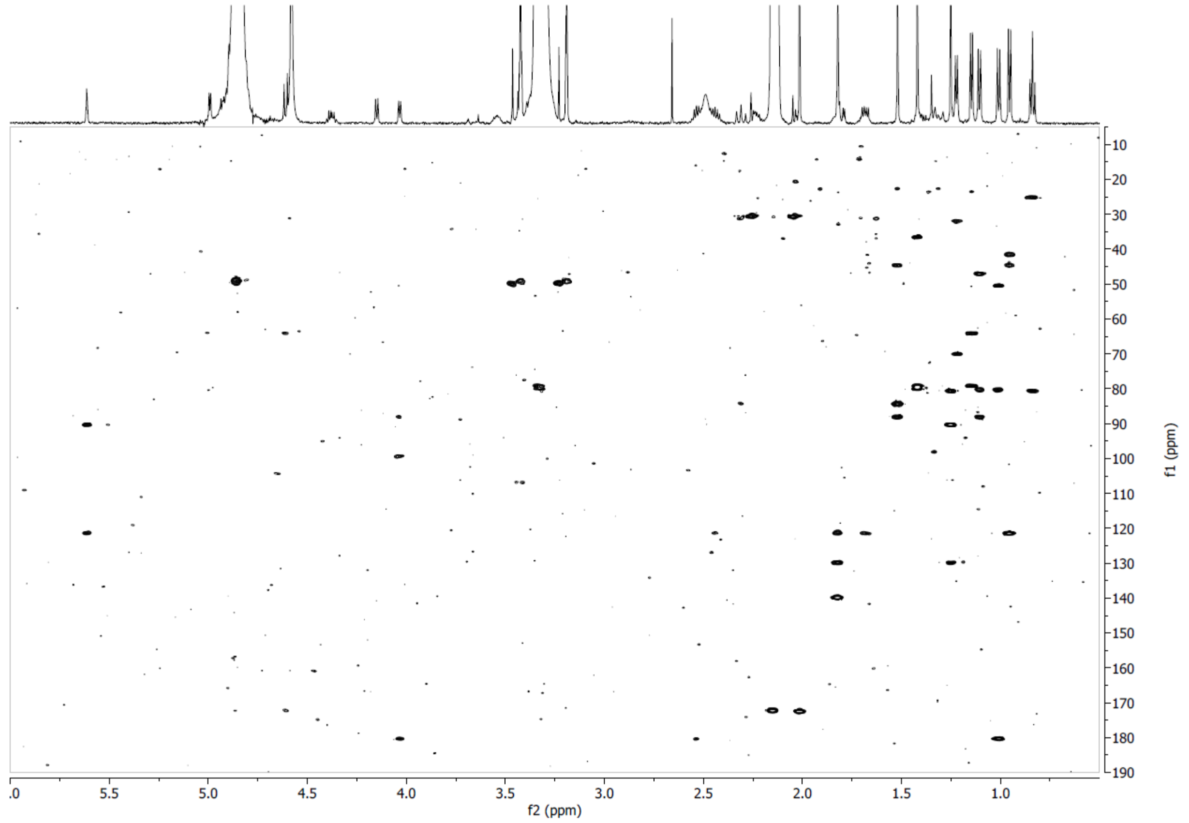

Figure S54 : HMBC NMR spectrum of 3'',4''-di-*O*-acetyl- 9-deoxo-6,12-dideoxy-6,9:9,12-diepoxyerythromycin D (**7**) in CD_3OD.

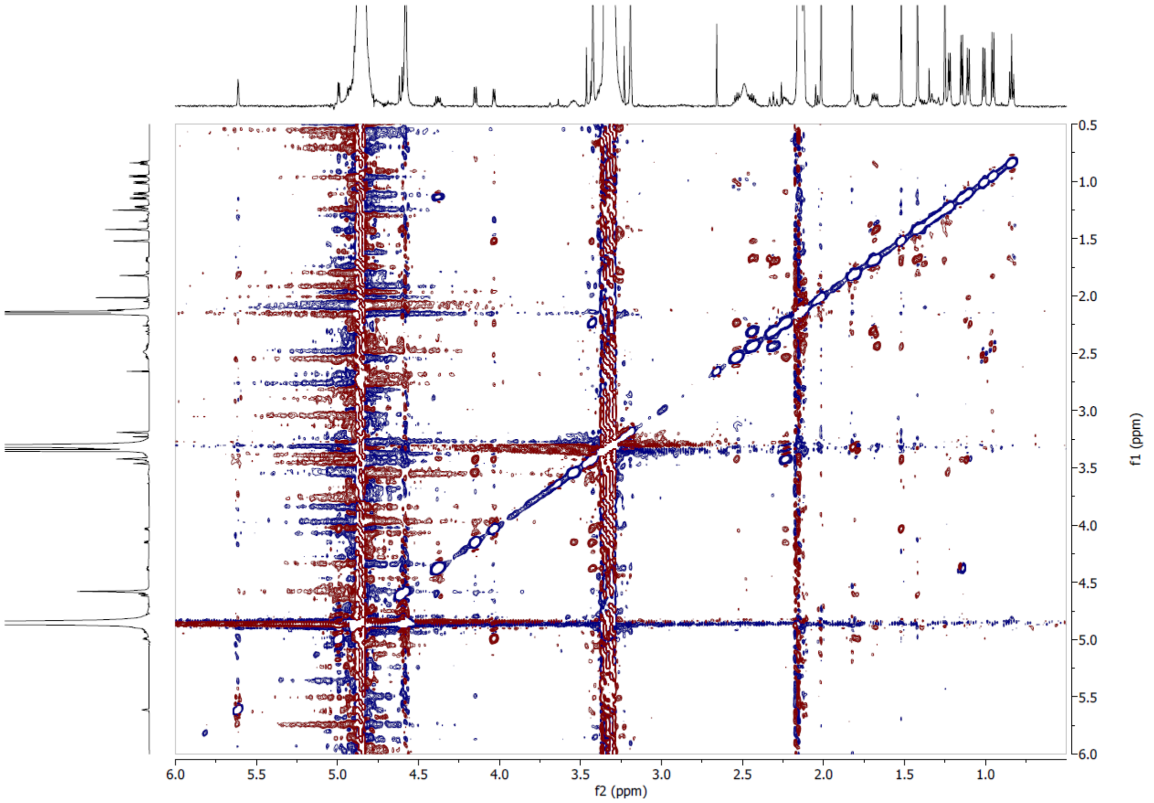

Figure S55 : ROESY NMR spectrum of 3'',4''-di-*O*-acetyl- 9-deoxo-6,12-dideoxy-6,9:9,12-diepoxyerythromycin D (**7**) in CD_3OD.

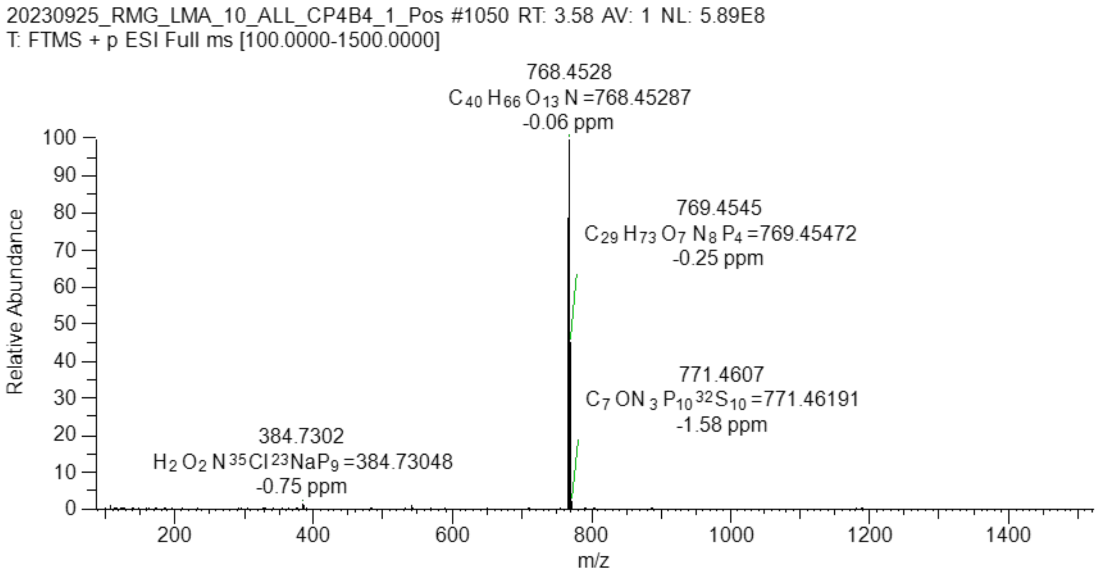

Figure S56 : ESI$^+$-HRMS data in positive mode of 3'',4''-di-*O*-acetyl- 9-deoxo-6,12-dideoxy-6,9:9,12-diepoxyerythromycin D (**7**).

Table S5: Summary table of annotations from the Ion Identity Molecular Network of *Micromonospora* sp. SH-82.

Compound ID	RT	m/z [+Adduct]	Molecular formula	Compound name or InChIKey [1,2]	Similarity [1,2]
C.1	2.01	961.5931 $[M+H]^+$	$C_{48}H_{84}N_2O_{17}$	Megalomicin C1 (compound **3**) [1,2]	81.70% [1] / 0.46 [2]
C.2	2.21	975.6086 $[M+H]^+$	$C_{49}H_{86}N_2O_{17}$	Megalomicin C2 [1]	81.49% [1]
C.3	2.34	975.6091 $[M+H]^+$			81.70% [1]
C.4	1.95	933.5988 $[M+H]^+$	$C_{47}H_{84}N_2O_{16}$	4'-Propionylmegalomicin A [1]	89.58% [1]
C.5	1.78	919.5822 $[M+H]^+$	$C_{46}H_{82}N_2O_{16}$	Megalomicin B [1]	89.97% [1]
C.6	1.61	877.564 $[M+H]^+$	$C_{44}H_{80}N_2O_{15}$	Megalomicin A [1]	92.24% [1]
C.7	1.40	733.4921 $[M+H]^+$	$C_{37}H_{68}N_2O_{12}$	9-Deoxo-9-iminoerythromycin A [1]	94.35% [1]
C.8	2.26	560.3799 $[M+H]^+$	$C_{29}H_{53}NO_9$	3-*O*-De(3-C,3-*O*-dimethyl-2,6-dideoxy-alpha-L-ribo-hexopyranosyl)-6-deoxyerythromycin [1]	83.52% [1]
C.9	2.48	704.4587 $[M+H]^+$	$C_{36}H_{65}NO_{12}$	Erythromycin D [1,2]	93.54% [1] / 0.18 [2]
C.10	2.16	720.4535 $[M+H]^+$	$C_{36}H_{65}NO_{13}$	Erythromycin C [1,2]	93.37% [1] / 0.18 [2]
C.11	3.40	369.2674 $[M-H_2O+H]^+$	$C_{21}H_{38}O_6$	6-Desoxyerythronolide B (compound **2**) [1]	56.08% [1]/ 0.17 [2]
C.12	3.01	355.2467 $[M-H_2O+H]^+$	$C_{20}H_{36}O_6$	8-Methyloleandolide [1]	58.66% [1]
C.13	3.03	399.2726 $[M+H]^+$ / 416.2990 $[M+NH_4]^+$	$C_{22}H_{38}O_5$	6,9-hemiacetal-9-*O*-methyl-10,11-anhydroerythronolide B (compound **5**)	NA
C.14	2.79	385.2561 $[M-H_2O+H]^+$	$C_{21}H_{38}O_7$	Erythronolide B (compound **1**) [1]	64.93% [1]
C.15	3.42	385.2591 $[M+H]^+$	$C_{21}H_{36}O_6$	6,9-hemiacetal-8,9-anhydroerythonolide B (compound **4**)	NA
C.16	3.59	402.2852 $[M+NH_4]^+$		10,11-anhydroerythronolide B (compound **6**)	
C.17	2.46	371.2432 $[M-H_2O+H]^+$	$C_{20}H_{36}O_7$	2-desmethyl-2-hydroxy-6-deoxyerythronolide B [1]	68.03% [1]
C.18	2.63	401.2549 $[M+H]^+$	$C_{21}H_{36}O_7$	5-deoxy-5,8-epoxy-8-epi-erythronolide B [1]	63.45% [1]
C.19	2.90	515.3200 $[M-H_2O+H]^+$	$C_{27}H_{48}O_{10}$	L-Olivosyl-erythronolide B [1]	79.85% [1]
C.20	3.16	529.3372 $[M-H_2O+H]^+$	$C_{28}H_{50}O_{10}$	3-*O*-Alpha-mycarosylerythronolide B [1]	69.72% [1]

Data from [1] SIRIUS and [2] ISDB timaR bioinformatics tools.

Table S5 : *Continued*

Compound ID	RT	m/z [+Adduct]	Molecular formula	Compound name or InChIKey [1,2]	Similarity [1,2]
C.21	3.77	269.2264 $[M-H_2O+H]^+$	$C_{20}H_{30}O$	Retinol [1,2]	78.86% [1]
C.22	2.21	287.2370 $[M+H]^+$			81.60% [1]
C.23	4.11	287.2369 $[M+H]^+$			89.34% [1]
C.24	4.31	269.2260 $[M-H_2O+H]^+$			79.84% [1] / 0.25 [2]
C.25	2.98	466.3147 $[M+H]^+$	$C_{26}H_{43}NO_6$	Glycocholic acid (D4) [1,2]	100% [1] / 0.21 [2]
C.26	3.58	450.3218 $[M+H]^+$	$C_{26}H_{43}NO_5$	Glycocholic acid (D6) [1,2]	98.64% [1] / 0.37 [2]
C.27	5.44	300.2897 $[M+H]^+$	$C_{18}H_{37}NO_2$	Palmitoylethanolamide-D4 [1,2]	100% [1] / 0.11 [2]
C.28	1.37	180.1020 $[M+H]^+$	$C_{10}H_{13}NO_2$	N-Acetyltyramine [1,2]	99.43% [1] / 0.52 [2]

Data from [1] SIRIUS and [2] ISDB timaR bioinformatics tools.

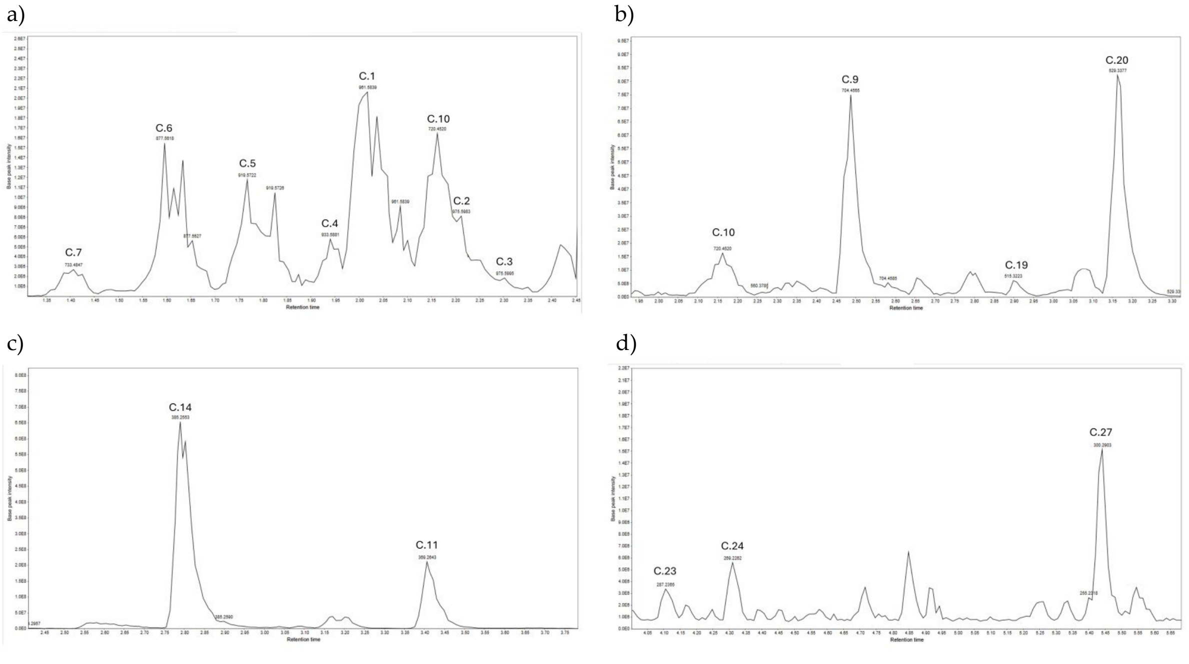

Figure S57 : Different EICs representing the annotated compounds. a) EIC 1000-700 *m/z*, RT: 1.3-2.4 min; b) EIC 750-500 *m/z*, RT: 1.9-3.3 min; c) EIC 450-350 *m/z*, RT: 2.4-3.7 min; d) EIC 300-100 *m/z*, RT: 4.0-5.7 min.

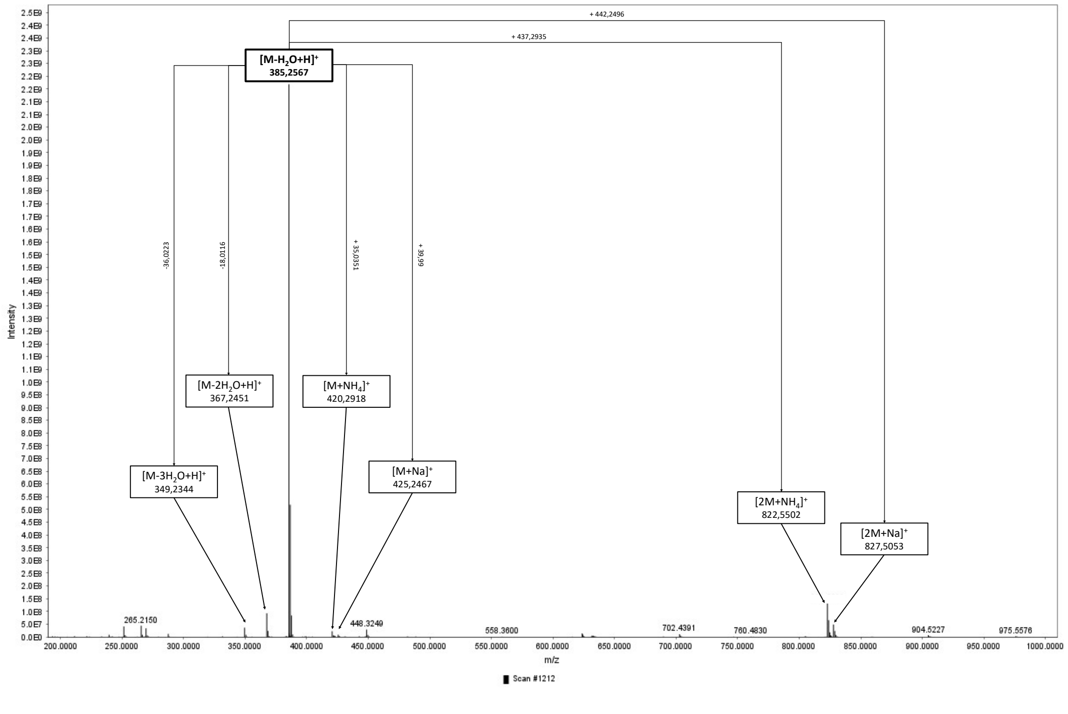

Figure S58 : ESI$^+$-HRMS data in positive mode of erythronolide B (**1**) (*m/z* 385.2561 [M-H_2O+H]$^+$, $C_{21}H_{38}O_7$) with the various adducts identified.

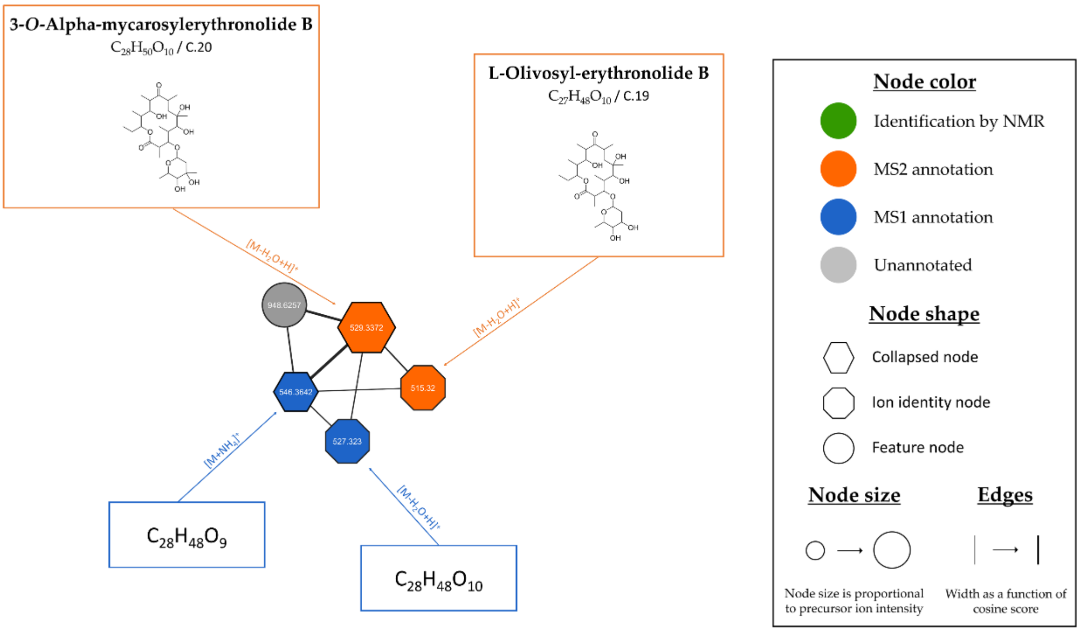

Figure S59 : Zoom-in on other clusters with MS2 annotation. Nodes are colored according to identification level : green for metabolites isolated and identified by NMR, orange for features annotated by MS2 indicating potential identification by bioinformatics tools, blue for features annotated by MS1 revealing identification of the adduct and associated molecular formula and grey for unannotated features.

Query sequence, Region 11, T1PKS, Location: 3,472,845 - 3,539,791 nt. (total: 66,947 nt)

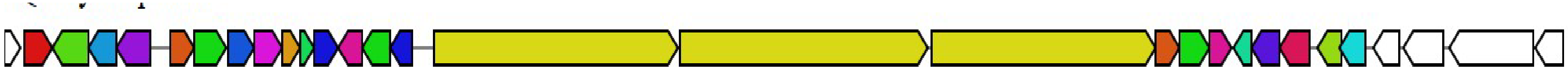

BGC0000092: megalomicin A/megalomicin B/megalomicin C1/megalomicin C2 (94% of genes show similarity), Polyketid

BGC0000092 from Micromonospora.megalomicea : megalomicin A/megalomicin B/megalomicin C1/megalomicin C2 (94% of genes show similarity)

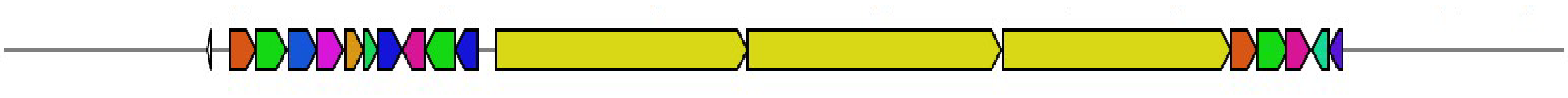

Figure S60 : Similarity between the BGC from *Micromonospora* sp. SH-82 genomic analysis (top) and the BGC0000092 from *Micromonospora megalomicea* responsible for megalomicin biosynthesis (bottom).

Table S6 : Batch mode used for data processing with MZmine 3 software.

1) Mass detection
Scan MS1 centroid ; noise level : $5^{E}5$
Scan MS2 centroid ; noise level : $1^{E}0$

2) ADAP Chromatogram Builder
Min group size in # of scans : 3
Group intensity threshold : $5^{E}5$
Min highest intensity : $1^{E}6$
Scan to scan accuracy : 0.0050 Da or 20 ppm

3) ADAP feature resolver
MS/MS scan pairing: retention time (RT) tolerance : 0.2 min
MS1 to MS2 precursor tolerance : 0.0070 Da
S/N threshold : 10
Minimum feature height : $1^{E}6$
Coefficient/area threshold 70
Peak duration range : 0-0.40 min
RT wavelet range: 0-0.05 min

4) 13C isotope filter
m/z tolerance : 0.007 Da
RT tolerance : 0.07 min
Maximum charge : 2
Representative isotope most intense

5) Join aligner
m/z tolerance : 0.007 Da
Weight for *m/z* : 80
RT tolerance : 0.1 min
Weight for RT : 40

6) Feature list blank subtraction
Minimum # of detection in blanks : 1
Fold change increase : 100%

7) metaCorrelate
RT : 0.05 min
Min height : $1^{E}6$
Correlation grouping
Feature height correlation

8) Ion identity molecular networking
m/z tolerance : 4 ppm

9) Export feature list GNPS
Feature intensity : peak area
CSV export simple

Table S7 : Parameters used to create IIMNs on the GNPS platform.

1) Basic options
Precursor Ion Mass Tolerance : 0.02 Da
Fragment Ion Mass Tolerance : 0.02 Da

2) Advanced network options
Min pairs cos : 0.70
Network TopK : 10
Minimum Matched Fragment Ions : 12

3) Advanced library search options
Library Search Min Matched Peaks : 6
Score Threshold : 0.7
